# Multicolumn Two-Dimensional Liquid Chromatography Screening Platform for Stereopeptidomics and Application to Antimicrobial Peptide Polyene and Lipopeptide

**DOI:** 10.1021/acs.analchem.5c02658

**Published:** 2025-06-24

**Authors:** Cornelius Knappe, Simon J. Jaag, Taulant Dema, Ruslan Jaufmann, Stephan Buckenmaier, Harald Gross, Stephanie Grond, Michael Lämmerhofer

**Affiliations:** † Pharmaceutical (Bio-)Analysis, Institute of Pharmaceutical Sciences, 9188 University of Tübingen, Auf der Morgenstelle 8, Tübingen 72076, Germany; ‡ Institute of Organic Chemistry, University of Tübingen, Auf der Morgenstelle 18, Tübingen 72076, Germany; § R&D Liquid Phase Division, Agilent Technologies Deutschland GmbH, Hewlett-Packard-Str. 8, Waldbronn 76337, Germany; ∥ Pharmaceutical Biology, Institute of Pharmaceutical Sciences, University of Tübingen, Auf der Morgenstelle 8, Tübingen 72076, Germany

## Abstract

Therapeutic peptides are a rapidly growing field in research and drug development. While the majority of natural and synthetic therapeutic peptides have l-amino acids as building blocks, d-amino acid-containing peptides are found frequently in nonribosomal peptides or can be formed during peptide synthesis by epimerization. Thus, analytical methods are needed for the quality control of stereointegrity and the determination of absolute configurations. Enantioselective amino acid analysis following complete hydrolysis is indispensable in the field but leads to the loss of sequence information, i.e., the position of d-amino acids can no longer be unambiguously assigned. Here, we propose a multicolumn two-dimensional liquid chromatography-tandem high-resolution mass spectrometry (2D LC-HRMS) platform with multiple reversed-phase type columns (C18, charged surface hybrid C18, mixed-mode C18 AX) in the first dimension (^1^D) and multiple chiral columns in the second dimension (^2^D) (teicoplanin, teicoplanin aglycone, crown ether, and zwitterionic quinine and quinidine carbamate-based chiral stationary phases). It allows the combination of distinct ^1^D columns (for peptide epimer/diastereomer separations) and ^2^D columns (for peptide enantiomer separations), enabling the full resolution of complex peptide stereoisomer mixtures. The utility of this 2D-LC platform for peptide analyses was demonstrated for a tetrapeptide amide from an antimicrobial peptide polyene natural product and a lipopeptide, digested into dipeptides for middle-down/middle-up stereoselective peptide analysis. Multiple heart-cutting and selective comprehensive 2D-LC, respectively, with active solvent modulation and sequential window acquisition of all theoretical fragment ion spectra mass spectrometry (SWATH-MS) enabled the full separation of all stereoisomers and the clarification of the configurations of all sample peptides. Such a 2D-LC-HRMS screening platform can be valuable as an efficient and fast generic approach for streamlining method development in the pharmaceutical industry.

## Graphical abstract

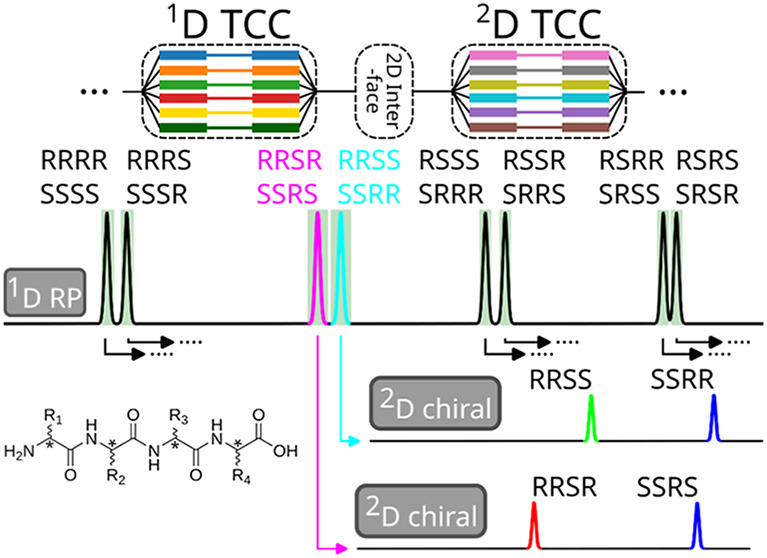

## Introduction

Peptides are target commodities of increasing importance in analytical chemistry due to their major roles as bioactive, therapeutic, and diagnostic peptides, as disease biomarkers, and food components. Their stereochemistry is one of the critical structural attributes and thus must be characterized and controlled. It is a challenging task as there is a lack of generic analytical solutions.^1,2^ Liquid chromatography (LC)^3^ and capillary electrophoresis (CE) assays^4^ have been methods of first choice in the past. Nowadays, ion-mobility-mass spectrometry (IM-MS) (and its coupling with LC and CE) is gaining impetus in this field, especially for peptide epimer/diastereomer analysis,^5–8^ yet it fails to separate peptide enantiomers. In several applications, distinct tools are required, which can address both peptide epimers/diastereomers and enantiomers separation. In general, for peptides made from l-amino acids and with increasing length, the analysis of peptide diastereomers is of higher relevance than peptide enantiomer separations. However, natural peptides often contain d-amino acids, and from a strict quality assurance point of view, assay specificity testing requires selectivity for all stereoisomers. This, however, is illusive on the intact level of peptides with more than four stereogenic centers, and other strategies are needed.

From a biological viewpoint, biogenic peptides usually result from ribosomal peptide synthesis involving l-amino acids (l-AAs). Here, peptide isomerism can originate from sequence scrambling, positional isomerism of post-translational modifications (PTMs), isomeric amino acid residues (Leu vs Ile; Thr vs allo-Thr, and Thr vs homo-Ser), or so-called zero-mass shift PTMs.^5,9^ The current study is focused on d-AA-containing peptides that can originate from different pathways. They can either emerge from ribosomal peptides by post-translational modification (so-called ribosomally synthesized and post-translationally modified peptides, RiPPs).^10^ Irreversible, post-translational epimerization in RiPPs is achieved by radical *S*-adenosyl-l-methionine (SAM) enzymes.^10^ Furthermore, d-AA-containing peptides can also result from ribosomal all-l-peptides by the above-mentioned zero-mass shift PTMs, e.g., Asp/isoAsp isomerization,^9^ spontaneous isomerization (epimerization at single AAs e.g., Ser), or enzymatic isomerization by peptide isomerases.^11,12^ l-Asp and l-Asn residues in peptides can undergo l-succinimide formation and further epimerization from l-succinimide to d-succinimide via the enol intermediate. Reversible hydrolytic ring-opening of l- and d-succinimide at the backbone amide group leads to l- and d-isoAsp-containing peptides, and hydrolytic ring-opening at the side chain amide to the corresponding l- and d-Aspcontaining peptides. Such Asp/isoAsp isomerization and epimerization occur in long-lived peptides and prevent their digestion by the proteases of the lysosome.^13^ Spontaneous stereoinversion is also observed in Ser residues of peptides (presumably via an enol intermediate), and like Asp/isoAsp isomerization/epimerization, it has been found in long-lived proteins, e.g., amyloid-β (Aβ), as present in the plaques of Alzheimer′s disease.^3^ Other age-related diseases are associated with isomerization/epimerization (of Asp/Asn/Ser) as well, such as cataract (Asp/isoAsp isomerizations of the lens proteins αA- and αB-Crystallin).^14^ Single amino acid stereoconfigurations are intentionally epimerized by peptide isomerases in many venoms and in signaling neuropeptides, e.g., in crustaceans, being a requirement for their bioactivities.^11,15,16^ On the other hand, natural peptides from microbial nonribosomal peptide synthetases (NRPSs) are rich in d-AAs.^17^ NRPSs are multimodular enzyme complexes, which generate peptides (particularly *M*_r_ ∼ 200–2000 Da) by converting amino acids into d-configuration by epimerase domains in the growing peptide chain.^17^ Numerous nonribosomal peptides have made it to the drug market (e.g., cyclosporine A, teicoplanin, daptomycin, caspofungin).^17^

Peptide research is nowadays booming, not least due to increasing numbers of peptide therapeutics and the blockbuster status of several therapeutic peptides, like semaglutide or tirzepatide.^18^ They are typically prepared by both chemical synthesis and biological processes (recombinant technology comprising both ribosomally and nonribosomally synthesized peptides). Since therapeutic peptides often mimic their endogenous analogues, they typically contain l-AAs. To optimize their pharmacodynamic and pharmacokinetic properties as well as drug delivery, modifications are often introduced, ranging from genetic code expansion through introducing noncanonical AAs, conjugations, chelators like DOTA (2,2′,2″,2‴-(1,4,7,10-Tetraazacyclododecane-1,4,7,10-tetrayl)­tetraacetic acid for diagnostic and theranostic peptides), and non-natural amino acid substitutions. Following the latter concept, the replacement of l-AAs by d-AAs is a successful strategy to increase protease stability and has been incorporated, e.g., for octreotide, a synthetic somatostatin analog, and many gonadotropin-releasing hormone analogs, such as leuprorelin or abarelix. d-Amino acid-containing peptides can be formed during peptide synthesis due to the isomerization of single amino acid residues. Direct enolization (of FMOC (fluorenylmethoxycarbonyl)-protected AAs), oxazolone (azalactone) formation, and a ketene mechanism have been suggested as isomerization mechanisms.^19,20^ Since the bioactivity of peptides is strongly related to their correct stereochemistry, testing the stereochemical integrity of synthetic peptides is therefore an integral part of therapeutic peptide quality control strategies. Most importantly, it is also required to control the enantiomeric purity of the starting AAs to the highest possible degree. Spontaneous isomerizations during shelf life, as described above for Asp, Asn, and Ser, are also of concern for synthetic peptides.

The standard approach for characterizing and controlling the stereochemical integrity of peptides is to fully hydrolyze them (in D_2_O/DCl) followed by enantioselective amino acid analysis (eAAA) by LC-MS, GC-MS, or 2D-LC-MS.^21–25^ Unfortunately, the sequence information is lost during hydrolysis, and only sum d-AA information is obtained for each AA but no information on their location within the peptide chain (see Figure 1). On the other hand, it may be challenging to find zero-mass shift PTMs just by MS, IM-MS, or LC-MS in a long peptide sequence, especially under the constraint that it is impossible to have all realistic peptide stereoisomers available as a reference for validation of structural annotations. Hence, enzymatic^2,26,27^ and nonenzymatic^26^ partial hydrolysis to smaller, so-called peptide building blocks, combined with stereoselective peptide analysis, can be an effective approach toward characterization of d-AA-containing peptides. This approach is termed enantioselective peptide building block analysis (ePBBA). The development of specific methodologies for each new peptide to be characterized for its d-AA content and composition is painstaking and time-consuming. For this reason, the pharmaceutical industry seeks to establish generic platform technologies.^28–30^ 2D-LC has attracted greater attention in this context as a multiattribute method. 2D-LC platforms for peptide therapeutics characterization have been previously established.^31–33^ Yet, there has been no specific focus in these studies on peptide stereoisomers. On the other hand, a few studies elucidated platform technologies toward a generic approach to the characterization of d-AA-containing peptides.^2,27,34^ Sweedler and co-workers proposed a 3-step d-AA-containing neuropeptide discovery funnel in which first an aminopeptidase is employed for screening of peptides resistant to digestion, indicating the presence of a d-AA; second, the corresponding enzyme-resistant candidate d-AA-containing peptide is isolated and the presence of the d-AA confirmed after full hydrolysis in D_2_O/DCl by eAAA using Marfey’s reagent and achiral RPLC; and third, the putative d-AA-containing peptide is then synthesized, and its retention is compared with the endogenous peptide to confirm its structure.^2^ Armstrong and co-workers employed digestion by carboxypeptidase Y to eliminate all-l-AA-containing peptides and C-terminal l-AAs in d-AA-containing peptides to afford C-terminal d-AA-peptides, which can be effectively bound by teicoplanin-SPE and LC, respectively, which in turn possess preferential affinity for peptides with C-terminal d-AAs. This approach facilitates the concentration and identification of d-AA-containing peptides.^27^ Recently, it was demonstrated as proof of principle that nanopore technology, i.e., nanopore currents, can distinguish between stereoisomeric peptides, not distinguishable by conventional MS analysis, down to individual D-AA substitutions in small peptides.^34^ However, this technology is not yet broadly applicable.

**Figure 1:**
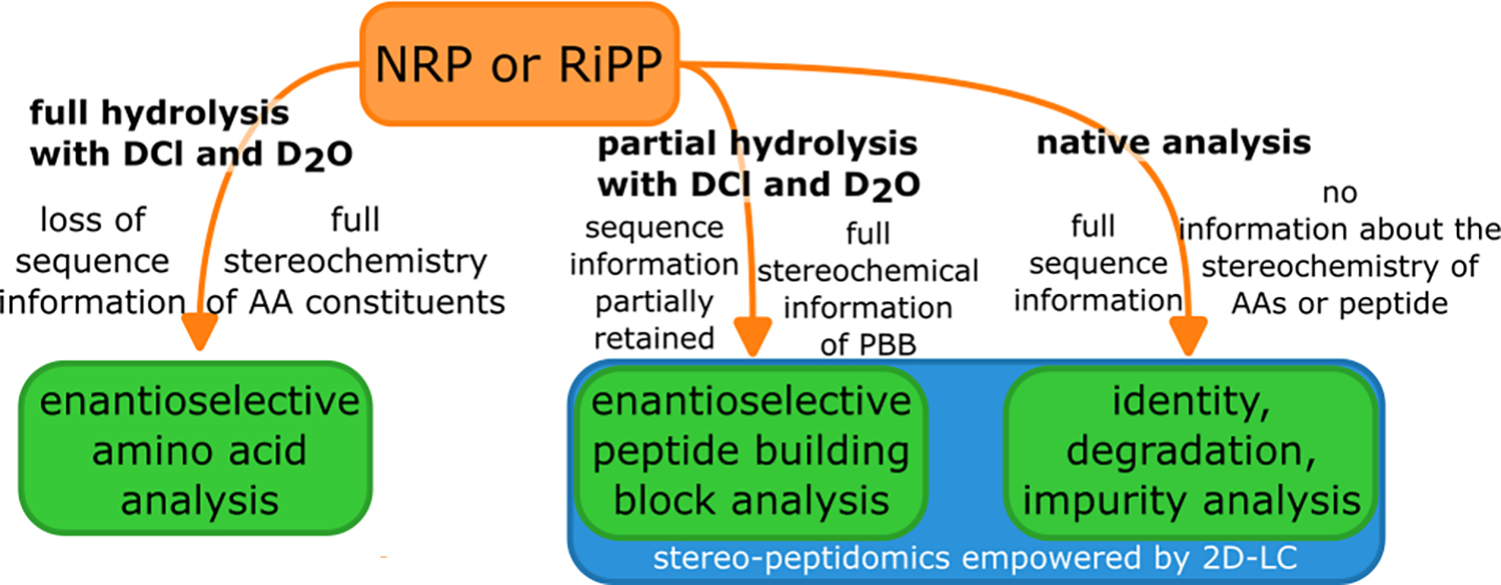
Systematic approach to get full stereochemical information on peptides. The combination of all three methods is necessary.

This study presents an automated multicolumn 2D-LC platform as a generic solution for stereoselective peptide analysis. The platform combines up to six different achiral RP-type columns in the first dimension (^1^D), up to six enantioselective columns with various types of chiral stationary phases (CSPs) in the second dimension (^2^D), and UV detection (^1^D and ^2^D) as well as QTOF-MS (^2^D) for complementary detection. The platform is designed for stereopeptidomics, including both 2D ePBBA in the range of di-, tri-, and tetrapeptides and native 2D stereoselective peptide analysis, providing identity (fully sequence information), degradation, and impurity analysis. The concept and general feasibility are demonstrated by the application to a nonribosomal peptide polyene and a nonribosomal lipopeptide.

## Experimental Section

### Materials

Epifadin-derived tetrapeptide amide samples were obtained from a previous study.^35^ Lipopeptide samples were provided by the research group of H. Gross. Individual peptide stereoisomer standards were synthesized by solid-phase or liquid-phase peptide synthesis (SPPS or LPPS) as described in Supporting Information note S1. For columns and other materials, see Supporting Information note S2.

### Instrumentation and Software

LC separations were performed by using an Agilent Technologies (Waldbronn, Germany) 1290 Infinity 2D-LC system (Figure 2). The system consisted of two 1290 Infinity II High Speed (binary) pumps (G7120A), a 1290 Infinity II Multisampler (MLS) (G7167B), two 1290 Infinity II Multicolumn thermostat compartments (G7116B), two 1290 Infinity II diode array UV absorbance detector (DAD) (G7117B), and three 1290 Infinity II Valve Drives (G1170A). Both ^1^D and ^2^D pumps were utilized with a 100 μL JetWeaver. Max-Light Cells (G4212–6008) with 10 mm path length and *V*(σ) = 1 μL were used. The ^1^D flow cell was protected by a pressure relief kit (G4236–60010). The interface connecting the ^1^D and ^2^D consisted of three valve drives, which were equipped with two deck valve heads and an ASM valve head (G4243A and G4242A) to enable multiple-heart-cutting (MHC) 2D-LC and sLCxLC 2D-LC with two times six 40 μL sample loops. In addition, column selector valve heads (G4234C) were installed in both ^1^D and ^2^D multicolumn thermostat compartments (MCT), implementing the option for column screening. The 2D detector was either the second 1290 Infinity II DAD detector or the mass spectrometer. In the case of 2D-LC-MS, the HPLC system was coupled to a Sciex (Concord, Ontario, Canada) CDS (calibrant-delivery-system) and further to a Sciex TripleTOF 5600+ system with DuoSpray source operated in electrospray ionization mode (TurboIonSpray) (see Supporting Information note S2 for more information). The HPLC system was controlled using Agilent OpenLab CDS ChemStation Edition Rev.C.01.10, and the mass spectrometer was controlled by Sciex Analyst TF 1.8.1. The 2D-LC system was running fully automated. A start signal was transferred over the ERI (Enhanced Remote Interface) cable connected from the Agilent multisampler to the mass spectrometer, and runs stopped according to their set run time in the respective 2D-LC and MS methods. No solvent selection valves were used herein; hence, automated screening of multiple 2D-LC runs was restricted to the same gradient mobile phase systems. Prior to the 2D-LC experiments, chiral 1D-LC separations for preoptimization were performed on an LC system, consisting of an Agilent 1290 Infinity High Speed (binary) pump, an Agilent 1200 thermostated column compartment (G1316A), and a PAL HTC-xt autosampler (CTC Analytics AG, Zwingen, Switzerland). This system was also coupled to a CDS and further to a Sciex TripleTOF 5600+ with DuoSpray source operated in electrospray ionization mode. Both LC and MS were controlled by Sciex Analyst TF 1.8.1. Sciex PeakView 2.2 and Agilent OpenLab CDS ChemStation Edition Rev.C.01.10 were used for data analysis, and different Python packages were used for data visualization (for details, see Supporting Information note S3).

**Figure 2:**
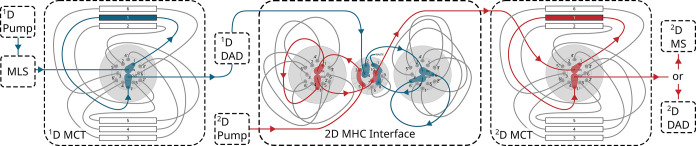
Instrumentation and flow-path scheme of the developed 2D-LC stereopeptidomics platform solution. ^1^D: Achiral column screening stack: 1, Atlantis Premier BEH C18 AX; 2, XBridge Peptide BEH C18; 3, Zorbax SB-C18; 4, Advance Bio Peptide Plus; and 5, Zorbax Bonus RP; ^2^D: Chiral column screening stack: 1, Chirobiotic T; 2, Chirobiotic TAG; 3, Crownpak CR-I­(+); 4, Crownpak CR-I(−); 5, Chiralpak ZWIX­(+); and 6, Chiralpak ZWIX(−).

### HPLC Methods

Detailed experimental conditions of the 1D-HPLC methods are summarized in Tables S1–S3. 2D-LC method parameters are described in detail in Table S4. For the multiple-heart-cutting experiments, peak-based sampling was employed and transferred to the ^2^D with ASM mode activated (ASM factor: 5.3, flush sample loop: 3×). Loops were filled to 100% (sampling time: 0.2 min). The slope mode was used as a peak trigger with an up- and down-slope of 10 mAU/s. The injection volume was 1 μL of a 100 μM concentrated sample for all methods.

## Results and Discussion

### Epifadin-Derived Tetrapeptide Amide

The current multicolumn 2D-LC stereopeptidomics platform was developed to elucidate the stereochemistry of epifadin. Epifadin (Figure S1) is a broad-spectrum and short-lived microbial peptide-polyene-tetramate, which is produced by nasal *Staphylococcus epidermidis* IVK83.^35^ Chemically, it has a unique molecular architecture consisting of a nonribosomally synthesized tetrapeptide, a polyketide component, and a terminal modified AA moiety (Asp). Isolated from the human nose with its specific microbial habitat, it showed antibacterial activity against *Staphylococcus aureus*. Unfortunately, it is highly unstable and decomposes within a few hours into the tetrapeptide amide moiety and a degraded lipid portion. The remaining tetrapeptide amide (Figure S2, H_2_N-Phe-Phe-Asp-Asn–NH_2_) was the sample analyzed in this study. Since nonribosomal peptides are known to have d-AAs incorporated, a stereoselective analysis is required to confirm their structure. Although it is a relatively simple peptide, the common approach of experimentally elucidating the stereochemistry by eAAA after full hydrolysis with D_2_O/DCl fails. Asn gets hydrolyzed to Asp so that the two distinct residues cannot be distinguished anymore. There are two Phe residues in the tetrapeptide, which may have opposite configurations; yet after hydrolysis, the sequence information (i.e., the position of each AA and hence where there might be a d- and l-form) is lost, and the same is true for Asp/Asn. On the other hand, performing the stereoselective analysis on the intact tetrapeptide level conserves the sequence information, yet leads to a relatively complex mixture of 2^*n*^-stereoisomers (with *n* being the number of stereogenic centers), i.e., sixteen possible stereoisomers in the present case (see Table S1). Considering that chiral separations typically deal with the resolution of two peaks, this is already a quite complex mixture. For an unequivocal structural assignment, all 16 individual tetrapeptide amide stereoisomers were synthesized by solid-phase peptide synthesis; it required the separation of both diastereomers and enantiomers for full resolution of this mixture.

### Chiral Separation of Epifadin-Derived Tetrapeptide Amide Stereoisomers—A Need for 2D-LC

While diastereomers of the 16 stereoisomers mixture of the epifadin-derived tetrapeptide H_2_N-Phe-Phe–Asp-Asn–NH_2_ could in principle be separated with achiral methods like RPLC or IMS, for peptide enantiomer separation, enantioselective LC with chiral stationary phases is the first choice. Generally, the scientific literature reveals primarily three distinct chiral selector systems, which have proven successful for (free, underivatized) peptide enantiomer separations, including diastereomer separations, mostly demonstrated with model peptides. These three classes of chiral selectors comprise: (i) macrocyclic glycopeptide antibiotics-based CSPs (teicoplanin T,^36^ teicoplanin aglycone TAG^27^) bind peptides preferentially through their C-terminal end by triple hydrogen bond-driven inclusion complexation with the peptide backbone.^37^ (ii) CSPs based on chiral crown ethers (Crownpak CR-I­(+) and CR-I(−)) interact at the *N*-terminus of peptides by inclusion complexation of their ammonium moiety into the 18-crown-6 macrocyclic ring, driven by triple hydrogen bonding with ether groups.^38^ (iii) Zwitterionic quinine and quinidine carbamate-based^36^ CSPs (ZWIX­(+) and ZWIX(−)), on the other hand, have the capability to bind peptides on both the *N*- and *C*-terminus through double ion-pairing as primary interactions.^39^ The mixture of all 16 H_2_N-Phe-Phe-Asp-Asn-NH_2_ stereoisomers has then been injected on these 6 chiral columns (Chirobiotic T and TAG, Crownpak CR-I­(+) and CR-I(−), and Chiralpak ZWIX­(+) and ZWIX(−)) by common one-dimensional LC (1D-LC). All 6 columns achieved a certain degree of stereoselectivity, but none of the tested columns was able to separate all 16 stereoisomers (Figure 3, top panes). However, when mixtures of the 8 enantiomer pairs were injected individually, all CSPs exhibited at least some enantiomer separations. T and TAG CSPs together could separate 6 out of the 8 enantiomer pairs (Figure 3a,b), ZWIX(−) 3 out of 8, and CR-I­(+) and CR-I(−), as well as ZWIX­(+) could separate all tetrapeptide enantiomer mixtures. Elution orders are reversed on CSPs CR-I­(+) and CR-I(−), having immobilized fully synthetic enantiomeric crown ether selectors. Strikingly, reversed elution orders are also observed on ZWIX­(+) and ZWIX(−) for those enantiomer pairs that were resolved on ZWIX(−). In conclusion, these chiral stationary phases showed remarkable enantioselectivity but insufficient chemoselectivity (diastereoselectivity) and/or peak capacity; hence, the full mixture cannot be resolved by 1D-LC only. For this reason, a 2D-LC approach was envisioned as a solution.

**Figure 3:**
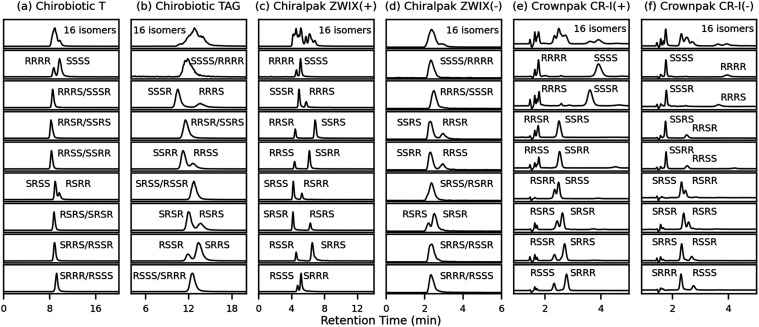
LC separations of epifadin-derived tetrapeptide amide stereoisomers on 3 types of chiral stationary phases: (a) Teicoplain (T), (b) Teicoplanin aglycone (TAG), (c) zwitterionic quinine carbamate (ZWIX­(+)), (d) zwitterionic quinidine carbamate (ZWIX(−)), (e) (*S*)-3,3′-diphenyl binaphthyl-2,2′diyl-18-crown-6, and (f) corresponding (*R*)-enantiomer. More information: Tables S2 and S8, Figures S4–S7. Note, mixtures of 1:2 or 1:3 molar ratios of enantiomers were injected to allow assignment of elution orders.

### Development of the Achiral ^1^D of the Stereopeptidomics 2D-LC Platform

Achiral column selection for the ^1^D diastereomer separation of the stereopeptidomics screening platform was guided by the work of Petersson et al.^32^ First, a set of 6 achiral RP columns were selected from the PCA score plot of 43 screened columns for peptide separations, which were distant from each other, hence presumably orthogonal, and can be classified into three groups (neutral, negative, and positive surface).^32^ Zorbax SB-C18 was selected as a standard C18 column, Acquity BEH C18 as an ethylene-bridged organosilane hybrid silica material with less silanol activity, Advance Bio Peptide Plus as a chemically charged C18 hybrid surface with slight positive surface charge, Premier Atlantis BEH C18 AX with mixed-mode RP/anion exchange surface chemistry, and Zorbax Bonus RP with densely reacted sterically protected diisopropyl-C14 groups covalently bonded through an embedded amide functionality. In the current method, these 5 columns were employed in the achiral ^1^D screening stack. A phenyl column, like Acquity CSH Phenyl (Phenylhexyl), could be envisioned as a sixth, to some extent, complementary column. These columns were integrated into the ^1^D MCT through a column selection valve (see Figure 2). All tentative ^1^D RP columns were screened by a generic gradient, which was, for better comparison, adjusted with respect to each column dimension. In this initial screening phase, every column was also tested under different mobile phase conditions (MeOH and ACN as organic modifiers, 0.1% TFA, FA, and AcOH as additives, as well as ammonium acetate and sodium phosphate as buffer systems) (Table S3). Significant complementarity could be obtained with distinct eluent systems and columns. Generally, with methanol, a lower chromatographic efficiency (thus broader peaks) due to the lower diffusion coefficients than with acetonitrile was achieved, as expected, independent of the RP column used. MeOH is therefore mainly considered a good option for phenyl­(hexyl) phases but may provide improved or orthogonal selectivity in special cases for other stationary phases as well. Further, retention profiles were similar for mobile phases with similar pH (i.e., all 3 acidic eluents and all 3 close to neutral eluents ca. pH 6); hence, only diastereomer separation results on different columns with 0.1% TFA and 10 mM ammonium acetate (pH 5.8) are depicted (results can be found in Figures S7–S10 and Table S8).

The elution order of the isomers remained consistent throughout the screening experiments. Therefore, each peak was named according to its elution number. The screening results observed with ACN as organic modifier can be summarized as follows: (i) the separations with ammonium acetate (pH 5.8) and sodium phosphate (pH 6.0) on the C18 columns are comparable. For compatibility reasons with the second dimension, ammonium acetate is preferred, especially for ZWIX and Teicoplanin/TAG columns, which are employed in the polar organic mode. For the crown ether columns, operated with highly aqueous mobile phases, both buffers are adequate from a compatibility viewpoint. (ii) With C18 columns (Zorbax and Acquity BEH) at low pH (0.1% TFA), diastereomers 5 and 6 represented the critical peak pair (Figures S7 and S9), while at high pH (close to 6; ammonium acetate and sodium phosphate), peak pairs 5 and 6 were well resolved, yet peak pairs 7 and 8 as well as 2 and 3 coeluted (on Zorbax column also partly peak 1) (see Figures S7–S10). (iii) Advance Bio Peptide could separate the critical peak pair 5 and 6 with weakly acidic AcOH eluent but was generally unfavorable otherwise, with many overlaps of diastereomers at all conditions. (iv) The mixed-mode C18 AX column showed similar retention profiles as BEH C18 with 0.1% TFA, while with less acidic conditions (0.1% FA and AcOH), peak pairs 5 and 6, but not 7 and 8, were resolved. The peak pattern changed significantly between ammonium acetate and phosphate buffers, yet multiple coelutions existed. Evidently, the separation mechanism changes on C18 AX from 0.1% TFA conditions to high pH (∼pH 6). Anion exchange plays a significant role on the C18 AX column when ammonium acetate is used, while phosphate, as a strong counterion, seems to offset this AX retention contribution so that separation is mostly based on hydrophobic interactions and profiles look similar to (BEH) C18. Furthermore, the anion exchange capacity can be controlled by the pH of the mobile phase, resulting in higher capacities with intermediate pH values (both carboxylic group and AX site ionized). When changing the modifier from acetic acid to formic acid, not only the pH value of the mobile phase but also the counterion type and concentration changes, which can result in effects acting against each other. In sum, a clear tendency for less retention is visible when changing the modifier from acetic acid to formic acid. Trifluoroacetic acid suppresses the ionization of the peptides’ carboxylic acid and forms ion pairs at the *N*-terminal amino group. TFA also causes the majority of free silanol groups of the stationary phase to be fully protonated. Hence, overall, a retention mechanism similar to that of C18 prevails. Still, the peptide analytes may experience a slight repulsive electrostatic interaction with the AX moiety of the mixed-mode C18 AX phase. This electrostatic repulsion-assisted retention mechanism^40^ reduces retention and seems to have a positive effect on the separation of peak pairs 5 and 6. Hence, based on the screening runs, BEH C18 AX was finally selected as a ^1^D separation column for further optimization of the diastereomer separation.

In the next step, fine-tuning of the diastereomer separation was carried out, in particular, focusing on optimization of the critical peak pair 5 and 6. A gain in resolution could be achieved when the temperature was decreased from 40 to 10 °C; however, no full baseline separation was obtained (Figure S11 and Table S9). Coupling two 150 mm long C18 AX columns in series with ACN-gradient containing 0.1% TFA at 15 °C as a compromise between selectivity and backpressure almost allowed the baseline separation of all diastereomers, including the critical peak pair 5 and 6 (Figure S11a). The single enantiomer pairs (see Figure S3) were injected to determine the elution order of the diastereomers on final C18 AX mixed-mode LC separation (Figure S12 and Table S6).

### 2D-LC Stereopeptidomics Platform

For the greatest flexibility in the course of 2D-LC peptide stereoisomer separations, both screenings (RPLC and enantioselective LC) were integrated into one 2D-LC screening platform. Thus, the column selection valve in the ^1^D column compartment harbored the 6 achiral columns for diastereomer separations, while the six chiral columns were attached to the column selection valve in the ^2^D column compartment. Both ^1^D and ^2^D were interfaced via a multiple-heartcutting valve with active solvent modulation capability and with two loop decks, each equipped with six 40 μL loops (Figure 1). It allows the automated collection and storage of 10 fractions from the ^1^D effluent simultaneously before analysis in the ^2^D (one loop in each deck remains free for mobile phase flow through). For a successful 2D-LC separation, the following criteria need to be considered. The separations in the two dimensions must be orthogonal to a certain degree, which is given here as the ^1^D does not separate enantiomers and the ^2^D has limited diastereoselectivity but good enantioselectivity, as discussed above. The ^1^D eluent, which represents the sample diluent of the transferred fraction into the ^2^D, must be compatible with the phase system of the ^2^D. Here, the ASM valve provides some flexibility.^41^ The practical utility of this platform is demonstrated by two real applications from natural peptide drug discovery using two distinct 2D-LC modalities.

### Multiple-Heart Cutting RP-Chiral 2D-LC-MS/MS of Epifadin-Derived Tetrapeptide Amide

The tandem C18 AX mixed-mode column with 0.05% TFA (TFA is a strong counterion on the WAX site of ZWIX­(+), and hence a lower concentration has a better compatibility with the ^2^D) was selected for the ^1^D separation. All 6 ^2^D chiral columns were screened using a 16-stereoisomer tetrapeptide standard mixture with a multiple-heart-cutting setup (for detailed parameters, see Table S5 and Figures S13–S18). In contrast to full comprehensive LC × LC 2D separations, the ^2^D speed is less critical, and no compromise in terms of resolution for speed is required. Generally, it can be performed by two modalities of sampling: time-based and peak-based. Time-based sampling requires knowledge of the retention times in the ^1^D RPLC separation, which can be obtained from a prior run. High retention time repeatability is a prerequisite in order to transfer the heart-cut at the highest peak intensity. It can be regarded as a kind of targeted approach. On the contrary, peak-based sampling (using peak properties as a trigger for sampling, such as the slope of the peak) does not require a prior run, thus can be employed as an untargeted analysis technology, and is less susceptible to slight retention shifts.When using a different MS vendor (Sciex) than the 2D-LC system (Agilent), the MS run is started by the signal transferred from the Agilent multisampler to the Sciex MS over the ERI cable and stopped by the fixed LC and MS run times. A prior run is needed for determining the number of cuts and thus the run-time in peak-based sampling approach with non-Agilent MS detector. Since the number of cut peaks (and hence ^2^D runs) were known, peak-based sampling with a run-time corresponding to eight heart cuts was employed for fully automated 2D-LC for all tetrapeptide amide samples. As expected from the preoptimization runs, 2D-LC chromatograms with Chirobiotic T (Figure S17) and TAG (Figure S18) were not useful because only a limited number of stereoisomers could be resolved. Here, it must be pointed out that newer Teicoplanin and TAG columns are available on the market, which might give better separations, and further mobile phase optimization may also lead to better success. The employed ^1^D aqueous mobile phase with 0.05% TFA eluents in combination with ASM was well compatible with the polar ionic elution mode (polar organic solvents containing ammonium formate) of the ^2^D. Crownpak CR-I­(+) and CR-I(−), on the other hand, are well compatible with the ^1^D. These chiral crown ether CSPs require strongly acidic eluents (typically high concentrations of TFA or perchloric acid) to create positively charged ammonium ions for inclusion complexation in the crown ether macrocycle. Herein, we used 0.2% TFA as an additive to water/MeOH/ACN mixtures as a ^2^D mobile phase; hence, the 0.05% TFA from the ^1^D is uncritical. However, high ACN contents from the ^1^D might cause peak shape problems; hence, ASM was activated. As can be seen from Figure S15 (Crownpak CR-I­(+)) and S16 (Crownpak CR-I(−)), all 16 stereoisomers were fully baseline separated by this 2D-LC approach. It is striking that enantioselectivity declines with increasing lipophilicity of the diastereomers, i.e., enantiomers of early eluted, less lipophilic diastereomers in ^1^D show larger separation factors in the ^2^D than later eluted ones. Due to the availability of enantiomeric fully synthetic selectors, enantiomer elution orders can be reversed on Crownpak CR-I­(+) and CR-I(−) (cf. Figure S15 and S16). Although ^2^D elution conditions are generally MS compatible, we disfavored this 2D-LC system for the sake of high 0.2% TFA in the ^2^D, which may lead to ion suppression in ESI-MS detection of peptides.

For the final determination of the tetrapeptide stereoisomer configuration, the combination of tandem C18 AX in ^1^D and the ZWIX­(+) column in ^2^D was used. Because TFA is a strong counterion on the AX moiety and hence a strong eluent on the ZWIX­(+), its concentration in the ^1^D was reduced to 0.05%, and ASM was activated, allowing the loop volume to be diluted with the weak ^2^D eluent (channel ^2^A). This ^2^D weak eluent (Table S5, method Nr. 34) has a low concentration of counterions only (2.5 mM NH_3_, 5 mM FA) competing for the ion-exchange sites, and a high concentration of acetonitrile (i.e., largely nonprotic polar organic mode), which both resulted in weak elution strength. Due to the high salt concentration of the other ^2^D eluent (channel ^2^B; 25 mM NH_3_, 50 mM FA) and the resulting high UV cutoff concentration, UV detection was unfavorable. However, these ^2^D elution conditions are well compatible with ESI-QTOF-MS/MS detection, which is the preferred detection mode in peptidomics research. Figure 4 shows the successful 2D-LC separation. The bottom panel in each subfigure represents the ^1^D UV chromatogram and the upper panel the 2D MS2 contour plot (with a zoom of the peak pairs 5 and 6) reconstructed from the EIC of *m*/*z* 410.171 corresponding to the b_3_ fragment ion from the precursor isolation (SWATH) window *m*/*z* 539.5–560.5. In the ^1^D UV chromatograms, the sampling times of the heart cuts are indicated in light green. The cuts were triggered by the ^1^D UV signal (peak-based, slope). Thus, the results were not affected by small retention time shifts. All 16 stereoisomers were separated in the 2D chromatogram. In the zoom in, it can be seen that the heart cut of peak number five also contains some traces of peak number 6. Fortunately, the ZWIX­(+) column provides (besides enantioselectivity) also diastereoselectivity, so that those peaks can also be separated in the ^2^D. The enantiomer peak assignment was made on the basis of the 1D separation, shown in Figure S4, and the diastereomer peak assignment based on Figure S12. Figure S19 shows the 2D contour plot of a real sample of tetrapeptide amide produced by nasal *S. epidermidis* IVK83. From the 2D contour plot of this sample, the configuration of *SRSR* was determined for the microbial natural tetrapeptide amide sample (Figure S19a) and confirmed by injection of the corresponding single stereoisomer standard (Figure S19b).

**Figure 4:**
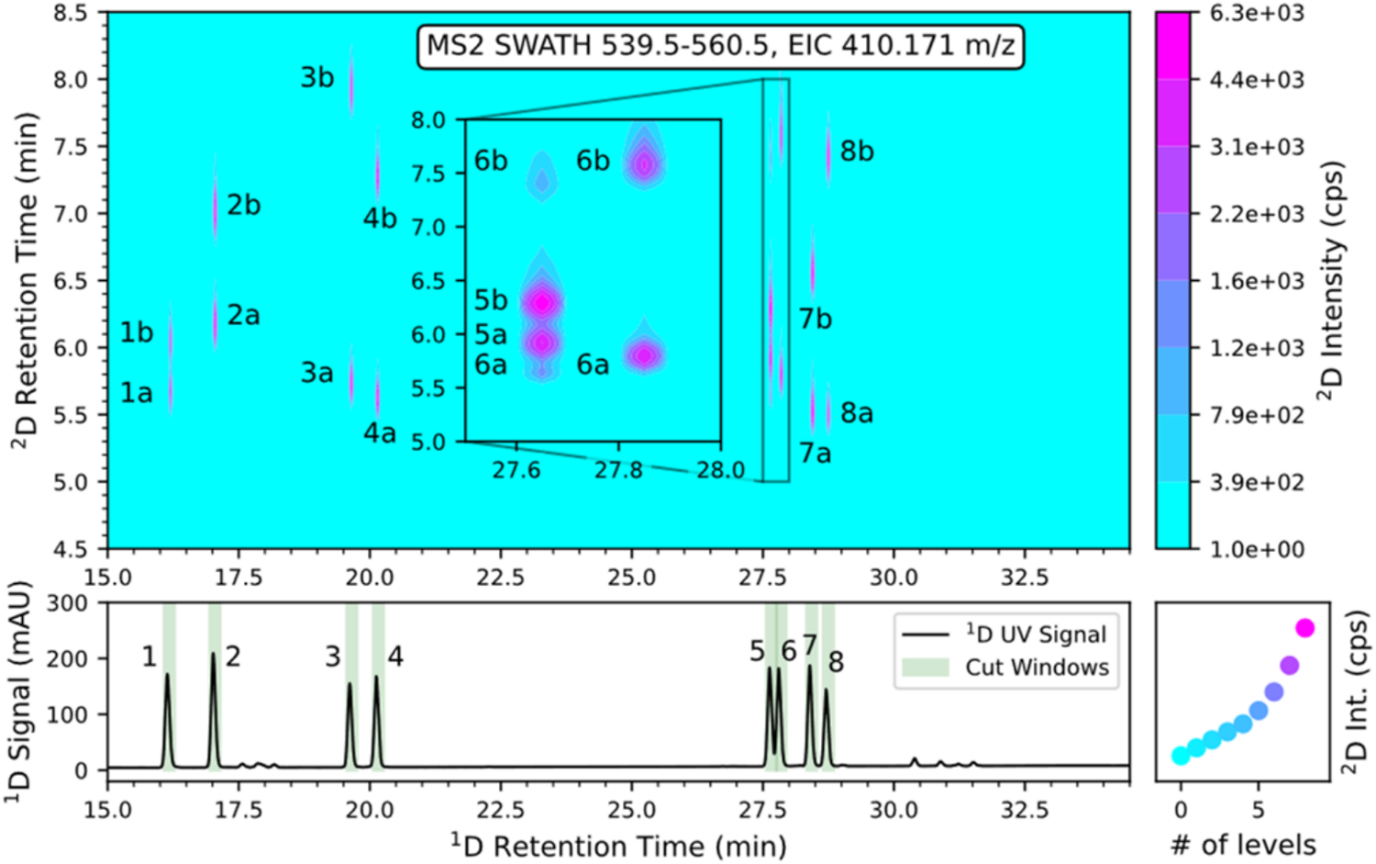
Stereoselective multiple-heart-cutting 2D-LC-MS/MS analysis (with SWATH acquisition) of tetrapeptide (Phe-Phe–Asp-Asn–NH_2_) 16 standards using C18 AX in ^1^D and ZWIX­(+) in ^2^D. For peak annotation, see Table S6. Conditions: see Table S5, method 34. b_3_ fragment ion (*m*/*z* 410.171) of the MS2 level was used for generation of the EIC 2D-plot. Peak annotation: 1a, *RRRR*; 1b, *SSSS*; 2a, *SSSR*; 2b, *RRRS*; 3a, *RRS*R; 3b, *SSRS*; 4a, *RRSS*; 4b, *SSRR*; 5a, *RSSS*; 5b, *SRRR*; 6a, *RSSR*; 6b, *SRRS*; 7a, *SRSS*; 7b, *RSR*R; 8a, *SRSR*; and 8b, *RSRS*. Zoom shows an enlarged view of the respective chromatographic window.

### Degradation Analysis of Epifadin-Derived Tetrapeptide

As can be seen in the ^1^UV chromatogram of Figure 4, the tetrapeptide standards show minor degradation peaks in the elution window between 17.5 and 18.5 min as well as between 30 and 32 min. The H_2_N-Phe-Phe-Asp-Asn-NH_2_ tetrapeptide can undergo multiple spontaneous degradation or isomerization pathways due to its Asp and Asn contents. In Figure S23, favorable degradation pathways are described in more detail for the *SSSS* isomer as an example. These pathways are the same for all 16 isomers. Generally, they can be classified into (i) deamidation products [M-NH_3_+H_2_O+H]^+^ (with mass shift of Δ*m*/*z* + 1 and altered carboxylic acid number ΔCOOH of +1) (double deamidation also possible), (ii) Asp-succinimides (Asu peptides) [M-H_2_O+H]^+^ (with Δ*m*/*z* −18 and ΔCOOH −1), (iii) epimers ([2M+H]^+^/[M + H]^+^; originating from Asp-succinimide formation followed by epimerization via enolization and subsequent hydrolysis, resulting in Δ*m*/*z* 0 and ΔCOOH 0), (iv) isoAsp isomers (through Asp/isoAsp isomerization via corresponding Asu peptides), and (v) deamidated isoAsp peptides (with Δ*m*/*z* + 1 and ΔCOOH + 1). Figures S20–S22 show 1D-LC/MS measurements with the Tandem BEH C18 AX column and the ZWIX­(+) column. The 1:3 *RRRR*/*SSSS* standard mixture (sample from peak assignments) was used as an example to document the selectivity of the two columns for these degradation products (note, the corresponding generated impurities of *RRRR* and *SSSS* tetrapeptide are enantiomeric and hence not separated on achiral LC). Multiple impurity/degradation peaks were detected when using the C18 AX column, demonstrating the great utility of the mixed-mode C18 AX column for impurity profiling of peptides with altered charge state. The TOF-MS EIC with *m*/*z* 541.241 ([M + H]^+^) (Figure S21a and S22b) shows one major and multiple minor degradation peaks besides the target tetrapeptide. Since epimerization via Asp-succinimide and subsequent hydrolysis leads to *SSRS*/*RRSR* diastereomer (3), this peak could be assigned by the standard. The other minor degradation peaks could possibly be isoAsp isomer peaks (not verified due to the absence of standards). The EIC trace for deamidation, EIC [M-NH_3_+H_2_O+H]^+^ with *m*/*z* 542.228 (Figure S21b), shows two major degradation peaks (and a few minor ones) that are more strongly retained on the C18 AX as expected due to an additional carboxylic acid group. In contrast, no significant double deamidation peaks were found (EICs not shown). On the other hand, the EIC for [M-H_2_O+H]^+^ with *m*/*z* 523.230 corresponding to the Asp-succinimide peptide showed two peaks (Figure S21c), indicating that epimerization via the enol form of the Asu-peptide took place. Due to the presence of significant signals for this degradation product, the formation of epimers *SSRS*/*RRSR* and isoAsp isomers is strongly supported (see Figures S22 and S24–S26). 1D-LC analysis by the chiral ZWIX­(+) phase did not allow deciphering of the entire impurity profile (Figure S20). eAAA of the fully hydrolyzed peptide sample does not provide information on Asp/isoAsp and other impurities; the info gets lost during hydrolysis. It demonstrates that the current 2D-LC-MS platform has extended capability to deal with such challenging analytical problems in peptide quality control. A more thorough discussion of this topic is beyond the goal of the current study.

### Selective Comprehensive RP × Chiral 2D-LC-MS/MS of Lipopeptide-Derived Dipeptide

The trideca-modular NRPS-derived lipopeptide with a partial sequence of *N*-3-hydroxyfatty acyl-Glu-Glu–Leu was analyzed and used as an example for ePBBA. The partial structure is shown in Figure S27. The eAAA of the 13 AA-constituted lipopeptide provided the configurations of all AAs. Glu was present twice, once in the l and once in the d-configuration. This raised the question of at which position in the peptide chain d-Glu is located. Partial hydrolysis (as described in detail in the sample preparation part of the Supporting Information) was performed, digesting the peptide randomly into di-, tri-, or tetrapeptides (ePBBA). Herein, only dipeptides were used as a source of information. While the complete sequence information was lost (but already available from native peptide analysis), the information about the absolute configurations of the dipeptides, including their partial sequence, remained available in these peptide building blocks. In the case of the current lipopeptide, the open question of the configurations of the two Glu residues could be addressed by three possibilities: the fatty acid-Glu-, Glu–Glu-, or Glu–Leu-dipeptide. While Glu–Glu dipeptide could also be well resolved into 4 stereoisomer peaks (see Figure S28), Glu–Leu could be readily detected in the partial digest and was selected to determine which Glu has d configuration (this partial sequence existed only once in the peptide and the configuration of Leu was determined as d).

The same column combination in the ^1^D (C18 AX) and ^2^D (ZWIX­(+)), and the same mobile phases (in A and B channels) were used as above in the context of Figure 4. Instead of multiple-heart-cutting triggered by the UV signal, a selective comprehensive sLC × LC mode of 2D-LC was used in this case for the following reason. No peak in the ^1^D UV signal could be detected for the digested real sample due to the limited sample quantity available and low concentration of the targeted Glu–Leu dipeptide after partial digestion. Each diastereomer peak of Glu–Leu was sampled comprehensively by four fractions (marked in green in Figure 5, bottom ^1^D UV chromatograms). The resulting 2D plots show peaks for each transferred Glu–Leu cut from the ^1^D (note, distinct cuts across the same ^1^D peak were not merged). Figure 5a depicts the sLC × LC 2D chromatogram of a mixture of the four synthesized Glu–Leu dipeptide standards. As can be anticipated from Figure 5a, 1D-LC on Chiralpak ZWIX­(+) would not be capable of separating all four stereoisomers under the given ^2^D LC conditions. Two diastereomers would always overlap in 1D-LC on ZWIX­(+). The mixed-mode C18 AX, on the other hand, has excellent diastereoselectivity. By combination of the two dimensions, all 4 stereoisomers can be readily resolved (note that the d-Glu-d-Leu standard at 9 (^1^D) and 4 min (^2^D) was lower concentrated in the mix; hence, only cut#2 shows a peak). The peak assignment was made with individual synthetic standards of the stereoisomers (see Figure S29 and Figure S30). Figure 5b, bottom, reveals the ^1^D UV trace of the partially hydrolyzed lipopeptide sample. It is evident that the concentration of the target dipeptide is below LOD, and peak-based sampling with slope-mode as peak trigger of sampling would fail. The problem can be readily circumvented by a selective comprehensive 2D-LC mode. Figure 5b, top, depicts the 2D chromatogram of the partially hydrolyzed lipopeptide sample. There is a major peak visible at the retention times of the d-Glu-d-Leu stereoisomer, which allows unequivocal assignment of the absolute configurations of the Glu-Leu dipeptide, which occurs only once in the lipopeptide and allows identification of its stereochemistry within the sequence. While this specific example could have been solved by 1D-LC with two diastereomer standards (as the configuration of Leu was known), it documents the general applicability of the concept and illustrates how to deal with larger peptides, of which too large a number of stereoisomers exist.

**Figure 5:**
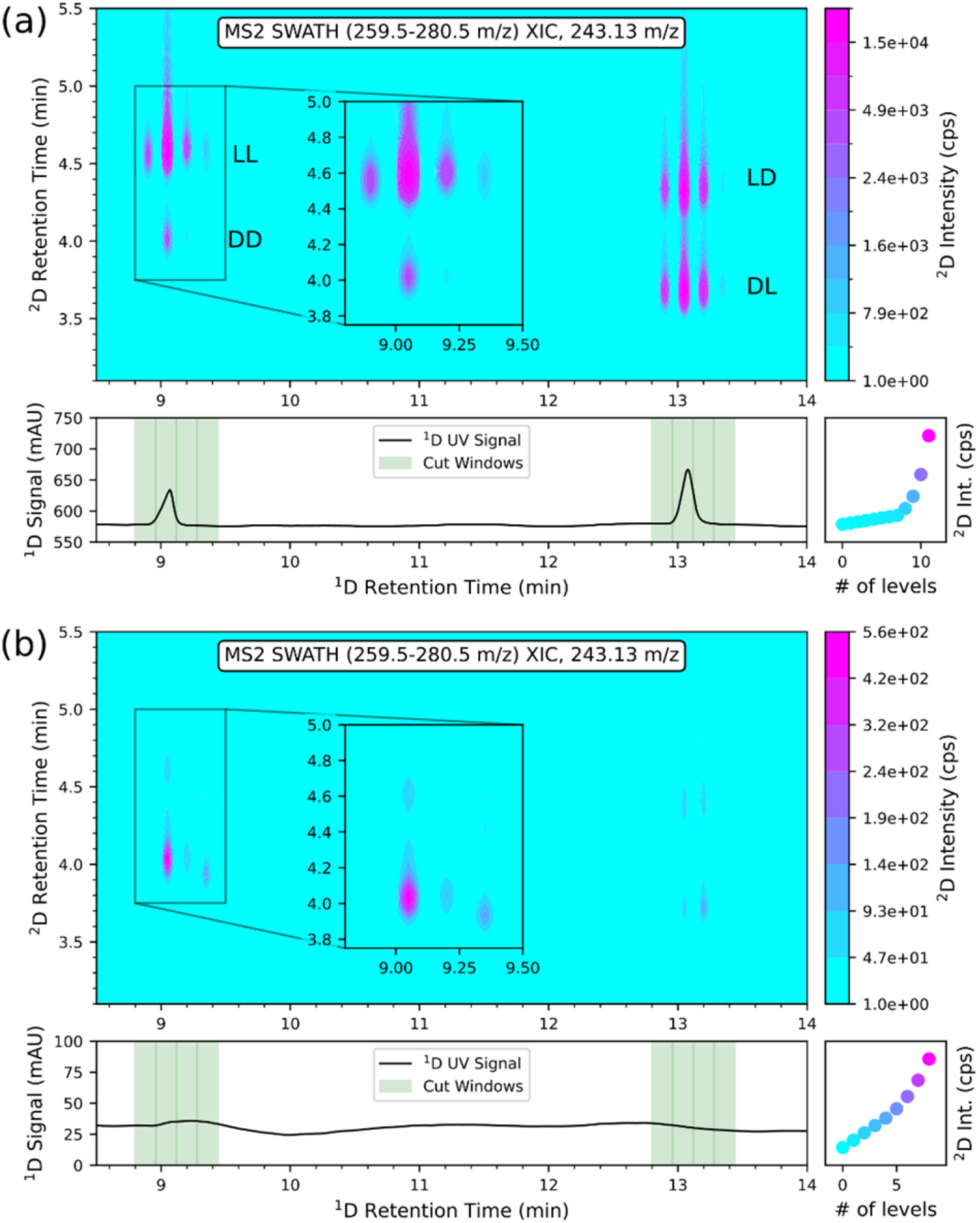
ePBBA of Glu-Leu by selective comprehensive 2D-LC-MS/MS analysis. (a) Mixture of synthetic standards of all 4 stereoisomers (Figures S29 and S30), and (b) Glu-Leu sample from partially digested lipopeptide (isolated natural product; peaks at ^1^ *t*_R_ = 9 min and ^2^ *t*_R_ = 4 min indicate DD configuration of dipeptide in natural product). Conditions: see Table S4, method 25. Zoom presents an enlarged view of the respective chromatographic window.

## Conclusions

The growing importance of synthetic and biotechnologically produced peptide therapeutics raises the demand for analytical methods that can deal with the selective analysis of peptide isomers, including stereoisomers. Also, the most scarce amounts of ecologically indispensable peptides in studies of interspecies communication and interaction need reliable stereoselective analysis. Distinct chromatographic platforms with solid methods fuel the field of chemical ecology. Full hydrolysis and subsequent enantioselective amino acid analysis has long been a method of first choice to test for the presence of d-amino acids and hence indirectly of d-amino acid-containing peptides. The methodology has some shortcomings in pinpointing the position of detected d-amino acids within the peptide chain if a specific amino acid is present more than once in L and D configuration. Generally, in this study, it turned out that chiral columns have good enantiomer selectivity of peptides but have problems to separate complex mixtures of multiple stereoisomers. On the contrary, RP-type columns showed good diastereoselectivity but cannot separate enantiomers. With an online coupled system of achiral mixed-mode C18 AX in the ^1^D and chiral column (herein ZWIX­(+)) in the ^2^D, more complex peptide stereoisomer mixtures, such as a 16-stereoisomer mixture of a tetrapeptide, could be fully resolved. The configuration, including sequence information, can be obtained. In combination with partial hydrolysis to small oligopeptides, the current stereopeptidomics platform can also be applied to more complex peptides (ePBBA). Synthetic peptide therapeutics can be partially digested, and their peptide stereoisomers can be analyzed by this platform, in a similar manner to the present lipopeptide. In sum, a systematic approach is presented to combine information derived from eAAA, ePBBA, and native analysis to fully analyze and determine full stereochemical information on peptides. As part of this approach, the use of 2D-LC analysis in stereopeptidomics has proven to be essential for in-depth degradation analysis and for pushing the boundaries of conventional 1D-LC.

## Supplementary Material



## Notes

Supporting information notes on standard synthesis, materials and columns, and Jupiter notebook; Supporting information tables with mobile phases and experimental conditions, and chromatographic parameters; Supporting information figures with 1D-LC chromatograms of the ^1^D and ^2^D optimization, 2D plots of C18 AX with Chirobiotics T&TAG, Crownpak CR-I­(+)&(−), and Chiralpak ZXIX(−), and 1D and 2D-LC peak assignment of dipeptides (PDF)
