# Supplementary material for: Multicolumn Two-Dimensional Liquid Chromatography Screening Platform for Stereopeptidomics and Application to Antimicrobial Peptide Polyene and Lipopeptide
Source: Anal Chem. 2025 Jun 24;97(26):14048–57. doi: 10.1021/acs.analchem.5c02658 (PMC13010258; doi:10.1021/acs.analchem.5c02658)
Supplement: Supplementary file 1 [file ac5c02658_si_001.pdf]

Supporting Information

Multi-column two-dimensional liquid chromatography screening platform for stereopeptidomics and application to antimicrobial peptide polyene and lipopeptide

Cornelius Knappe [a], Simon J. Jaag [a], Taulant Dema [b], Ruslan Jaufmann [a], Stephan Buckenmaier [c], Harald Gross [d], Stephanie Grond [b], Michael Lämmerhofer [a, *]

[a] Pharmaceutical (Bio-)Analysis, Institute of Pharmaceutical Sciences, University of Tübingen, Auf der Morgenstelle 8, 72076 Tübingen, Germany

[b] Institute of Organic Chemistry, University of Tübingen, Auf der Morgenstelle 18, 72076 Tübingen, Germany

[c] R&D Liquid Phase Division, Agilent Technologies Deutschland GmbH, Hewlett-Packard-Str. 8, 76337 Waldbronn, Germany

[d] Pharmaceutical Biology, Institute of Pharmaceutical Sciences, University of Tübingen, Auf der Morgenstelle 8, 72076 Tübingen, Germany

*Authors for correspondence:

Prof. Dr. Michael Lämmerhofer
Pharmaceutical (Bio-)Analysis
Institute of Pharmaceutical Sciences
University of Tuebingen
Auf der Morgenstelle 8
72076 Tuebingen, Germany
T +49 7071 29 78793, F +49 7071 29 4565
E-Mail: michael.laemmerhofer@uni-tuebingen.de

Table of Contents

Supplementary Note S1. Sample preparation 4
Supplementary Note S2. Materials 5
Supplementary Note S3. Jupyter Notebook. 7

Table S1.Labels of the tetrapeptide of epifadin. 17
Table S2. 1D chiral HPLC methods. 18
Table S3. 1D achiral HPLC screening methods. 18
Table S4. 1D temperature screening methods. 20
Table S5. 2D HPLC methods (final methods). 21
Table S6. Summarized elution pattern of all chiral columns. 22
Table S7. Summarized separation parameters of 1D chiral columns. 24
Table S8. Separation parameters of 1D column screening. 27
Table S9. Separation parameters of temperature screening and tandem column. 32

Figure S1. Structure of epifadin. 34
Figure S2. Structure of the tetrapeptide of epifadin. 34
Figure S3. Fragment ion analysis for tetrapeptide of epifadin. 34
Figure S4. 1D chiral separations with zwix columns. 35
Figure S5. 1D chiral separations with Crownether columns. 36
Figure S6. 1D chiral separations with CHIROBIOTIC columns. 37
Figure S7. 1D achiral separations with Waters XBridge Peptide BEH C18 38
Figure S8. 1D achiral separations with the Waters Atlantis Premier BEH C18 AX. 39
Figure S9. 1D achiral separations with Agilent Zorbax SB-C18. 40
Figure S10. 1D achiral separations with the Agilent AdvanceBio Peptide Plus 41
Table S11. Comparison between Single vs Tandem Column and Temperature 42
Figure S12. 1D achiral summary. 43
Figure S13. 2D plot with BEH C18AX and ZWIX (-). 44
Figure S14. 2D plot with BEH C18AX and ZWIX (+). 44
Figure S15. 2D plot with BEH C18AX and CR-I (+). 45
Figure S16. 2D plot with BEH C18AX and CR-I (-). 45
Figure S17. 2D plot with BEH C18AX and Chirobiotic T. 46
Figure S18. 2D plot with BEH C18AX and Chirobiotic TAG. 47
Figure S19. 2D plot comparison of sample and SRSR standard 47
Figure S20. 1D LC-MS degradation analysis with ZWIX (+). 48
Figure S21. 1D LC-MS degradation analysis with tandem C18 AX of *SSSS*/*RRRR* standard mix. 48
Figure S22. 1D LC-MS degradation overview of analysis with BEH C18 AX. 49
Figure S23. Possible degradation or isomerization pathways of the tetrapeptide. 50

Figure S24. 1D LC-MS degradation analysis with tandem BEH C18 AX, MS1 541 *m/z*. 51
Figure S25. 1D LC-MS degradation analysis with tandem BEH C18 AX, MS1 542 *m/z*. 52
Figure S26. 1D LC-MS degradation analysis with tandem BEH C18 AX, MS1 523 *m/z*. 53
Figure S27. Partial structure and building blocks of lipopeptide. 54
Figure S28. Separation of Glu-Glu. 54
Figure S29. 1D-LC separation of Glu-Leu standards. 55
Figure S30. 2D plot of Glu-Leu. 56

Supplementary Note S1. Sample preparation

Synthesis of Epifadin-derived tetrapeptide standards

The tetrapeptide (NH_2-Phe-Phe-Asp-Asn-NH_2) stereoisomers were synthesized by stereoselective solid-phase peptide synthesis (SPPS). The reaction and washing steps were carried out in a peptide synthesis reactor (5 mL, 25 µm polypropylene frit, Carl Roth). The SRSR tetrapeptide was synthesized starting with swelling of the Fmoc-R-Asn(Trt) resin (TG S RAM, Rapp Polymere, loading: 0.23 mmol/g, 150 mg, 34.5 µmol scale) in 2 mL DMF for 30 min. First the Fmoc protection group was cleaved by alkalic treatment with a solution of 2 % DBU/10% morpholine in DMF (v/v) for three min and an additional second treatment for 12 min. The resin-bound residue was then submitted to iterative peptide assembly using a solution of the respective Fmoc-R/S-AAx-OH (Fmoc-S-Asp(OtBu)-OH, Fmoc-R-Phe-OH, Fmoc-S-Phe-OH) (6 equivalents), HOBt and HATU (both 6 equiv.) and NMM (8 equiv.) in 2 mL DMF to couple the next amino acid in a 45 min reaction. Subsequently the Fmoc-deprotection was performed using 2 % DBU/10% morpholine in DMF (v/v) for three min and an additional second treatment for 12 min. After full assembly and final Fmoc-deprotection of the tetrapeptide amide on the solid support, the resin was washed with 3x 2 mL DMF, 3x 2 mL DCM, 3x 2 mL toluene, 3x 2 mL IPA, 3x2 mL diethylether (Et2O) and then dried under reduced pressure for three hours. The dried peptide was cleaved from the resin by acidic treatment with TFA/TIPS/H2O (95:2.5:2.5 v/v, 2 mL) for one hour and for a total of three times. A final cleavage step with 2 mL pure TFA for 10 min was performed. The cleavage solvents were removed under reduced pressure and the residue washed three times with 2 mL Et2O. The precipitate was dissolved in 10 mL tert-butanol/H2O (1:1 v/v) and lyophilised. All other stereoisomers were synthesized in the same fashion using the respective Fmoc-R/S-Asn(Trt) resins and Fmoc-R/S-AAx-OH amino acids.

Dipeptide synthesis (NH_2-Glu-Leu-OH)

Leucine(OtBu): Place tert-butanol (43.2 eq) and cool to 0 °C. Add acetyl chloride (13.2 eq) slowly under constant stirring, ensuring the temperature remains below 5 °C. Cool the mixture to 0 °C, and add leucine (1.0 eq). After 2 hours, add concentrated HClO4 (70%, 3.0 eq) dropwise. Stir the reaction mixture at rt for 12 h. Extract the solution with water (3 x 25 mL). Add 5% HCl (2 x 25 mL) to the organic phase and combine the aqueous phases. Add K2CO3 to the aqueous phases until no gas production is visible. Extract with ethyl ether (2 x 25 mL). Combine the organic phases, dry over MgSO4, and remove the solvent under vacuum. The resultant product was obtained as a slightly yellowish oil.

Fmoc-Glu(OtBu)-NHS: Place dichloromethane (DCM, 10 mL) and cool to 10 °C. Add Fmoc-Glu(OtBu)-OH (1.0 eq) with dicyclohexylcarbodiimide (DCC, 1.3 eq) and N-hydroxysuccinimide (NHS, 1.4 eq). Stir the mixture for 2 h at rt. Filter the Urea using Celite. Remove the solvent under vacuum, yielding a slightly yellowish product.

Fmoc-Glu(OtBu)-Leu(OtBu): Dissolve Fmoc-Glu(OtBu)-NHS (1.0 eq) in acetone. Add leucine(OtBu) (1.2 eq) and $NaHCO_3$ (4.1 eq) to the reaction mixture at rt. Stir for 30 min, then remove the acetone. Extract with diethyl ether (3 x 2 mL). Dilute the aqueous phase with water (2 mL). Cool the reaction mixture to 0 °C and adjust the pH to between 2-3 using HCl (1M). Extract the aqueous phase with diethyl ether (3 x 2 mL). Wash the organic phases with brine and water, combine, and dry over MgSO4. Remove the solvent under vacuum. Obtain a slightly yellowish oil as product.

Fmoc-Glu-Leu: Add (v/v) piperidine (20%, 12.0 eq) and DMF (1 mL) to the Fmoc-Glu(OtBu)-Leu(OtBu) (1.0 eq). Stir the reaction mixture at room temperature for 5 min. Filter the reaction solution and wash with DMF (1 mL). Add the same amount of piperidine again and stir the reaction mixture at rt for another 10 min. Filter the mixture and wash with DMF (2 x 1 mL). Remove the solvent under vacuum.

Glu-Leu: Dissolve Fmoc-Glu-Leu in DCM (10 mL) and add TFA (6 mL). Stir the mixture at rt for 3h. Remove the solvent under vacuum, yielding a slightly yellow product. The product was further purified using analytical preparative LC-Method.

Preparation of the hydrolysate of the lipopeptide (partial hydrolysis)

The lipopeptide was hydrolysed in 1.5 mL PCR clean safe-lock tube (Eppendorf, Hamburg, Germany) by dissolving 1 mg of the substance in 100 µL of ACN and 900 µL of 6 M deuterated hydrochloric acid (DCl/D_2O). Then, the glass vial was flushed with argon, sealed, and heated to 100°C on a thermo shaker (Eppendorf, Hamburg, Germany). After 30 minutes, one hour, and two hours, 300 µL were taken out from the vial each time and finally pooled again after the end of the reaction. After reaction, the partial hydrolysate solution was subsequently evaporated to dryness on a Genevac EZ-2 high performance evaporator (SP Scientific, Ipswich, UK). The sample was reconstituted in the initial mobile phase without modifiers or salt.

Supplementary Note S2. Materials

The following reversed phase columns were used in the ^{1}D: Atlantis Premier BEH C18 AX (150 x 2.1 mm, 1.7 µm), Atlantis Premier BEH C18 AX (50 x 2.1 mm, 2.5 µm) and XBridge Peptide BEH C18 (250 x 2.1 mm, 3.5 µm) from Waters (Milford, USA); Zorbax SB-C18 (100 x 2.1 mm, 1.8 µm), AdvanceBio Peptide Plus (50 x 2.1 mm, 1.9 µm) and Zorbax Bonus RP (2.1 x 50, 1.8 µm) from Agilent Technologies (Santa Clara, USA). The ^{2}D chiral columns Chiralpak ZWIX(+) (150 x 3 mm, 3 µm), Chiralpak ZWIX(-) (150 x 4 mm, 3 µm), Crownpak CR-I(+) (150 x 3 mm, 5 µm) and Crownpak CR-I(-) (150 x 3 mm, 5 µm) were purchased from Chiral Technologies (Illkirch-Graffenstaden, France). Astec Chirobiotic T (250 x 4.6 mm, 5 µm) and

Astec Chirobiotic TAG (250 x 4.6 mm, 5 µm) were obtained from Merck (Darmstadt, Germany). Methanol and acetonitrile were purchased in Ultra LC-MS grade (Rotisolv) from Carl Roth (Karlsruhe, Germany). HPLC grade methanol and acetonitrile were obtained from Sigma-Aldrich (Steinheim, Germany). Demineralized water was purified by Elga LabWater Ultra purification system (Celle, Germany). Acetic acid, formic acid, trifluoroacetic acid and ammonium acetate each in LC-MS grade were obtained from Carl Roth (Karlsruhe, Germany). Sodium dihydrogen phosphate (anhydrous, Suprapur) was purchased from Merck (Darmstadt, Germany). All MS data was acquired in Sequential Window Acquisition of all Theoretical Mass Spectra (SWATH-MS) mode, consisting of a MS1 full scan and various SWATH experiments (MS2). For full scan an accumulation time of 50 ms, collision energy of 10 V and a scan range of 100-2000 *m/z* were used. 24 SWATH windows spanned the area of 50-1200 *m/z* for precursor selection: 50-200, 200-220.5, 220.5-240.5, 240.5-260.5, 260.5-280.5, 280.5-300.5, 300.5-320.5, 320.5-340.5, 340.5-360.5, 360.5-380.5, 380.5-400.5, 400.5-420.5, 420.5-440.5, 440.5-460.5, 460.5-480.5, 480.5-500.5, 500.5-520.5, 520.5-540.5, 540.5-560.5, 560.5-580.5, 580.5-600.5, 600-800, 800-1000, 1000-1200. The MS/MS experiments utilized an accumulation time of 100 ms and rolling collision energy with a collision energy spread (CES) of 15 V. For all MS experiments a declustering potential (DP) of 100 V, curtain gas (CUR) 30 psi, nebulizing gas (GS1) 50 psi, heater gas (GS2) 40 psi, a source temperature of 450°C and an ion spray voltage floating (ISVF) of 5500 V were used.

Supplementary Note S3. Jupyter Notebook. For data visualization and plotting of 1D-LC and 2D-LC chromatograms different open-source Python packages SciPy[1], NumPy[2] and Pandas[3] were used. Actual plots were generated with Matplotlib[4]. All written code is summarized in a Jupyter Notebook[5] and provided in the supporting information (supplementary note 3). Further details of the self-programmed python module with description and discussion of the data import functions will be published elsewhere.

1 Make Imports

[1]:
```
import numpy as np
import pandas as pd
import matplotlib as mpl
import matplotlib.pyplot as plt
import AKLaemmLib as akl
```

[2]:
```
import warnings
warnings.filterwarnings('ignore')
```

2 Show version of libraries

[3]:
```
print(f'Numpy version: {np.__version__}')
print(f'Pandas version: {pd.__version__}')
print(f'Matplotlib version: {mpl.__version__}')
print(f'Matplotlib backend: {mpl.get_backend()}')
```

```
Numpy version: 1.26.4
Pandas version: 2.2.2
Matplotlib version: 3.8.4
Matplotlib backend: module://matplotlib_inline.backend_inline
```

[4]:
```
SMALL_SIZE = 8
MEDIUM_SIZE = 10
BIGGER_SIZE = 12

plt.rc('font', size=SMALL_SIZE)          # controls default text sizes
plt.rc('axes', titlesize=SMALL_SIZE)     # fontsize of the axes title
plt.rc('axes', labelsize=MEDIUM_SIZE)    # fontsize of the x and y labels
plt.rc('xtick', labelsize=SMALL_SIZE)    # fontsize of the tick labels
plt.rc('ytick', labelsize=SMALL_SIZE)    # fontsize of the tick labels
plt.rc('legend', fontsize=SMALL_SIZE)    # legend fontsize
plt.rc('figure', titlesize=BIGGER_SIZE)  # fontsize of the figure title
```

3 Define file path's as list of list's

[5]:
```
pathlist = [
[
    # hplc metadata
    "../sequenzes_msdata/231022_grond_ax_tandem_all_ms_2d 2023-10-22 18-40-20/
 ↪002-D1F-A2-16_mix.D/PMP3.AnalyticalResults.drvml",

    # exported eic data
    "../sequenzes_msdata/231022_grond_ax_tandem_all_ms_2d 2023-10-22 18-40-20/" \
    "231022_grond_2D_ms/
 ↪231022_2D_grond_D1F-A2_16_mix_1uL_ms2_541_241_xic_410_171.txt",
],
]
```

4 Read all drvml files and summarize data in a Pandas dataframe

[6]:
```
df_drvml = akl.read_agilent_mhc_drvml_files_from_pathlist(pathlist)
```

[7]:
```
df_drvml.sort_values('From1D')
```

[7]:

	RunNumber	CutNumber	From1D	To1D	From2D	To2D	Loop	\
0	1	1	16.075408	16.275365	16.359576	27.326309	1	
1	1	2	16.946933	17.146894	60.360498	71.327245	1	
2	1	3	19.560345	19.760306	49.360294	60.327038	2	
3	1	4	20.065149	20.265110	38.360081	49.326827	3	
4	1	6	27.565380	27.759965	115.361664	126.328414	1	
5	1	7	27.759965	27.942792	104.361452	115.328199	2	
6	1	8	28.332283	28.532244	93.361242	104.327989	3	
7	1	9	28.651978	28.851941	82.361030	93.327777	4	

	Deck	Sampling Time
0	A	0.199958
1	B	0.199961
2	B	0.199961
3	B	0.199961
4	A	0.194585
5	A	0.182827
6	A	0.199961
7	A	0.199962

5 Read all ^{1}D signals into a list of Pandas dataframe's

[8]:
```
list_ch = akl.read_agilent_1d_ch_files_from_pathlist(pathlist, signal="DAD1A")
```

[9]:
```
list_ch
```

[9]:
```
[              Time    Signal
0         0.000208 -0.068009
1         0.000417 -0.066049
2         0.000625 -0.064418
3         0.000833 -0.063844
4         0.001042 -0.061929
...            ...       ...
695977  144.995417  0.094086
695978  144.995625  0.086524
695979  144.995833  0.083052
695980  144.996042  0.086389
695981  144.996250  0.092760

[695982 rows x 2 columns]]
```

6 Generate a first dimension plot

[10]:
```
labels = [r"First Run $^{1}$D UV Signal"]

%matplotlib inline
fig, ax = plt.subplots(figsize=(22.35, 8.9))

for no, measurement in enumerate(list_ch):
    ax.plot(list_ch[no]["Time"], list_ch[no]["Signal"], label=labels[no],␣
 ↪linewidth=2)

for cut in df_drvml.index:
    if cut == 0:
        rectangle = mpl.patches.Rectangle(
            (df_drvml.iloc[cut]['From1D'], 0),
            df_drvml.iloc[cut]['To1D']-df_drvml.iloc[cut]['From1D'],
            300,
            alpha = 0.2,
            color = 'forestgreen',
            label="Cut Windows"
        )
        ax.add_patch(rectangle)
    else:
        rectangle = mpl.patches.Rectangle(
            (df_drvml.iloc[cut]['From1D'], 0),
            df_drvml.iloc[cut]['To1D']-df_drvml.iloc[cut]['From1D'],
            300,
            alpha = 0.2,
            color = 'forestgreen',
             )
        ax.add_patch(rectangle)
```

```
ax.set_xlim(15, 33)
ax.set_ylim(-20, 450)
ax.set_ylabel("Intensity in mAU", fontsize=20)
ax.set_xlabel(r"$^{1}$D Retention Time in min", fontsize=20)
ax.legend(loc=2, fontsize=20)

plt.tight_layout()
plt.show()
```

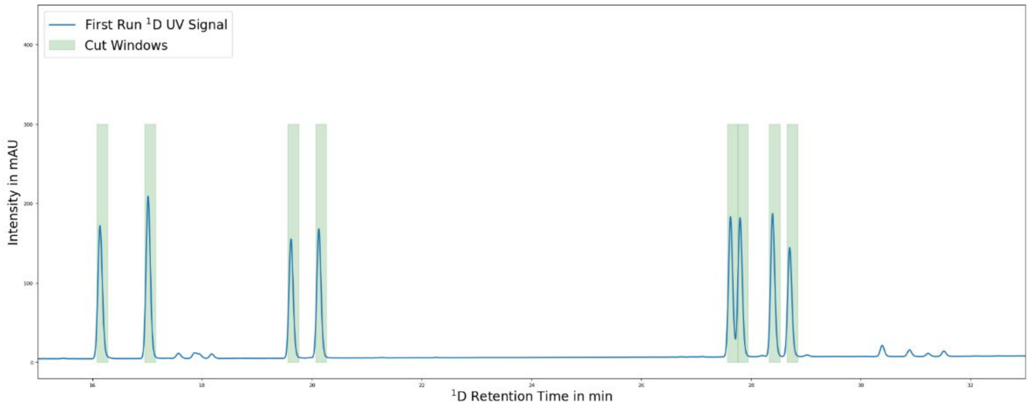

7 Cut the continuously ^{2}D data string into several ^{2}D chromatograms

[11]:
```
x, y, z = akl.make_arrays_mhc_txt(df_drvml, pathlist)
```

```
1 out of 1 Done.
```

8 Generate the transposed matrix of the data for plotting -> ^{2}D on y axis, ^{1}D on x axis

[12]:
```
xT = x.T
yT = y.T
zT = z.T
```

9 Show data

[13]:
```
xT
```

```
[13]:           0          1          2          3          4          5  \
      0  16.175387  17.046914  19.660326  20.165129  27.662673  27.851379

                6          7
      0  28.432264  28.751959
```

```
[14]: yT
```

```
[14]:            0          1          2          3          4          5  \
      0     0.000000   0.000000   0.000000   0.000000   0.000000   0.000000
      1     0.039783   0.039783   0.039800   0.039800   0.039800   0.039800
      2     0.079583   0.079583   0.079583   0.079600   0.079600   0.079600
      3     0.119383   0.119383   0.119383   0.119400   0.119400   0.119383
      4     0.159183   0.159167   0.159183   0.159183   0.159183   0.159183
      ..         ...        ...        ...        ...        ...        ...
      270  10.744767  10.744767  10.744767  10.744783  10.744783  10.744767
      271  10.784567  10.784550  10.784567  10.784567  10.784567  10.784567
      272  10.824350  10.824350  10.824367  10.824367  10.824367  10.824367
      273  10.864150  10.864150  10.864150  10.864167  10.864167  10.864167
      274        NaN  10.903950  10.903950        NaN        NaN  10.903950

                   6          7
      0     0.000000   0.000000
      1     0.039783   0.039800
      2     0.079583   0.079600
      3     0.119383   0.119400
      4     0.159183   0.159183
      ..         ...        ...
      270  10.744767  10.744783
      271  10.784567  10.784567
      272  10.824350  10.824367
      273  10.864150  10.864167
      274        NaN  10.903950

      [275 rows x 8 columns]
```

```
[15]: zT
```

```
[15]:            0          1          2          3          4          5  \
      0     4.388365  34.731809  28.411341   2.662598  36.080379  32.601408
      1     2.661678  37.631099  34.032219   4.388392  23.820268  26.209228
      2     0.593901  42.811576  34.956026   2.663166  19.138513  19.701121
      3     0.048750  34.371251  32.242968   0.644123  19.773581  15.027650
      4     0.001472  28.557019  29.045772   0.691418  22.931923  15.050873
      ..         ...        ...        ...        ...        ...        ...
      270  20.236023  45.621902  32.146221  24.835068  29.164797  16.309432
      271  16.054371  32.711111  27.213352  27.695105  38.129393  14.584334
```

```
272  10.705226  44.636428  22.480857  28.170484  41.494869  17.664524
273   9.409599  63.964944  27.017377  28.207799  35.058427  25.578892
274        NaN  66.549831  35.669211        NaN        NaN  32.328588

             6          7
0    18.021222   4.708498
1    15.290514   3.879419
2    10.797599   2.600530
3    14.290538   1.309660
4    19.821780   0.816866
..         ...        ...
270   8.137699  25.019439
271  13.748595  23.123065
272  29.444553  16.917489
273  40.019110  11.671874
274        NaN   8.808627

[275 rows x 8 columns]
```

10 Generate Mesh Grid for contourf plot

[16]:
```
[xT.values.min(), xT.values.max(), np.nanmin(yT), np.nanmax(yT)]
```

[16]:
```
[16.175386558842035, 28.7519593904605, 0.0, 10.903950000000009]
```

[17]:
```
x_min, x_max, step = 15, 35, 0.05
```

[18]:
```
x_mesh = np.arange(x_min, x_max,  step=step)
```

[19]:
```
y_mesh = y.iloc[1]
```

[20]:
```
X, Y = np.meshgrid(x_mesh, y_mesh)
```

[21]:
```
X.shape
```

[21]:
```
(275, 400)
```

[22]:
```
Y.shape
```

[22]:
```
(275, 400)
```

[23]:
```
Z = pd.DataFrame(np.zeros(Y.shape))
```

11 Fill z data into grid

[24]:
```
for no, number in enumerate(xT.values[0].round(decimals=4)):
    z_index = np.argwhere(np.isclose(number, X[0], atol=step/2))[0][0]
    Z[z_index] = zT[no]
```

12 Remove all z-Values lower than 1 or NaN's

[25]:
```
Z.clip(lower=1, inplace=True)
Z.fillna(1, inplace=True)
```

13 Generate levels - values of contour lines - first lin than log scale

[26]:
```
np.nanmax(Z.values) / 5
level_part1 = np.linspace(1, np.nanmax(Z.values) / 4, 5)
level_part2 = np.logspace(np.log10(np.nanmax(Z.values) / 4), np.log10(np.
 ↪nanmax(Z.values)), 5)
levels = np.append(level_part1, level_part2[1:])
levels
```

[26]:
```
array([1.00000000e+00, 3.93487298e+02, 7.85974595e+02, 1.17846189e+03,
       1.57094919e+03, 2.22165765e+03, 3.14189838e+03, 4.44331530e+03,
       6.28379676e+03])
```

14 Make a 2D plot based on contourf function

[27]:
```
%matplotlib inline
width = 7
height = 5

fig, axs = plt.subplots(2, 2, figsize=(width, height),
                        gridspec_kw={'height_ratios': [3, 1],
                                     'width_ratios': [30, 1],
                                    'wspace': 0.05,
                                    'hspace': 0.15,},
                       )
fig.tight_layout()


labels = [r"$^{1}$D UV Signal"]

for no, measurement in enumerate(list_ch):
    axs[1][0].plot(list_ch[no]["Time"], list_ch[no]["Signal"],
                   label=labels[no], linewidth=1, color="black")
```

```python
for cut in df_drvml.index:
    if cut == 0:
        rectangle = mpl.patches.Rectangle(
            (df_drvml.iloc[cut]['From1D'], 0),
            df_drvml.iloc[cut]['To1D']-df_drvml.iloc[cut]['From1D'],
            300,
            alpha = 0.2,
            color = 'forestgreen',
            label="Cut Windows"
        )
        axs[1][0].add_patch(rectangle)
    else:
        rectangle = mpl.patches.Rectangle(
            (df_drvml.iloc[cut]['From1D'], 0),
            df_drvml.iloc[cut]['To1D']-df_drvml.iloc[cut]['From1D'],
            300,
            alpha = 0.2,
            color = 'forestgreen',
             )
        axs[1][0].add_patch(rectangle)

axs[1][0].legend()
axs[1][0].sharex(axs[0][0])
axs[1][0].set_xlim(15, 34.5)
axs[1][0].set_ylim(-20, 300)


axs[1][0].set_ylabel(r"$^{1}$D Signal (mAU)")
axs[1][0].set_xlabel(r"$^{1}$D Retention Time (min)")

cf = axs[0][0].contourf(
    X,
    Y,
    Z,
    norm=mpl.cm.colors.BoundaryNorm(levels, 256),
    levels=levels,
    cmap=mpl.cm.cool,
)

axs[0][0].annotate('MS2 SWATH 539.5-560.5, EIC 410.171 m/z', xy=(0.6, 0.96),␣
 ↪xycoords='axes fraction',
            size=10, ha='center', va='top',
            bbox=dict(boxstyle='round', fc='w'))

axs[0][0].xaxis.set_minor_locator(mpl.ticker.AutoMinorLocator())
axs[0][0].yaxis.set_minor_locator(mpl.ticker.AutoMinorLocator())
```

```
axs[0][0].set_ylim(4.5, 8.5)
xaxcbar = axs[0][0].set_ylabel(r"$^{2}$D Retention Time (min)")




cbar = fig.colorbar(cf, cax=axs[0][1],  format='%.1e',)
cbar.ax.set_ylabel('$^{2}$D Intensity (cps)')


# inset axes
x1, x2, y1, y2 = 27.5, 28, 5, 8
axins = axs[0][0].inset_axes(
    [0.35, 0.2, 0.25, 0.6],
    xlim=(x1, x2), ylim=(y1, y2))


axins.contourf(
    X,
    Y,
    Z,
    norm=mpl.cm.colors.BoundaryNorm(levels, 256),
    levels=levels,
    cmap=mpl.cm.cool,
)

for label in axins.get_xticklabels():
    label.set_bbox(dict(boxstyle='round',
                        edgecolor="#00ffff",
                        facecolor="#00ffff",
                        alpha=0.7))

for label in axins.get_yticklabels():
    label.set_bbox(dict(boxstyle='round',
                    edgecolor="#00ffff",
                    facecolor="#00ffff",
                    alpha=0.7))

labels = [
    [axs.flat[0], '1a', (15.55, 5.65)],
    [axs.flat[0], '1b', (15.55, 6.1)],
    [axs.flat[0], '2a', (17.6, 6.23)],
    [axs.flat[0], '2b', (17.6, 7.05)],
    [axs.flat[0], '3a', (19, 5.8)],
    [axs.flat[0], '3b', (19, 8)],
    [axs.flat[0], '4a', (20.2, 5.35)],
    [axs.flat[0], '4b', (20.2, 6.95)],
```

```
    [axins, '5a', (27.55, 5.98)],
    [axins, '5b', (27.55, 6.3)],
    [axins, '6a', (27.55, 5.7)],
    [axins, '6b', (27.55, 7.6)],
    [axins, '6a', (27.75, 5.7)],
    [axins, '6b', (27.75, 7.6)],
    [axs.flat[0], '7a', (28.5, 5.2)],
    [axs.flat[0], '7b', (28.5, 6.2)],
    [axs.flat[0], '8a', (29.3, 5.5)],
    [axs.flat[0], '8b', (29.3, 7.45)],
    [axs.flat[2], '1', (15.8, 200)],
    [axs.flat[2], '2', (17.4, 200)],
    [axs.flat[2], '3', (19.3, 200)],
    [axs.flat[2], '4', (20.5, 200)],
    [axs.flat[2], '5', (27.4, 210)],
    [axs.flat[2], '6', (28, 210)],
    [axs.flat[2], '7', (28.4, 220)],
    [axs.flat[2], '8', (28.9, 190)],
]

for label in labels:
    label[0].annotate(label[1], xy =label[2],
                xycoords='data',
                size=10,
                ha='center',
                va='center',
               )


axs[0][0].indicate_inset_zoom(axins, edgecolor="black")

pos00 = axs[0,0].get_position()
pos01 = axs[0,1].get_position()
pos10 = axs[1,0].get_position()
pos11 = axs[1,1].get_position()
pos_xbar = xaxcbar.get_position()

axs[1,1].set_axis_off()
pos = [pos11.x0, pos11.y0, 1-pos11.x0, pos10.height]
ax = fig.add_axes(pos)
ax.set_position(pos)

x_levels = np.arange(0, len(levels))
ax.scatter(x_levels, levels, c=mpl.cm.cool(levels/levels.max()) )
ax.margins(0.2, 0.2)
ax.set_yticks([])
ax.set_xlabel(r"# of levels")
```

```
ax.yaxis.set_label_position("right")
ax.set_ylabel(r"$^2$D Int. (cps)")

plt.savefig("../figures/2dplot_zwix_plus_16mix_ms2_contourf_lin.svg",␣
 ↪bbox_inches="tight")
plt.savefig("../figures/2dplot_zwix_plus_16mix_ms2_contourf_lin.pdf",␣
 ↪bbox_inches="tight")
plt.show()
```

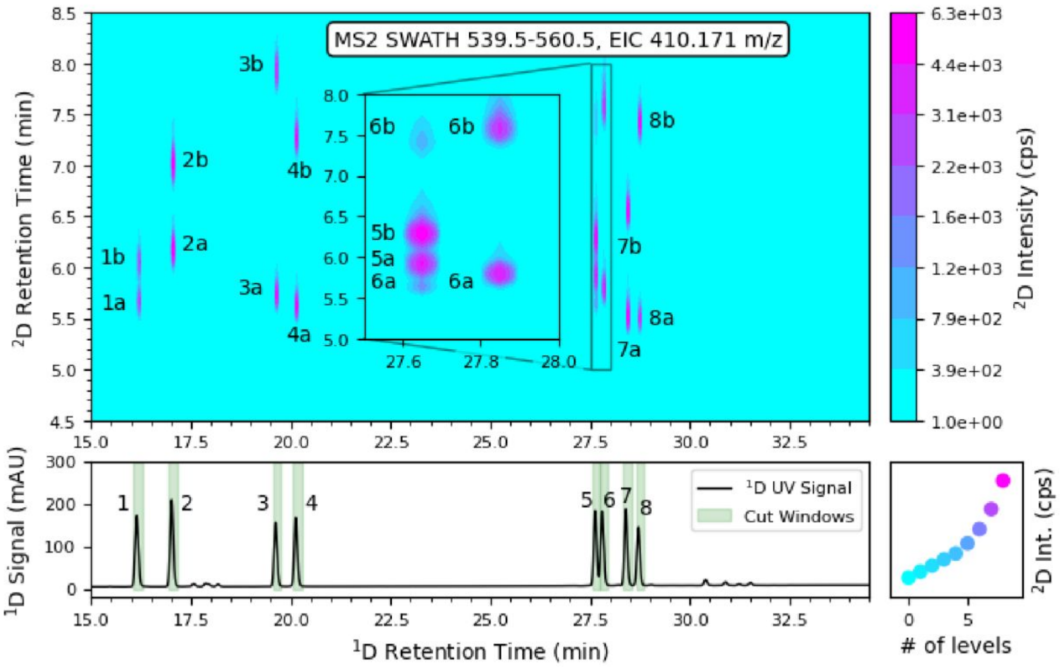

Table S1.Labels of the tetrapeptide of epifadin.

Label	Stereo configuration	Label	Stereo configuration
1	RRRR / SSSS	5	RSSS / SRRR
2	RRRS / SSSR	6	RSSR / SRRS
3	RRSR / SSRS	7	RSRR / SRSS
4	RRSS / SSRR	8	RSRS / SRSR
1a	RRRR	1b	SSSS
2a	SSSR	2b	RRRS
3a	RRSR	3b	SSRS
4a	RRSS	4b	SSRR
5a	RSSS	5b	SRRR
6a	RSSR	6b	SRRS
7a	SRSS	7b	RSRR
8a	SRSR	8b	RSRS

Table S2. 1D chiral HPLC methods.

Method Nr.	Column	Temperature	Flow rate	Mobile Phases	Gradient
28	Daicel CHIRALPAK ZWIX(+), 3 x 150 mm, 3 µm	25 °C	0.5 mL/min	49% ACN, 49% MeOH, 25mM NH_3, 50mM FA	Isocratic 16 min
29	Daicel CHIRALPAK ZWIX(-), 4 x 150 mm, 3 µm	25 °C	0.89 mL/min	49% ACN, 49% MeOH, 25mM NH_3, 50mM FA	Isocratic 16 min
30	Daicel CROWNPAK CR-I(+), 3 x 150 mm, 5 µm	25 °C	0.55 mL/min	A: 100% H_2O + 0.2 % TFA<br>B: 85% ACN, 15% MeOH + 0.2 % TFA	Isocratic 10 min, A: 20%, B: 80%
31	Daicel CROWNPAK CR-I(-), 3 x 150 mm, 5 µm	25 °C	0.55 mL/min	A: 100% H_2O + 0.2 % TFA<br>B: 85% ACN, 15% MeOH + 0.2 % TFA	Isocratic 10 min, A: 20%, B: 80%
32	Astec CHIROBIOTIC T, 4.6 x 250 mm, 5 µm	25 °C	0.5 mL/min	49% ACN, 49% MeOH, 25mM NH_3, 50mM FA	Isocratic 20 min
33	Astec CHIROBIOTIC TAG, 4.6 x 250 mm, 5 µm	25 °C	0.5 mL/min	49% ACN, 49% MeOH, 25mM NH_3, 50mM FA	Isocratic 20 min

Table S3. 1D achiral HPLC screening methods.

Temperature was set to 40 °C.

Method Nr.	Column	Flow rate	Mobile Phases	Gradient
1	Agilent AdvanceBio Peptide Plus, 2.1 x 50 mm, 2.7 µm	0.250 mL/min 2.41 mm/s[1,2]	A: 10% MeOH, 90% H_2O, 10 mM NH_4Ac, pH 5.8<br>B: 70% MeOH, 30% H_2O, 10 mM NH_4Ac, pH 5.8	0 min 0 %B, 11.48 min 50 %B, 11.9 min 90 %B, 13.44 min 90 %B, 13.45 min 100 %B, 18.28 min 100 %B, 18.30 min 0 %B, 23.13 min 0 %B
2	″	0.350 mL/min 3.37 mm/s[1,2]	A: 10% ACN, 90% H_2O, 10 mM NH_4Ac, pH 5.8<br>B: 70% ACN, 30% H_2O, 10 mM NH_4Ac, pH 5.8	0 min 0 %B, 8.2 min 50 %B, 8.5 min 90 %B, 9.6 min 90 %B, 9.61 min 100 %B, 13.06 min 100 %B, 13.07 min 0 %B, 16.52 min 0 %B
3	″	″	A: 10% ACN, 90% H_2O, 20 mM NaH_2PO_4, pH 6<br>B: 70% ACN, 30% H_2O, 20 mM NaH_2PO_4, pH 6	″

[1] Used formula: ((4000/60) * flow_rate) / (column_diameter * π * ε)
[2] A total porosity of 0.5 was assumed for those particles.

4	Agilent AdvanceBio Peptide Plus, 2.1 x 50 mm, 2.7 µm	0.350 mL/min 3.37 mm/s[1,2]	A: 10% ACN, 90% H_2O, 0.1 % AcOH<br>B: 70% ACN, 30% H_2O, 0.1 % AcOH	0 min 0 %B, 8.2 min 50 %B, 8.5 min 90 %B, 9.6 min 90 %B, 9.61 min 100 %B, 13.06 min 100 %B, 13.07 min 0 %B, 16.52 min 0 %B
5	〃	〃	A: 10% ACN, 90% H_2O, 0.1 % TFA<br>B: 70% ACN, 30% H_2O, 0.1 % TFA	〃
6	Agilent Zorbax SB-C18, 2.1 x 100 mm, 1.8 µm	0.250 mL/min 1.85 mm/s[1,3]	A: 10% MeOH, 90% H_2O, 10 mM NH_4Ac, pH 5.8<br>B: 70% MeOH, 30% H_2O, 10 mM NH_4Ac, pH 5.8	0 min 0 %B, 22.96 min 50 %B, 23.80 min 90 %B, 26.88 min 90 %B, 26.91 min 100 %B, 36.57 min 100 %B, 36.60 min 0 %B, 46.26 min 0 %B
7	〃	0.350 mL/min 2.59 mm/s[1,3]	A: 10% ACN, 90% H_2O, 10 mM NH_4Ac, pH 5.8<br>B: 70% ACN, 30% H_2O, 10 mM NH_4Ac, pH 5.8	0 min 0 %B, 16.4 min 50 %B, 17 min 90 %B, 19.2 min 90 %B, 19.22 min 100 %B, 26.12 min 100 %B, 26.14 min 0 %B, 33.00 min 0 %B
8	〃	〃	A: 10% ACN, 90% H_2O, 20 mM NaH_2PO_4, pH 6<br>B: 70% ACN, 30% H_2O, 20 mM NaH_2PO_4, pH 6	〃
9	〃	〃	A: 10% ACN, 90% H_2O, 0.1 % AcOH<br>B: 70% ACN, 30% H_2O, 0.1 % AcOH	〃
10	〃	〃	A: 10% ACN, 90% H_2O, 0.1 % TFA<br>B: 70% ACN, 30% H_2O, 0.1 % TFA	〃
11	Waters Atlantis Premier BEH C18 AX, 2.1 x 50 mm, 2.5 µm	0.250 mL/min 1.85 mm/s[1,3]	A: 10% MeOH, 90% H_2O, 10 mM NH_4Ac, pH 5.8<br>B: 70% MeOH, 30% H_2O, 10 mM NH_4Ac, pH 5.8	0 min 0 %B, 11.48 min 50 %B, 11.9 min 90 %B, 13.44 min 90 %B, 13.45 min 100 %B, 18.28 min 100 %B, 18.30 min 0 %B, 23.13 min 0 %B
12	〃	0.350 mL/min 2.59 mm/s[1,3]	A: 10% ACN, 90% H_2O, 10 mM NH_4Ac, pH 5.8<br>B: 70% ACN, 30% H_2O, 10 mM NH_4Ac, pH 5.8	0 min 0 %B, 8.2 min 50 %B, 8.5 min 90 %B, 9.6 min 90 %B, 9.61 min 100 %B, 13.06 min 100 %B, 13.07 min 0 %B, 16.52 min 0 %
13	〃	〃	A: 10% ACN, 90% H_2O, 20 mM NaH_2PO_4, pH 6<br>B: 70% ACN, 30% H_2O, 20 mM NaH_2PO_4, pH 6	〃

[3] A total porosity of 0.65 was assumed for those particles.

14	„[4]	„	A: 10% ACN, 90% H_2O, 0.1 % FA<br>B: 70% ACN, 30% H_2O, 0.1 % FA	0 min 0 %B, 24.6 min 50 %B, 25.5 min 90 %B, 28.8 min 90 %B, 28.83 min 100 %B, 39.18 min 100 %B, 39.21 min 0 %B, 49.56 min 0 %
15	„	„	A: 10% ACN, 90% H_2O, 0.1 % AcOH<br>B: 70% ACN, 30% H_2O, 0.1 % AcOH	0 min 0 %B, 8.2 min 50 %B, 8.5 min 90 %B, 9.6 min 90 %B, 9.61 min 100 %B, 13.06 min 100 %B, 13.07 min 0 %B, 16.52 min 0 %
16	Waters Atlantis Premier BEH C18 AX,<br>2.1 x 50 mm, 2.5 µm	0.350 mL/min<br>2.59 mm/s[1,3]	A: 10% ACN, 90% H_2O, 0.1 % TFA<br>B: 70% ACN, 30% H_2O, 0.1 % TFA	0 min 0 %B, 8.2 min 50 %B, 8.5 min 90 %B, 9.6 min 90 %B, 9.61 min 100 %B, 13.06 min 100 %B, 13.07 min 0 %B, 16.52 min 0 %
17	Waters XBridge Peptide BEH C18,<br>2.1 x 250 mm, 3.5 µm	0.250 mL/min<br>1.85 mm/s[1,3]	A: 10% MeOH, 90% H_2O, 10 mM NH_4Ac, pH 5.8<br>B: 70% MeOH, 30% H_2O, 10 mM NH_4Ac, pH 5.8	0 min 0 %B, 57.40 min 50 %B, 59.50 min 90 %B, 67.20 min 90 %B, 67.30 min 100 %B, 91.40 min 100 %B, 91.50 min 0 %B, 116.00 min 0 %B
18	„	0.350 mL/min<br>2.59 mm/s[1,3]	A: 10% ACN, 90% H_2O, 10 mM NH_4Ac, pH 5.8<br>B: 70% ACN, 30% H_2O, 10 mM NH_4Ac, pH 5.8	0 min 0 %B, 41 min 50 %B, 42.5 min 90 %B, 48 min 90 %B, 48.01 min 100 %B, 65.30 min 100 %B, 65.31 min 0 %B, 82.60 min 0 %B
19	„	„	A: 10% ACN, 90% H_2O, 20 mM NaH_2PO_4, pH 6<br>B: 70% ACN, 30% H_2O, 20 mM NaH_2PO_4, pH 6	„
20	„	„	A: 10% ACN, 90% H_2O, 0.1 % FA<br>B: 70% ACN, 30% H_2O, 0.1 % FA	„
21	„	„	A: 10% ACN, 90% H_2O, 0.1 % AcOH<br>B: 70% ACN, 30% H_2O, 0.1 % AcOH	„
22	„	„	A: 10% ACN, 90% H_2O, 0.1 % TFA<br>B: 70% ACN, 30% H_2O, 0.1 % TFA	„

Table S4. 1D temperature screening methods.

Method Nr.	Column	Temperature	Flow rate	Mobile Phases	Gradient

[4] Gradient was changed, due to low retention with formic acid.

23	Tandem Waters Atlantis Premier BEH C18 AX,<br>2 x 2.1 x 150 mm, 1.7 µm	15 °C	0.250 mL/min<br>1.85 mm/s[1,3]	A: 10% ACN, 90% H_2O, 0.1 % TFA<br>B: 90% ACN, 10% H_2O, 0.1 % TFA	0 min 0 %B, 86.1 min 50 %B, 89.25 min 90 %B, 89.26 min 100 %B, 125.66 min 100 %B, 125.67 min 0 %B, 162 min 0 %B
24	Waters Atlantis Premier BEH C18 AX,<br>2.1 x 150 mm, 1.7 µm	10 °C	0.350 mL/min<br>2.59 mm/s[1,3]	A: 10% ACN, 90% H_2O, 0.1 % TFA<br>B: 90% ACN, 10% H_2O, 0.1 % TFA	0 min 0 %B, 24.6 min 50 %B, 25.5 min 90 %B, 28.8 min 90 %B, 28.83 min 100 %B, 39.18 min 100 %B, 39.21 min 0 %B, 49.56 min 0 %B
25	″	20 °C	″	″	″
26	″	30 °C	″	″	″
27	″	40 °C	″	″	″

Table S5. 2D HPLC methods (final methods).

Method Nr.	Column	Temperature	Flow rate	Mobile Phases	Gradient
34	^{1}D:<br>Tandem Waters Atlantis Premier BEH C18 AX,<br>2 x 2.1 x 150 mm, 1.7 µm<br>^{2}D:<br>Daicel CHIRALPAK ZWIX(+),<br>3 x 150 mm, 3 µm	^{1}D:<br>15 °C<br>^{2}D:<br>25 °C	^{1}D:<br>0.2 mL/min<br>^{2}D:<br>0.6 mL/min	^{1}D:<br>A: 10% ACN, 90% H_2O, 0.05 % TFA<br>B: 90% ACN, 10% H_2O, 0.05 % TFA<br>^{2}D:<br>A: 98% ACN, 2% H_2O, 2.5 mM NH_3, 5 mM FA<br>B: 49% ACN, 49% MeOH, 2% H_2O 25mM NH_3, 50mM FA	^{1}D:<br>0 min 0 %B, 60 min 35 %B, 60.01 min 100 %B, 97 min 100 %B, 97.01 min 0 %B, 134 min 0 %B<br>^{2}D:<br>0 min 0 %B, 1.1 min 0 %B, 1.15 min 100 %B, 10 min 100 %B, 10.01 min 0 %B, 11 min 0 %B
35	^{1}D:<br>Tandem Waters Atlantis Premier BEH C18 AX,<br>2 x 2.1 x 150 mm, 1.7 µm<br>^{2}D:<br>Daicel CHIRALPAK ZWIX(-),<br>4 x 150 mm, 3 µm	^{1}D:<br>15 °C<br>^{2}D:<br>25 °C	^{1}D:<br>0.2 mL/min<br>^{2}D:<br>1.07 mL/min	^{1}D:<br>A: 10% ACN, 90% H_2O, 0.05 % TFA<br>B: 90% ACN, 10% H_2O, 0.05 % TFA<br>^{2}D:<br>A: 98% ACN, 2% H_2O, 2.5 mM NH_3, 5 mM FA<br>B: 49% ACN, 49% MeOH, 25mM NH_3, 50mM FA	^{1}D:<br>0 min 0 %B, 60 min 35 %B, 60.01 min 100 %B, 97 min 100 %B, 97.01 min 0 %B, 134 min 0 %B<br>^{2}D:<br>0 min 0 %B, 1.1 min 0 %B, 1.15 min 100 %B, 10 min 100 %B, 10.01 min 0 %B, 11 min 0 %B
36	^{1}D:	^{1}D:	^{1}D:	^{1}D:	^{1}D:

	Tandem Waters Atlantis Premier BEH C18 AX,<br>2 x 2.1 x 150 mm, 1.7 µm<br>^{2}D:<br>Daicel CROWNPAK CR-I(+),<br>3 x 150 mm, 5 µm	15 °C<br>^{2}D:<br>25 °C	0.2 mL/min<br>^{2}D:<br>0.55 mL/min	A: 10% ACN, 90% H_2O, 0.05 % TFA<br>B: 90% ACN, 10% H_2O, 0.05 % TFA<br>^{2}D:<br>A: 100% H_2O + 0.2 % TFA<br>B: 85% ACN, 15% MeOH + 0.2 % TFA	0 min 0 %B, 60 min 35 %B, 60.01 min 100 %B, 97 min 100 %B, 97.01 min 0 %B, 134 min 0 %B<br>^{2}D:<br>0 min 0 %B, 1.2 min 0 %B, 1.21 min 20 %B, 10 min 20 %B, 10.01 min 0 %B, 11 min 0 %B
37	^{1}D:<br>Tandem Waters Atlantis Premier BEH C18 AX,<br>2 x 2.1 x 150 mm, 1.7 µm<br>^{2}D:<br>Daicel CROWNPAK CR-I(-),<br>3 x 150 mm, 5 µm	^{1}D:<br>15 °C<br>^{2}D:<br>25 °C	^{1}D:<br>0.2 mL/min<br>^{2}D:<br>0.55 mL/min	^{1}D:<br>A: 10% ACN, 90% H_2O, 0.05 % TFA<br>B: 90% ACN, 10% H_2O, 0.05 % TFA<br>^{2}D:<br>A: 100% H_2O + 0.2 % TFA<br>B: 85% ACN, 15% MeOH + 0.2 % TFA	^{1}D:<br>0 min 0 %B, 60 min 35 %B, 60.01 min 100 %B, 97 min 100 %B, 97.01 min 0 %B, 134 min 0 %B<br>^{2}D:<br>0 min 0 %B, 1.2 min 0 %B, 1.21 min 20 %B, 10 min 20 %B, 10.01 min 0 %B, 11 min 0 %B
38	^{1}D:<br>Tandem Waters Atlantis Premier BEH C18 AX,<br>2 x 2.1 x 150 mm, 1.7 µm<br>^{2}D:<br>Astec CHIROBIOTIC T,<br>4.6 x 250 mm, 5 µm	^{1}D:<br>15 °C<br>^{2}D:<br>25 °C	^{1}D:<br>0.2 mL/min<br>^{2}D:<br>1 mL/min	^{1}D:<br>A: 10% ACN, 90% H_2O, 0.05 % TFA<br>B: 90% ACN, 10% H_2O, 0.05 % TFA<br>^{2}D:<br>A: 98% ACN, 2% H_2O, 2.5 mM NH_3, 5 mM FA<br>B: 49% ACN, 49% MeOH, 25mM NH_3, 50mM FA	^{1}D:<br>0 min 0 %B, 60 min 35 %B, 60.01 min 100 %B, 97 min 100 %B, 97.01 min 0 %B, 134 min 0 %B<br>^{2}D:<br>0 min 0 %B, 0.66 min 0 %B, 0.67 min 100 %B, 10 min 100 %B, 10.01 min 0 %B, 11 min 0 %B
39	^{1}D:<br>Tandem Waters Atlantis Premier BEH C18 AX,<br>2 x 2.1 x 150 mm, 1.7 µm<br>^{2}D:<br>Astec CHIROBIOTIC TAG,<br>4.6 x 250 mm, 5 µm	^{1}D:<br>15 °C<br>^{2}D:<br>25 °C	^{1}D:<br>0.2 mL/min<br>^{2}D:<br>1 mL/min	^{1}D:<br>A: 10% ACN, 90% H_2O, 0.05 % TFA<br>B: 90% ACN, 10% H_2O, 0.05 % TFA<br>^{2}D:<br>A: 98% ACN, 2% H_2O, 2.5 mM NH_3, 5 mM FA<br>B: 49% ACN, 49% MeOH, 25mM NH_3, 50mM FA	^{1}D:<br>0 min 0 %B, 60 min 35 %B, 60.01 min 100 %B, 97 min 100 %B, 97.01 min 0 %B, 134 min 0 %B<br>^{2}D:<br>0 min 0 %B, 0.66 min 0 %B, 0.67 min 100 %B, 10 min 100 %B, 10.01 min 0 %B, 11 min 0 %B

Table S6. Summarized elution pattern of all chiral columns.

Tandem BEH C18 AX	More ^{2}D Retention →											
More ^{1}D Retention ↓	Chirobiotic T		Chirobiotic TAG		Zwix (+)		Zwix (-)		CR-I (+)		CR-I (-)	
RRRR / SSSS	RRRR	SSSS	No sep.		RRRR	SSSS	No sep.		RRRR	SSSS	SSSS	RRRR
RRRS / SSSR	No sep.		SSSR	RRRS	SSSR	RRRS	No sep.		RRRS	SSSR	SSSR	RSSS
RRSR / SSRS	No sep.		No sep.		RRSR	SSRS	SSRS	RRSR	RRSR	SSRS	SSRS	RRSR
RRSS / SSRR	No sep.		SSRR	RRSS	RRSS	SSRR	SSRR	RRSS	RRSS	SSRR	SSRR	RRSS
RSSS / SRRR	No sep.		No sep.		RSSS	SRRR	No sep.		RSSS	SRRR	SRRR	RSSS
RSSR / SRRS	No sep.		RSSR	SRRS	RSSR	SRRS	No sep.		RSSR	SRRS	SRRS	RSSR
RSRR / SRSS	SRSS	RSRR	No sep.		SRSS	RSRR	No sep.		RSRR	SRSS	SRSS	RSRR
RSRS / SRSR	No sep.		SRSR	RSRS	SRSR	RSRS	RSRS	SRSR	RSRS	SRSR	SRSR	RSRS

Table S7. Summarized separation parameters of 1D chiral columns.

t_0 in min, t_R in min, $w_{1/2}$ in min.

Parameter	ZWIX (+)	Parameter	ZWIX (-)	Parameter	CR-I (+)	Parameter	CR-I (-)	Parameter	Chirobiotic T	Parameter	Chirobiotic TAG
$\mathrm{th.}t_0$	1.61	$\mathrm{th.}t_0$	1.61	$\mathrm{th.}t_0$	1.47	$th.\,t_0$	1.47	$\mathrm{th.}t_0$	6.32	$\mathrm{th.}t_0$	6.32
$t_R(RRRR)$	4.59	$t_R(RRRR)$	-	$t_R(RRRR)$	1.78	$t_R(RRRR)$	3.95	$t_R(RRRR)$	8.77	$t_R(RRRR)$	-
$\omega_{1/2}(RRRR)$	0.15	$\omega_{1/2}(RRRR)$	-	$\omega_{1/2}(RRRR)$	0.06	$\omega_{1/2}(RRRR)$	0.21	$\omega_{1/2}(RRRR)$	0.43	$\omega_{1/2}(RRRR)$	-
$t_R(\mathrm{SSSS})$	5.10	$t_R(SSSS)$	-	$t_R(\mathrm{SSSS})$	3.92	$t_R(SSSS)$	1.78	$t_R(\mathrm{SSSS})$	9.78	$t_R(\mathrm{SSSS})$	-
$\omega_{1/2}(SSSS)$	0.17	$\omega_{1/2}(SSSS)$	-	$\omega_{1/2}(SSSS)$	0.17	$\omega_{1/2}(SSSS)$	0.06	$\omega_{1/2}(SSSS)$	0.59	$\omega_{1/2}(SSSS)$	-
$\mathrm{k}(RRRR)$	1.85	$\mathrm{k}(RRRR)$	-	$\mathrm{k}(RRRR)$	0.10	$k\ (RRRR)$	1.70	$\mathrm{k}(RRRR)$	0.39	$\mathrm{k}(RRRR)$	-
$\mathrm{k}(SSSS)$	2.16	$\mathrm{k}(SSSS)$	-	$\mathrm{k}(SSSS)$	1.43	$k\ (SSSS)$	0.21	$\mathrm{k}(SSSS)$	0.55	$\mathrm{k}(SSSS)$	-
$\alpha_{RRRR/SSSS}$	1.17	$\alpha_{RRRR/SSSS}$	-	$\alpha_{RRRR/SSSS}$	13.71	$\alpha_{SSSS/RRRR}$	7.97	$\alpha_{RRRR/SSSS}$	1.41	$\alpha_{RRRR/SSSS}$	-
$R_{s,RRRR/SSSS}$	1.88	$R_{s,RRRR/SSSS}$	-	$R_{s,RRRR/SSSS}$	10.95	$R_{s,SSSS/RRRR}$	9.34	$R_{s,RRRR/SSSS}$	1.17	$R_{s,RRRR/SSSS}$	-
$t_R(SSSR)$	4.92	$t_R(SSSR)$	-	$t_R(SSSR)$	3.61	$t_R(SSSR)$	1.79	$t_R(SSSR)$	-	$t_R(SSSR)$	10.51
$\omega_{1/2}(SSSR)$	0.17	$\omega_{1/2}(SSSR)$	-	$\omega_{1/2}(SSSR)$	0.15	$\omega_{1/2}(SSSR)$	0.06	$\omega_{1/2}(SSSR)$	-	$\omega_{1/2}(SSSR)$	0.75
$t_R(RRRS)$	5.79	$t_R(RRRS)$	-	$t_R(RRRS)$	1.80	$t_R(RRRS)$	3.65	$t_R(RRRS)$	-	$t_R(RRRS)$	13.63
$\omega_{1/2}(SSSR)$	0.22	$\omega_{1/2}(SSSR)$	-	$\omega_{1/2}(SSSR)$	0.06	$\omega_{1/2}(RRRS)$	0.15	$\omega_{1/2}(SSSR)$	-	$\omega_{1/2}(SSSR)$	1.02
$k(RRRS)$	2.59	$k(RRRS)$	-	$k(RRRS)$	1.24	$k\ (RRRS)$	1.26	$k(RRRS)$	-	$k(RRRS)$	1.16
$k(SSSR)$	2.05	$k(SSSR)$	-	$k(SSSR)$	0.12	$k\ (SSSR)$	0.11	$k(SSSR)$	-	$k(SSSR)$	0.66
$\alpha_{SSSR/RRRS}$	1.26	$\alpha_{SSSR/RRRS}$	-	$\alpha_{RRRS/SSSR}$	10.61	$\alpha_{SSSR/RRRS}$	11.36	$\alpha_{SSSR/RRRS}$	-	$\alpha_{SSSR/RRRS}$	1.74

$R_{s,SSSR/RRRS}$	2.63	$R_{s,SSSR/RRRS}$	-	$R_{s,RRRS/SSSR}$	10.14	$R_{s,SSSR/RRRS}$	10.23	$R_{s,SSSR/RRRS}$	-	$R_{s,SSSR/RRRS}$	2.07
$t_R(RRSR)$	4.45	$t_R(RRSR)$	2.97	$t_R(RRSR)$	1.77	$t_R(RRSR)$	2.52	$t_R(RRSR)$	-	$t_R(RRSR)$	-
$\omega_{1/2}(RRSR)$	0.14	$\omega_{1/2}(RRSR)$	0.38	$\omega_{1/2}(RRSR)$	0.06	$\omega_{1/2}(RRSR)$	0.09	$\omega_{1/2}(RRSR)$	-	$\omega_{1/2}(RRSR)$	-
$t_R(SSRS)$	6.89	$t_R(SSRS)$	2.32	$t_R(SSRS)$	2.51	$t_R(SSRS)$	1.76	$t_R(SSRS)$	-	$t_R(SSRS)$	-
$\omega_{1/2}(SSRS)$	0.24	$\omega_{1/2}(SSRS)$	0.33	$\omega_{1/2}(SSRS)$	0.12	$\omega_{1/2}(SSRS)$	0.06	$\omega_{1/2}(SSRS)$	-	$\omega_{1/2}(SSRS)$	-
$k(RRSR)$	1.76	$k(RRSR)$	0.84	$k(RRSR)$	0.10	$k\ (RRSR)$	0.57	$k(RRSR)$	-	$k(RRSR)$	-
$k(SSRS)$	3.28	$k(SSRS)$	0.44	$k(SSRS)$	0.56	$k\ (SSRS)$	0.09	$k(SSRS)$	-	$k(SSRS)$	-
$\alpha_{RRSR/SSRS}$	1.86	$\alpha_{SSRS/RRSR}$	1.92	$\alpha_{RRSR/SSRS}$	5.67	$\alpha_{SSRS/RRSR}$	6.03	$\alpha_{RRSR/SSRS}$	-	$\alpha_{RRSR/SSRS}$	-
$R_{s,RRSR/SSRS}$	7.56	$R_{s,SSRS/RRSR}$	1.08	$R_{s,RRSR/SSRS}$	4.84	$R_{s,SSRS/RRSR}$	5.98	$R_{s,RRSR/SSRS}$	-	$R_{s,RRSR/SSRS}$	-
$t_R(RRSS)$	4.37	$t_R(RRSS)$	2.94	$t_R(RRSS)$	1.79	$t_R(RRSS)$	2.54	$t_R(RRSS)$	-	$t_R(RRSS)$	12.58
$\omega_{1/2}(RRSS)$	0.15	$\omega_{1/2}(RRSS)$	0.35	$\omega_{1/2}(RRSS)$	0.07	$\omega_{1/2}(RRSS)$	0.10	$\omega_{1/2}(RRSS)$	-	$\omega_{1/2}(RRSS)$	1.07
$t_R(SSRR)$	6.17	$t_R(SSRR)$	2.32	$t_R(SSRR)$	2.53	$t_R(SSRR)$	1.78	$t_R(SSRR)$	-	$t_R(SSRR)$	11.27
$\omega_{1/2}(SSRR)$	0.20	$\omega_{1/2}(SSRR)$	0.3	$\omega_{1/2}(SSRR)$	0.11	$\omega_{1/2}(SSRR)$	0.06	$\omega_{1/2}(SSRR)$	-	$\omega_{1/2}(SSRR)$	0.75
$k(RRSS)$	1.71	$k(RRSS)$	0.82	$k(RRSS)$	0.11	$k\ (RRSS)$	0.57	$k(RRSS)$	-	$k(RRSS)$	0.99
$k(SSRR)$	2.83	$k(SSRR)$	0.44	$k(SSRR)$	0.57	$k\ (SSRR)$	0.11	$k(SSRR)$	-	$k(SSRR)$	0.78
$\alpha_{RRSS/SSRR}$	1.65	$\alpha_{SSRR/RRSS}$	1.88	$\alpha_{RRSS/SSRR}$	5.07	$\alpha_{SSRR/RRSS}$	5.44	$\alpha_{RRSS/SSRR}$	-	$\alpha_{SSRR/RRSS}$	1.26
$R_{s,RRSS/SSRR}$	6.05	$R_{s,SSRR/RRSS}$	1.12	$R_{s,RRSS/SSRR}$	4.87	$R_{s,SSRR/RRSS}$	5.55	$R_{s,RRSS/SSRR}$	-	$R_{s,SSRR/RRSS}$	0.85
$t_R(SRSS)$	4.22	$t_R(SRSS)$	-	$t_R(SRSS)$	2.50	$t_R(SRSS)$	2.33	$t_R(SRSS)$	9.04	$t_R(SRSS)$	-
$\omega_{1/2}(SRSS)$	0.16	$\omega_{1/2}(SRSS)$	-	$\omega_{1/2}(SRSS)$	0.09	$\omega_{1/2}(SRSS)$	0.10	$\omega_{1/2}(SRSS)$	0.41	$\omega_{1/2}(SRSS)$	-
$t_R(RSRR)$	5.28	$t_R(RSRR)$	-	$t_R(RSRR)$	2.36	$t_R(RSRR)$	2.47	$t_R(RSRR)$	9.69	$t_R(RSRR)$	-
$\omega_{1/2}(RSRR)$	0.19	$\omega_{1/2}(RSRR)$	-	$\omega_{1/2}(RSRR)$	0.09	$\omega_{1/2}(RSRR)$	0.10	$\omega_{1/2}(RSRR)$	0.51	$\omega_{1/2}(RSRR)$	-
$k(SRSS)$	1.62	$k(SRSS)$	-	$k(SRSS)$	0.55	$k\ (SRSS)$	0.45	$k(SRSS)$	0.43	$k(SRSS)$	-

$k(RSRR)$	2.28	$k(RSRR)$	-	$k(RSRR)$	0.46	$k\ (RSRR)$	0.53	$k(RSRR)$	0.53	$k(RSRR)$	-
$\alpha_{SRSS/RSRR}$	1.41	$\alpha_{SRSS/RSRR}$	-	$\alpha_{RSRR/SRSS}$	1.19	$\alpha_{SRSS/RSRR}$	1.19	$\alpha_{SRSS/RSRR}$	1.24	$\alpha_{SRSS/RSRR}$	-
$R_{s,SRSS/RSRR}$	3.56	$R_{s,SRSS/RSRR}$	-	$R_{s,RSRR/SRSS}$	0.93	$R_{s,SRSS/RSRR}$	0.81	$R_{s,SRSS/RSRR}$	0.83	$R_{s,SRSS/RSRR}$	-
$t_R(SRSR)$	4.18	$t_R(SRSR)$	2.52	$t_R(SRSR)$	2.64	$t_R(SRSR)$	2.41	$t_R(SRSR)$	-	$t_R(SRSR)$	12.05
$\omega_{1/2}(SRSR)$	0.15	$\omega_{1/2}(SRSR)$	0.36	$\omega_{1/2}(SRSR)$	0.10	$\omega_{1/2}(SRSR)$	0.09	$\omega_{1/2}(SRSR)$	-	$\omega_{1/2}(SRSR)$	0.70
$t_R(RSRS)$	6.29	$t_R(RSRS)$	2.22	$t_R(RSRS)$	2.45	$t_R(RSRS)$	2.58	$t_R(RSRS)$	-	$t_R(RSRS)$	13.67
$\omega_{1/2}(RSRS)$	0.19	$\omega_{1/2}(RSRS)$	0.22	$\omega_{1/2}(RSRS)$	0.10	$\omega_{1/2}(RSRS)$	0.10	$\omega_{1/2}(RSRS)$	-	$\omega_{1/2}(RSRS)$	1.00
$k(SRSR)$	1.59	$k(SRSR)$	0.56	$k(SRSR)$	0.64	$k(SRSR)$	0.50	$k(SRSR)$	-	$k(SRSR)$	0.91
$k(RSRS)$	2.90	$k(RSRS)$	0.38	$k(RSRS)$	0.52	$k(RSRS)$	0.60	$k(RSRS)$	-	$k(RSRS)$	1.16
$\alpha_{SRSR/RSRS}$	1.82	$\alpha_{RSRS/SRSR}$	1.49	$\alpha_{RSRS/SRSR}$	0.82	$\alpha_{SRSR/RSRS}$	0.82	$\alpha_{SRSR/RSRS}$	-	$\alpha_{SRSR/RSRS}$	0.78
$R_{s,SRSR/RSRS}$	7.30	$R_{s,RSRS/SRSR}$	0.61	$R_{s,RSRS/SRSR}$	1.12	$R_{s,SRSR/RSRS}$	1.05	$R_{s,SRSR/RSRS}$	-	$R_{s,SRSR/RSRS}$	1.12
$t_R(RSSR)$	4.58	$t_R(RSSR)$	-	$t_R(RSSR)$	2.35	$t_R(RSSR)$	2.69	$t_R(RSSR)$	-	$t_R(RSSR)$	11.95
$\omega_{1/2}(RSSR)$	0.15	$\omega_{1/2}(RSSR)$	-	$\omega_{1/2}(RSSR)$	0.09	$\omega_{1/2}(RSSR)$	0.09	$\omega_{1/2}(RSSR)$	-	$\omega_{1/2}(RSSR)$	0.74
$t_R(SRRS)$	6.54	$t_R(SRRS)$	-	$t_R(SRRS)$	2.71	$t_R(SRRS)$	2.34	$t_R(SRRS)$	-	$t_R(SRRS)$	13.44
$\omega_{1/2}(SRRS)$	0.26	$\omega_{1/2}(SRRS)$	-	$\omega_{1/2}(SRRS)$	0.11	$\omega_{1/2}(SRRS)$	0.09	$\omega_{1/2}(SRRS)$	-	$\omega_{1/2}(SRRS)$	0.90
$k(RSSR)$	1.84	$k(RSSR)$	-	$k(RSSR)$	0.46	$k\ (RSSR)$	0.67	$k(RSSR)$	-	$k(RSSR)$	0.89
$k(SRRS)$	3.06	$k(SRRS)$	-	$k(SRRS)$	0.68	$k\ (SRRS)$	0.45	$k(SRRS)$	-	$k(SRRS)$	1.13
$\alpha_{RSSR/SRRS}$	1.66	$\alpha_{RSSR/SRRS}$	-	$\alpha_{RSSR/SRRS}$	1.49	$\alpha_{SRRS/RSSR}$	1.49	$\alpha_{RSSR/SRRS}$	-	$\alpha_{RSSR/SRRS}$	1.26
$R_{s,RSSR/SRRS}$	5.62	$R_{s,RSSR/SRRS}$	-	$R_{s,RSSR/SRRS}$	2.08	$R_{s,SRRS/RSSR}$	2.39	$R_{s,RSSR/SRRS}$	-	$R_{s,RSSR/SRRS}$	1.07
$t_R(RSSS)$	4.75	$t_R(RSSS)$	-	$t_R(RSSS)$	2.34	$t_R(RSSS)$	2.76	$t_R(RSSS)$	-	$t_R(RSSS)$	-
$\omega_{1/2}(RSSS)$	0.16	$\omega_{1/2}(RSSS)$	-	$\omega_{1/2}(RSSS)$	0.09	$\omega_{1/2}(RSSS)$	0.11	$\omega_{1/2}(RSSS)$	-	$\omega_{1/2}(RSSS)$	-
$t_R(SRRR)$	5.17	$t_R(SRRR)$	-	$t_R(SRRR)$	2.78	$t_R(SRRR)$	2.31	$t_R(SRRR)$	-	$t_R(SRRR)$	-

$\omega_{1/2}(SRRR)$	0.19	$\omega_{1/2}(SRRR)$	-	$\omega_{1/2}(SRRR)$	0.11	$\omega_{1/2}(SRRR)$	0.09	$\omega_{1/2}(SRRR)$	-	$\omega_{1/2}(SRRR)$	-
$k(RSSS)$	1.95	$k(RSSS)$	-	$k(RSSS)$	0.45	$k\ (RSSS)$	0.71	$k(RSSS)$	-	$k(RSSS)$	-
$k(SRRR)$	2.21	$k(SRRR)$	-	$k(SRRR)$	0.72	$k\ (SRRR)$	0.44	$k(SRRR)$	-	$k(SRRR)$	-
$\alpha_{RSSS/SRRR}$	1.13	$\alpha_{RSSS/SRRR}$	-	$\alpha_{RSSS/SRRR}$	1.61	$\alpha_{SRRR/RSSS}$	1.63	$\alpha_{RSSS/SRRR}$	-	$\alpha_{RSSS/SRRR}$	-
$R_{s,RSSS/SRRR}$	1.41	$R_{s,RSSS/SRRR}$	-	$R_{s,RSSS/SRRR}$	2.61	$R_{s,SRRR/RSSS}$	2.63	$R_{s,RSSS/SRRR}$	-	$R_{s,RSSS/SRRR}$	-

Table S8. Separation parameters of 1D column screening.

Meth-ods No:	$th.t_0$ (min)	$t_R(1)$ (min)	$\omega_{1/2}(1)$ (min)	$t_R(2)$ (min)	$\omega_{1/2}(2)$ (min)	$t_R(3)$ (min)	$\omega_{1/2}(3)$ (min)	$t_R(4)$ (min)	$\omega_{1/2}(4)$ (min)	$t_R(5)$ (min)	$\omega_{1/2}(5)$ (min)	$t_R(6)$ (min)	$\omega_{1/2}(6)$ (min)	$t_R(7)$ (min)	$\omega_{1/2}(7)$ (min)	$t_R(3)$ (min)	$\omega_{1/2}(3)$ (min)	$R_s(1,2)$, $R_s(2,3)$, $R_s(3,4)$, $R_s(4,5)$, $R_s(5,6)$, $R_s(6,7)$, $R_s(7,8)$
1	0.53	nap.	nap.	nap.	nap.	nap.	nap.	nap.	nap.	nap.	nap.	nap.	nap.	nap.	nap.	nap.	nap.	-
2	0.38	4.06	0.07	4.06	0.07	4.06	0.07	4.18	0.06	4.79	0.04	4.85	0.05	4.92	0.06	4.92	0.06	0.00, 0.00, 1.05, 6.92, 0.74, 0.80, 0.00
3	0.38	3.60	0.04	3.60	0.04	3.65	0.04	3.76	0.04	3.85	0.03	3.94	0.03	3.97	0.03	4.01	0.03	0.00, 0.85, 1.53, 1.47, 1.79,

																			0.64,<br>0.64
4	0.38	2.54	0.04	2.61	0.06	2.61	0.06	2.61	0.06	3.61	0.05	3.71	0.05	3.79	0.05	3.79	0.05	0.83,<br>0.00,<br>0.00,<br>10.46,<br>1.10,<br>0.96,<br>0.00
5	0.38	1.70	0.05	1.80	0.05	2.17	0.05	2.22	0.06	3.13	0.07	3.13	0.07	3.27	0.08	3.27	0.08	1.12,<br>4.35,<br>0.56,<br>8.18,<br>0.00,<br>1.14,<br>0.00
6	0.78	15.56	0.08	15.69	0.12	15.69	0.12	16.29	0.09	21.33	0.08	21.57	0.08	21.79	0.10	21.79	0.10	0.84,<br>0.00,<br>3.34,<br>34.06,<br>1.79,<br>1.50,<br>0.00
7	0.55	8.06	0.08	8.06	0.08	8.06	0.08	8.28	0.04	9.59	0.04	9.71	0.04	9.85	0.05	9.85	0.05	0.00,<br>0.00,<br>2.21,<br>19.05,<br>1.80<br>1.76<br>0.00
8	0.55	4.86	0.04	4.91	0.06	4.91	0.06	5.15	0.05	6.19	0.04	6.32	0.04	6.47	0.04	6.51	0.04	0.68,<br>0.00,

																		2.65,<br>14.05,<br>1.94,<br>2.20,<br>0.55
9	0.55	6.22	0.09	6.43	0.09	6.91	0.18	6.91	0.18	8.92	0.12	8.92	0.12	9.25	0.20	9.25	0.20	1.42,<br>2.12,<br>0.00,<br>8.01,<br>0.00,<br>1.22,<br>0.00
10	0.55	5.11	0.04	5.30	0.04	6.04	0.04	6.17	0.04	8.10	0.06	8.10	0.06	8.44	0.04	8.51	0.05	2.58,<br>10.11,<br>1.72,<br>21.06,<br>0.00,<br>3.75,<br>0.84
11	0.53	10.97	0.08	11.42	0.08	11.30	0.08	11.42	0.09	12.33	0.07	12.47	0.11	12.47	0.11	12.47	0.11	3.25,<br>0.91,<br>0.88,<br>6.63,<br>0.92,<br>0.00,<br>0.00
12	0.38	5.44	0.04	5.44	0.04	5.50	0.04	5.59	0.03	5.64	0.03	5.73	0.02	5.75	0.03	5.78	0.03	0.00,<br>0.95,<br>1.47,<br>0.97,<br>1.90,<br>0.38,

																		0.59
13	0.38	2.28	0.07	2.28	0.07	2.28	0.07	2.41	0.06	2.92	0.05	2.98	0.05	3.07	0.07	3.07	0.07	0.00, 0.00, 1.10, 5.35, 0.79, 0.83, 0.00
14	0.38	2.91	0.03	2,95	0,03	2,99	0.03	3.07	0.03	3.93	0.03	4.05	0.03	4.21	0.03	4.21	0.03	0.85, 0.81, 1.71, 17.41, 2.46, 3.01, 0.00
15	0.38	nap.	nap.	1.94	0.04	2.10	0.04	2.26	0.04	4.63	0.04	4.85	0.04	4.93	0.04	4.93	0.04	-, 2.41, 2.37, 34.97, 3.49, 1.28, 0.00
16	0.38	2.40	0.03	2.50	0.04	2.88	0.03	2.94	0.03	3.91	0.04	3.91	0.04	4.03	0.03	4.06	0.03	1.64, 6.05, 1.10, 14.64, 0.00, 2.03, 0.50
17	2.63	36.28	0.26	36.28	0.26	36.45	0.21	38.14	0.20	50.23	0.19	50.83	0.17	51.36	0.19	51.36	0.19	0.00, 0.41, 4.96,

																		36.84, 1.95, 1.73, 0.00
18	1,88	20.73	0.09	20.92	0.10	20.92	0.10	21.54	0.09	24.38	0.09	24.70	0.08	25.08	0.08	25.11	0.08	1.19, 0.00, 3.92, 18.81, 2.20, 2.64, 0.21
19	1.88	11.71	0.09	11.91	0.10	11.91	0.10	12.54	0.10	14.75	0.09	15.10	0.08	15.50	0.09	15.59	0.08	1.22, 0.00, 3.75, 13.81, 2.35, 2.74, 0.67
20	1.88	7.24	0.08	7.59	0.09	9.14	0.08	9.40	0.08	14.46	0.14	14.46	0.14	15.27	0.12	15.49	0.15	2.53, 11.32, 1.93, 26.41, 0.00, 3.63, 0.93
21	1.88	16.12	0.12	16.60	0.12	17.89	0.17	18.15	0.20	23.15	0.19	23.15	0.19	23.71	0.18	23.95	0.25	2.42, 5.25, 0.84, 15.20, 0.00, 1.78, 0.63

22	1.88	11.64	0.09	12.09	0.09	13.86	0.09	14.17	0.10	19.39	0.13	19.39	0.13	20.17	0.08	20.35	0.10	2.81,<br>11.38,<br>1.99,<br>27.24,<br>0.00,<br>4,27,<br>1.14

Table S9. Separation parameters of temperature screening and tandem column.

Th. t_0 in min, t_R in min, $w_{1/2}$ in min.

Separation parameters	C18 AX (2.1 x 150 mm, 1.7 µm), 40 °C, method 27	C18 AX (2.1 x 150 mm, 1.7 µm), 30 °C, method 26	C18 AX (2.1 x 150 mm, 1.7 µm), 20 °C, method 25	C18 AX (2.1 x 150 mm, 1.7 µm), 10 °C, method 24	C18 AX (2 x 2.1 x 150 mm, 1.7 µm), 30 °C, method 23
$\mathrm{th}.t_0$	1.128	1.128	1.128	1.128	3.949
$t_R(1)$	4.411	4.836	5.402	5.982	16.239
$\omega_{1/2}(1)$	0.041	0.044	0.049	0.056	0.115
$t_R(2)$	4.643	5.106	5.717	6.342	17.373
$\omega_{1/2}(2)$	0.041	0.043	0.048	0.052	0.111

$t_R(3)$	5.555	6.073	6.736	7.402	20.796
$\omega_{1/2}(3)$	0.042	0.044	0.047	0.051	0.108
$t_R(4)$	5.712	6.247	6.927	7.610	21.452
$\omega_{1/2}(4)$	0.041	0.044	0.046	0.050	0.108
$t_R(5)$	8.438	9.050	9.793	10.527	31.301
$\omega_{1/2}(5)$	0.0399	0.0413	0.0436	0.0455	0.1084
$t_R(6)$	8.490	9.107	9.856	10.595	31.532
$\omega_{1/2}(6)$	0.045	0.046	0.048	0.049	0.113
$t_R(7)$	8.822	9.412	10.130	10.838	32.315
$\omega_{1/2}(7)$	0.044	0.045	0.047	0.048	0.110
$t_R(7)$	8.916	9.514	10.240	10.956	32.740
$\omega_{1/2}(7)$	0.046	0.047	0.048	0.049	0.111
$R_s(1,2)$	3.898	3.665	3.850	3.898	5.893
$R_s(2,3)$	12.136	13.128	12.746	12.136	18.355
$R_s(3,4)$	2.441	2.349	2.417	2.441	3.563
$R_s(4,5)$	36.026	38.859	37.481	36.026	53.470
$R_s(5,6)$	0.843	0.768	0.814	0.843	1.227
$R_s(6,7)$	2.924	3.936	3.420	2.924	4.123
$R_s(7,8)$	1.423	1.309	1.370	1.423	2.263

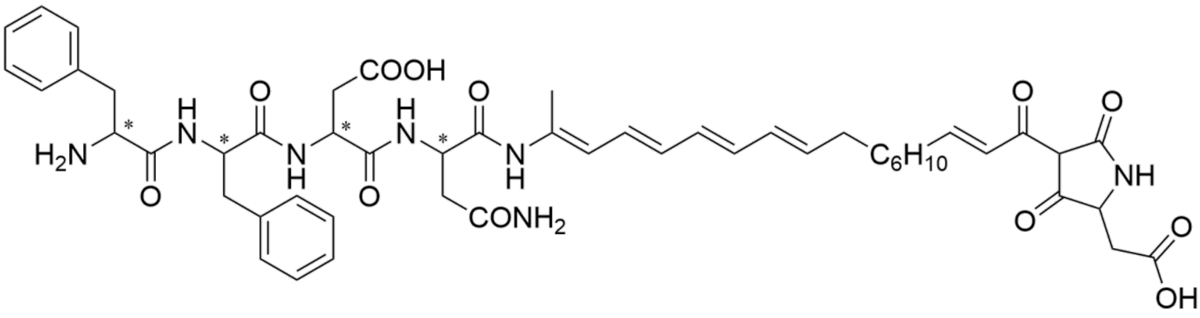

Figure S1. Structure of epifadin.

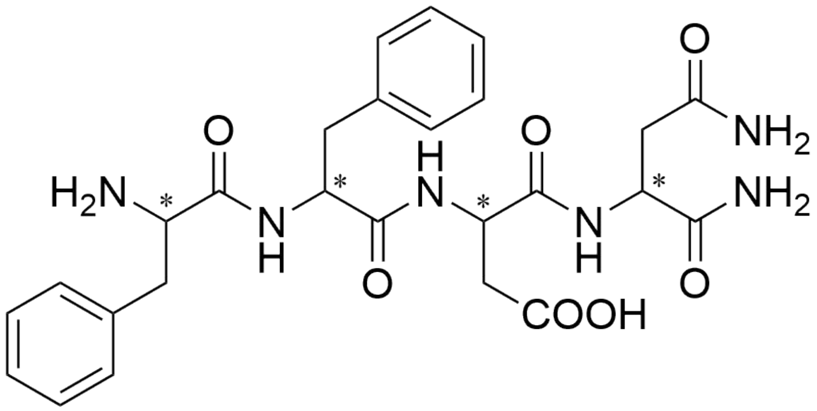

Figure S2. Structure of the tetrapeptide of epifadin.

$[M+H]^+$ calculated for $C_{26}H_{32}N_6O_7$, 541.2405; found 541.2422 (-3.0 ppm err; 3.4 mSigma)
$[M+Na]^+$ calculated for $C_{26}H_{32}N_6O_7$, 563.2225; found 563.2237 (-2.3 ppm err; 7.2 mSigma)

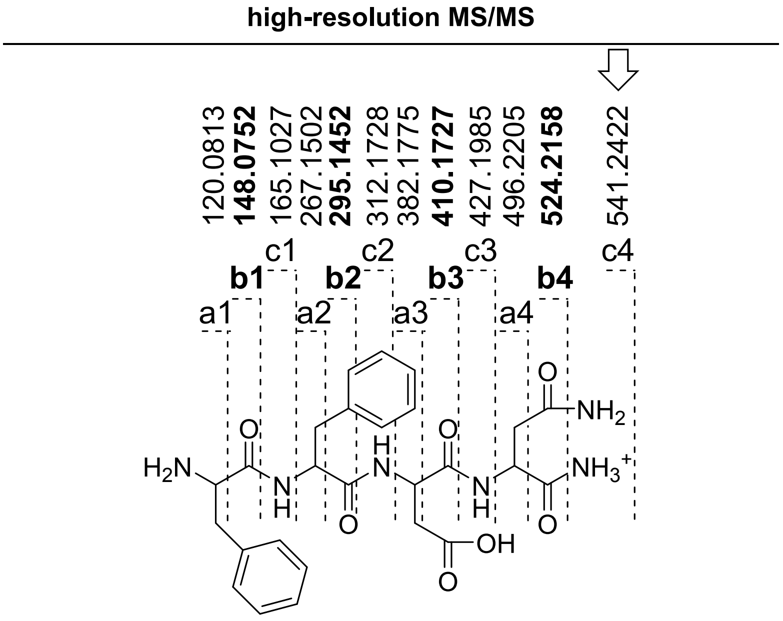

Figure S3. Fragment ion analysis for tetrapeptide of epifadin.

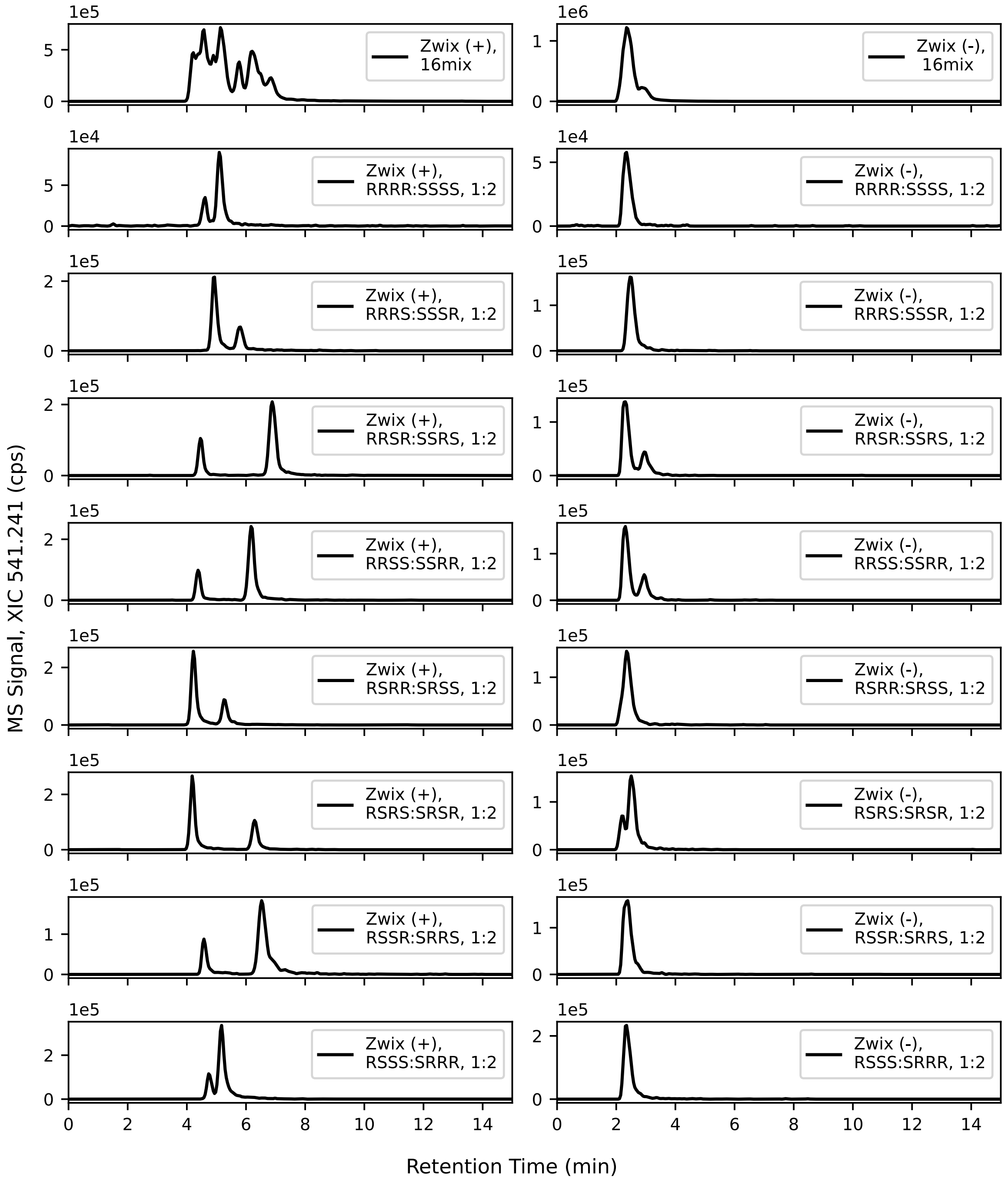

Figure S4. 1D chiral separations with zwix columns.

. 1D chiral separations of all enantiomer pairs of the tetrapeptide of epifadin with the ZWIX columns. ZWIX (+), method: 28. ZWIX (-), method: 29

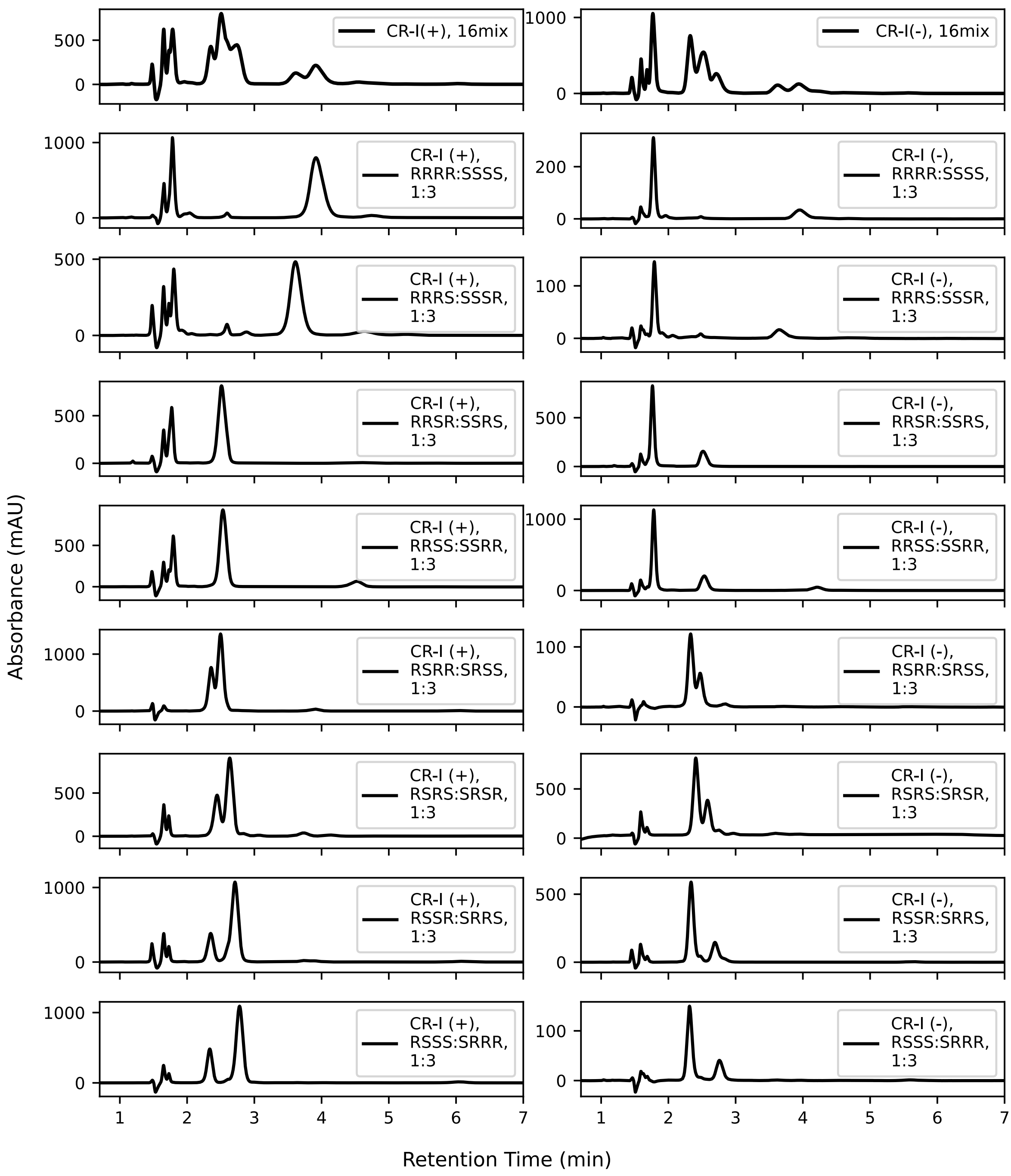

Figure S5. 1D chiral separations with Crownether columns.

1D chiral separations of all enantiomer pairs of the tetrapeptide of epifadin with the Crownether columns. CR-I (+), method: 30. CR-I (-), method: 31.

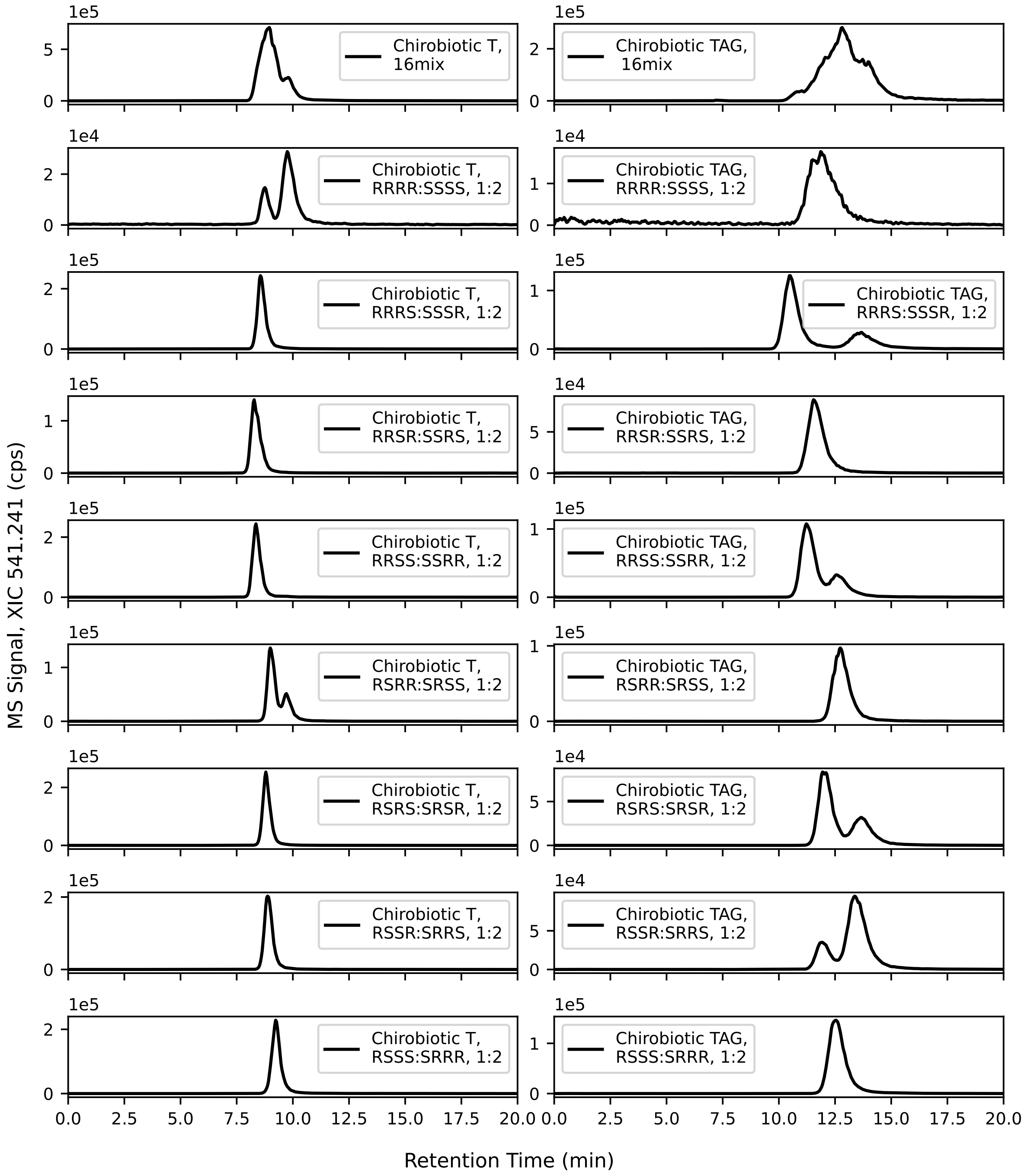

Figure S6. 1D chiral separations with CHIROBIOTIC columns.

1D chiral separations of all enantiomer pairs of the tetrapeptide of epifadin with the CHIROBIOTIC columns. Chirobiotic T, method: 32. Chirobiotic TAG, method: 33.

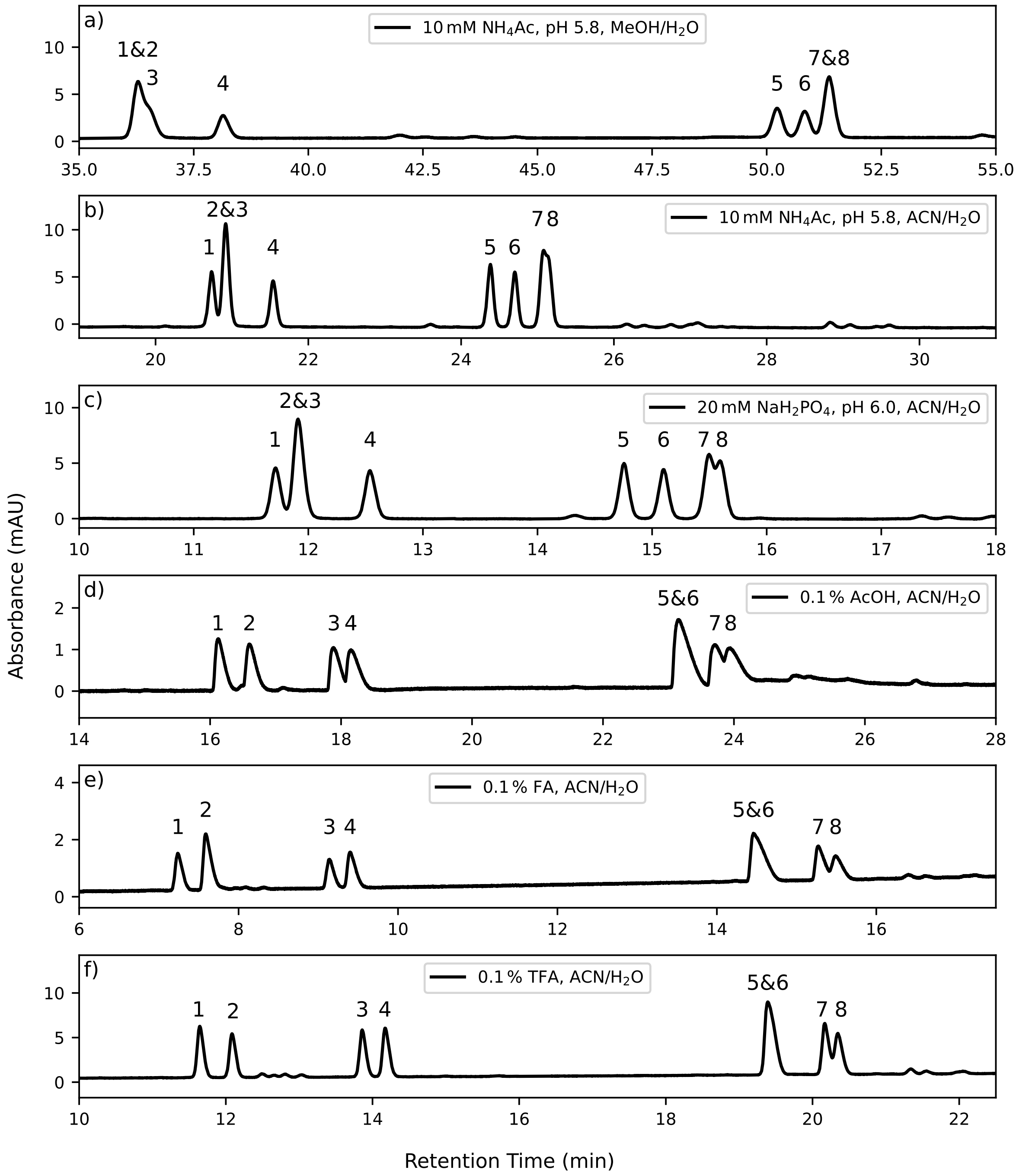

Figure S7. 1D achiral separations with Waters XBridge Peptide BEH C18.

1D achiral separations of the 16-isomer standard mix of the tetrapeptide of epifadin with the Waters X Bridge Peptide BEH C18 (2.1 x 250 mm, 3.5 µm). Methods: a) 17, b) 18, c) 19, d) 20, e) 21, f) 22.

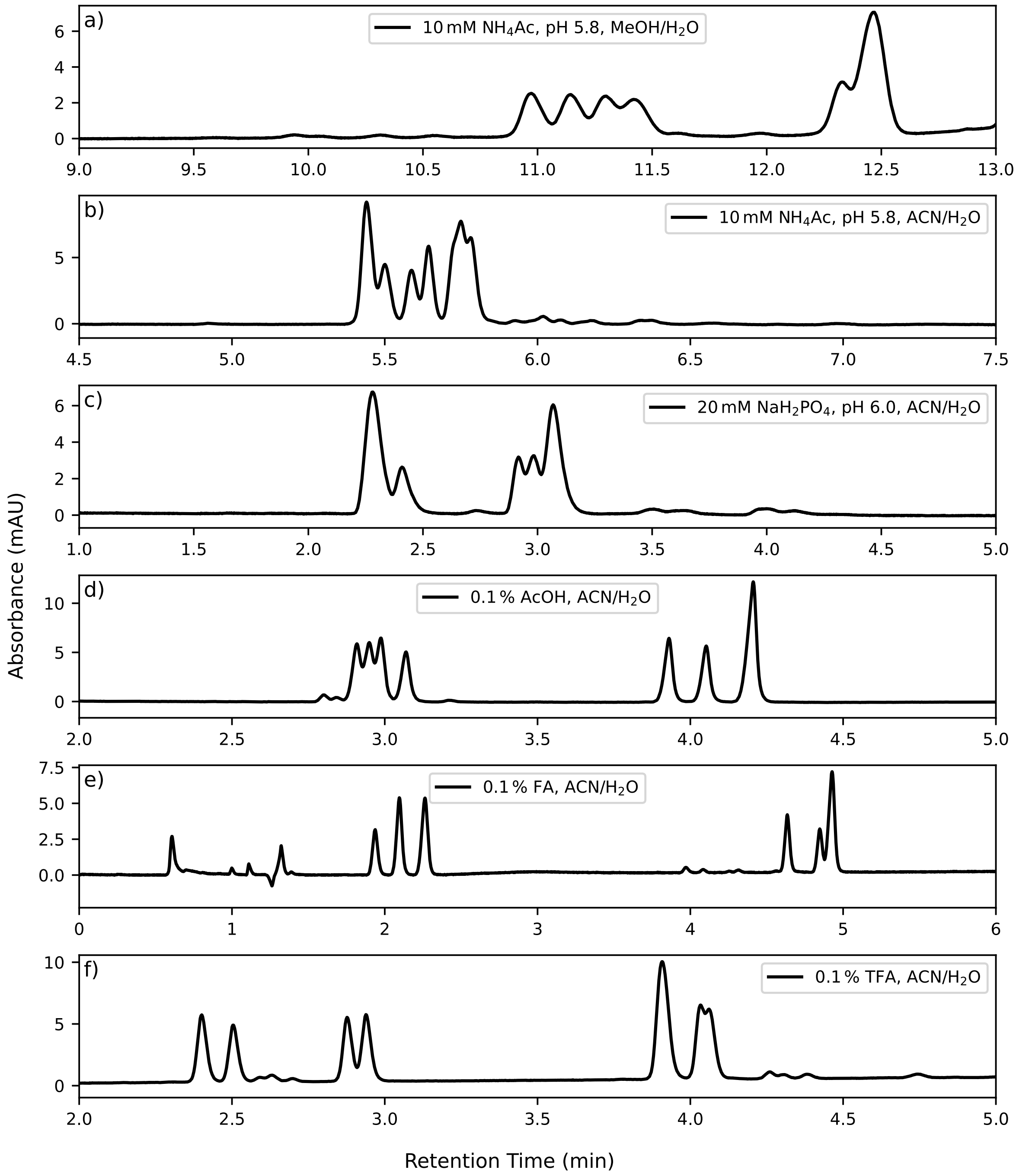

Figure S8. 1D achiral separations with the Waters Atlantis Premier BEH C18 AX.

1D achiral separations of the 16-isomer standard mix of the tetrapeptide of epifadin with the Waters Atlantis Premier BEH C18 AX (2.1 x 50 mm, 2.5 µm). Methods: a) 11, b) 12, c) 13, d) 14, e) 15, f) 16.

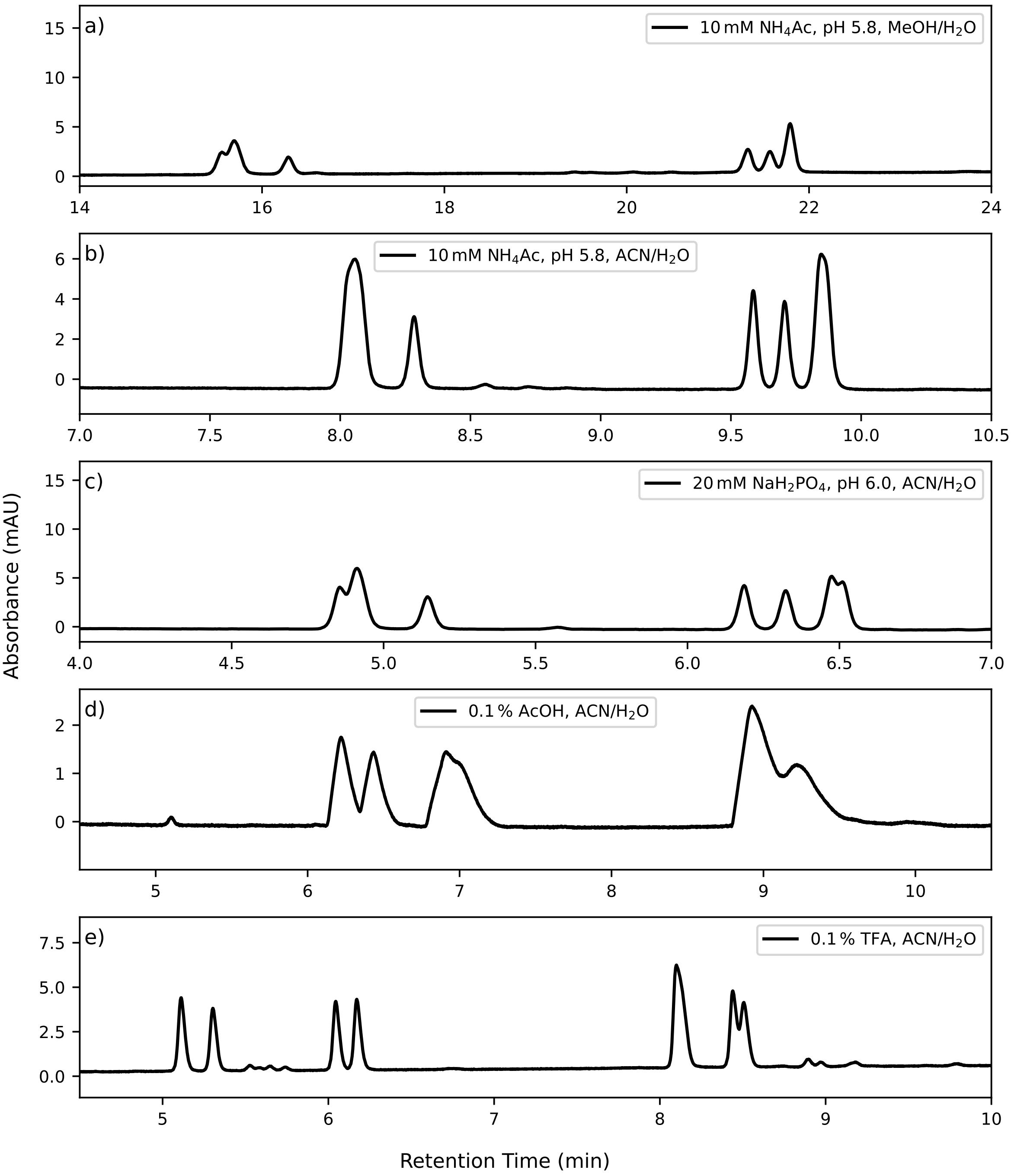

Figure S9. 1D achiral separations with Agilent Zorbax SB-C18.

1D achiral separations of the 16-isomer standard mix of the tetrapeptide of epifadin with the Agilent Zorbax SB-C18 (2.1 x 100 mm, 1.8 µm). Methods: a) 6, b) 7, c) 8, d) 9, e) 10.

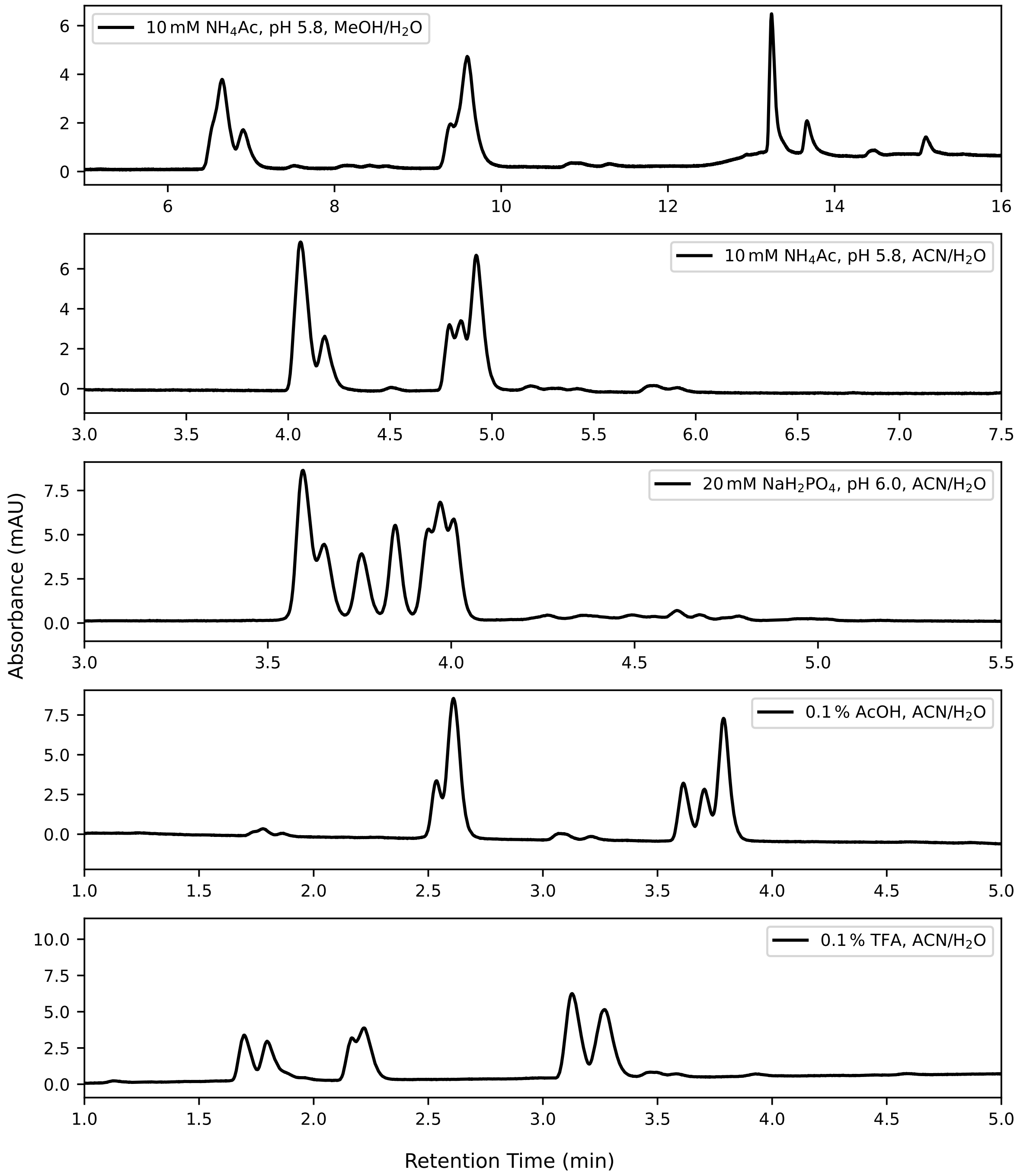

Figure S10. 1D achiral separations with the Agilent AdvanceBio Peptide Plus.

1D achiral separations of the 16-isomer standard mix of the tetrapeptide of epifadin with the Agilent AdvanceBio Peptide Plus (2.1 x 50 mm, 2.5 µm). Methods: a) 1, b) 2, c) 3, d) 4, e) 5

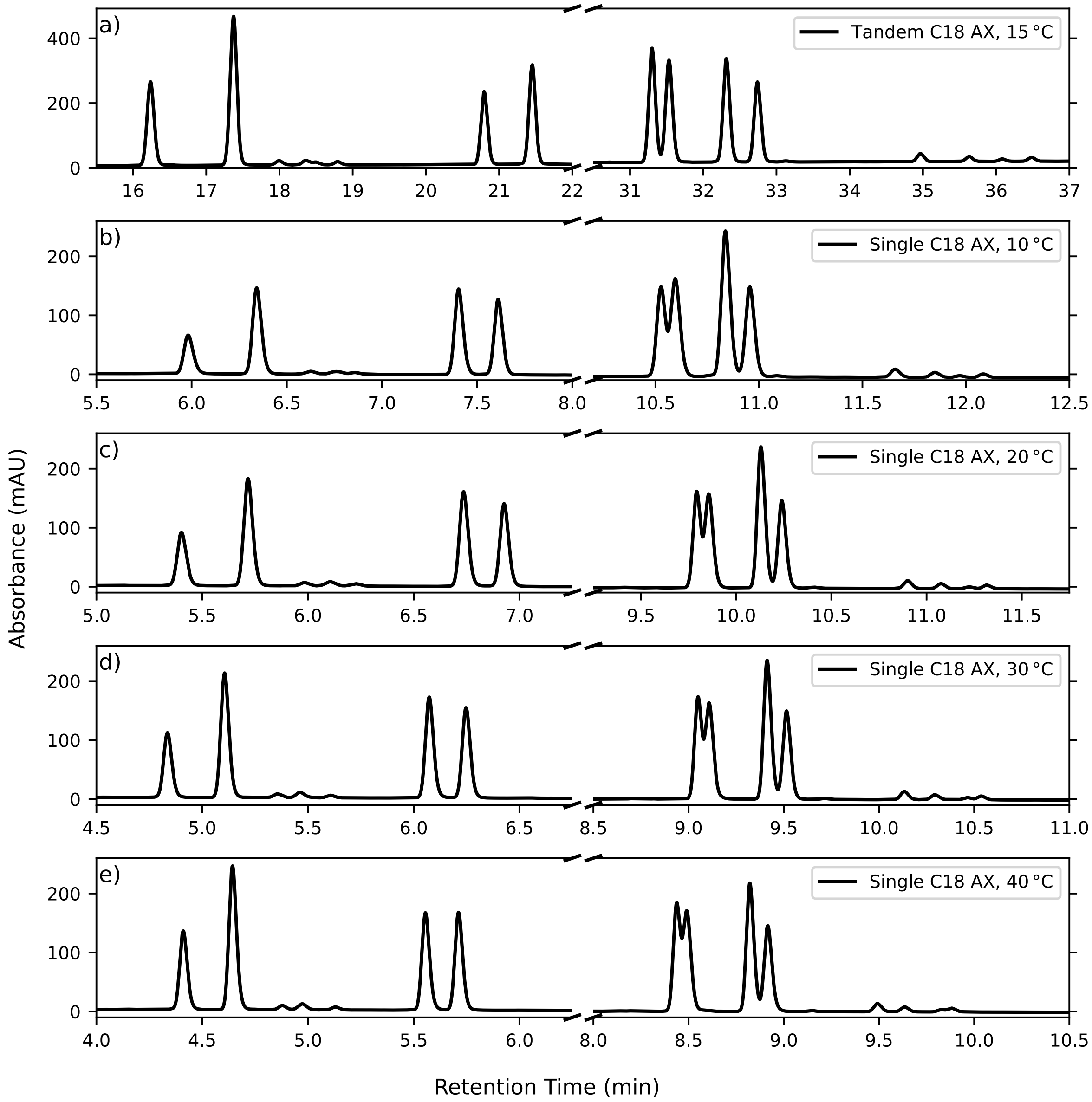

Table S11. Comparison between Single vs Tandem Column and Temperature.

Premier BEH C18 AX, 2.1x150mm, 1.7 µm. Methods: a) 23, b) 24, c) 25, d) 26, e) 27.

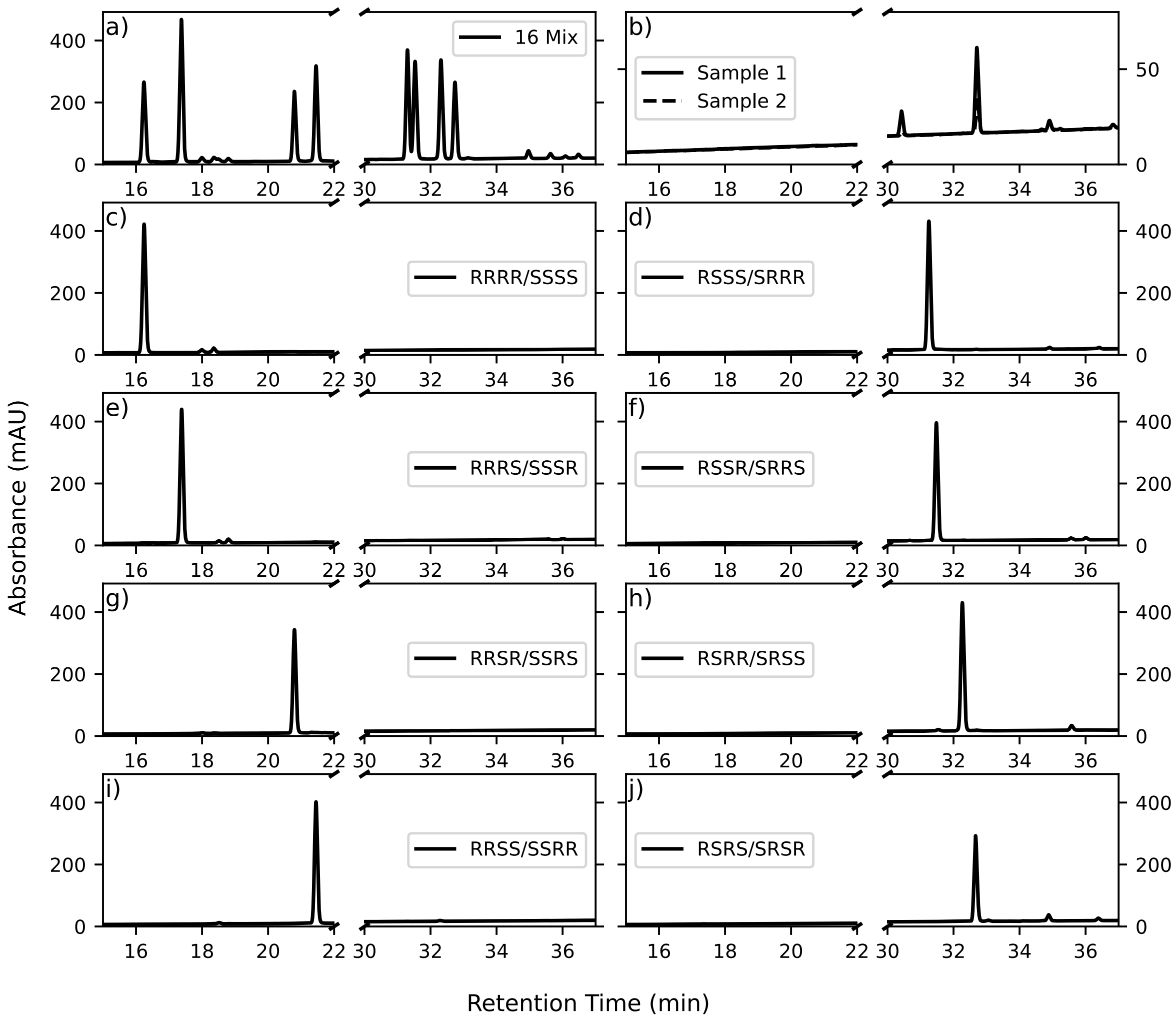

Figure S12. 1D achiral summary.

Summary of the separation of the tetrapeptide standards of epifadin with the tandem BEH C18 AX column (2 times 2.1 x 150, 1.7 µm, method: 23). Two Samples, both produced by nasal Staphylococcus epidermidis IVK83, can be assigned to the RSRS/SRSR standard/isomers.

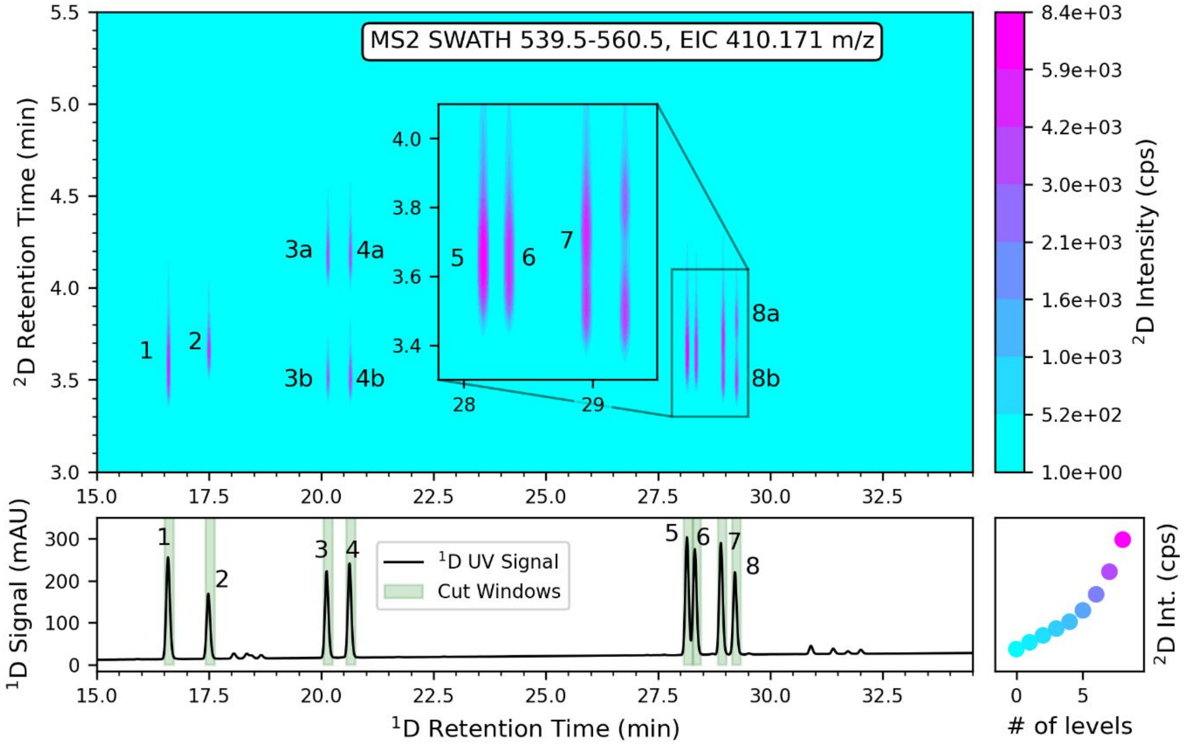

Figure S13. 2D plot with BEH C18AX and ZWIX (-).

2D plot of the 16-isomer standard mix of the tetrapeptide of epifadin. In the 1D the tandem BEH C18 AX and in the 2D the ZWIX (-) was used (Method: 35). Lower left plot shows the achiral 1D. Upper left plot shows the 2D plot with zoom area. Upper right plot de-scribes the intensity of 2D plot. Lower right shows the scaling of the color levels, first linear then exponential.

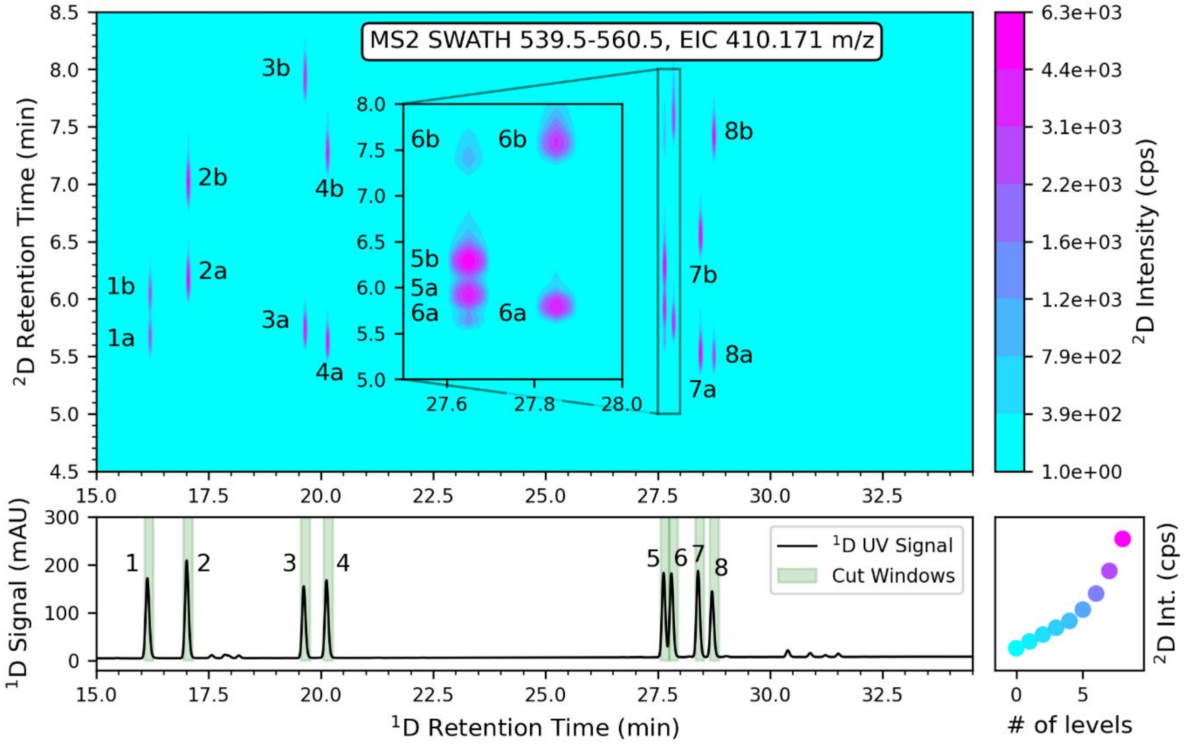

Figure S14. 2D plot with BEH C18AX and ZWIX (+).

2D plot of the 16-isomer standard mix of the tetrapeptide of epifadin. In the 1D the tandem BEH C18 AX and in the 2D the ZWIX (+) was used (Method: 34). Lower left plot shows the achiral 1D. Upper left plot shows the 2D plot with zoom area. Upper right plot de-scribes the

intensity of 2D plot. Lower right shows the scaling of the color levels, first linear then exponential.

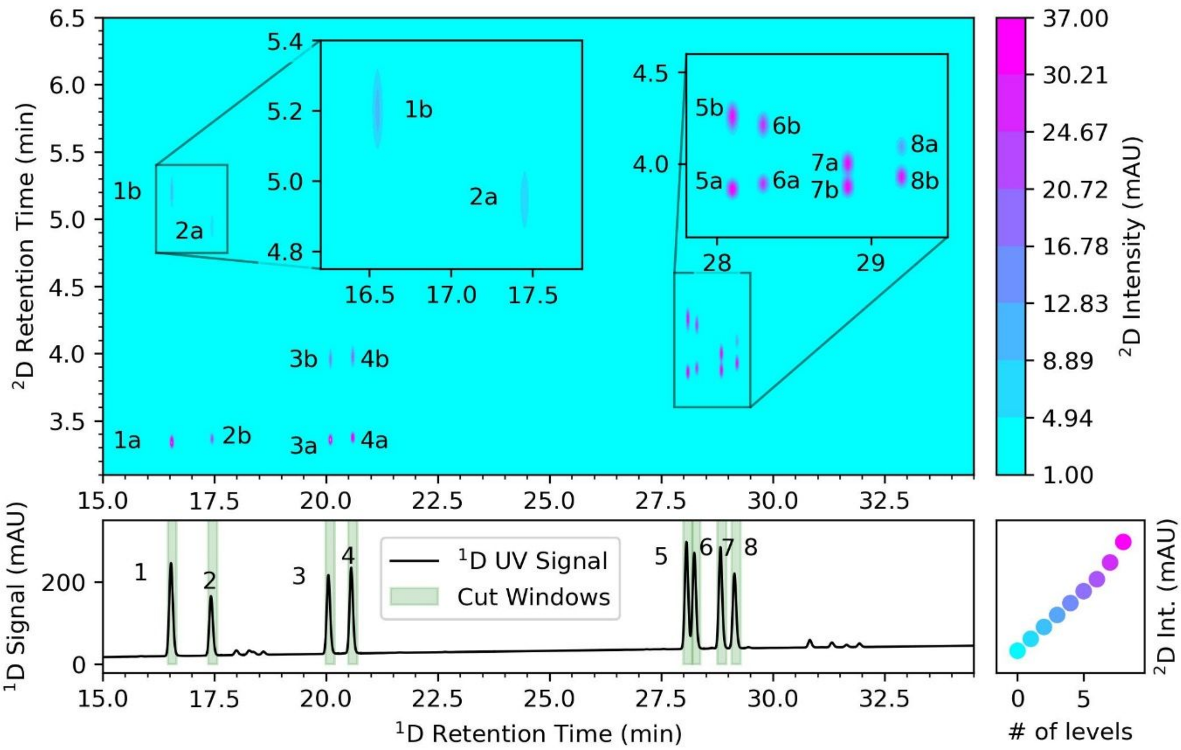

Figure S15. 2D plot with BEH C18AX and CR-I (+).

2D plot of the 16-isomer standard mix of the tetrapeptide of epifadin. In the 1D the tandem BEH C18 AX and in the 2D the CR-I (+) was used (Method: 36). Lower left plot shows the achiral 1D. Upper left plot shows the 2D plot with zoom area. Upper right plot de-scribes the intensity of 2D plot. Lower right shows the scaling of the color levels, first linear then exponential.

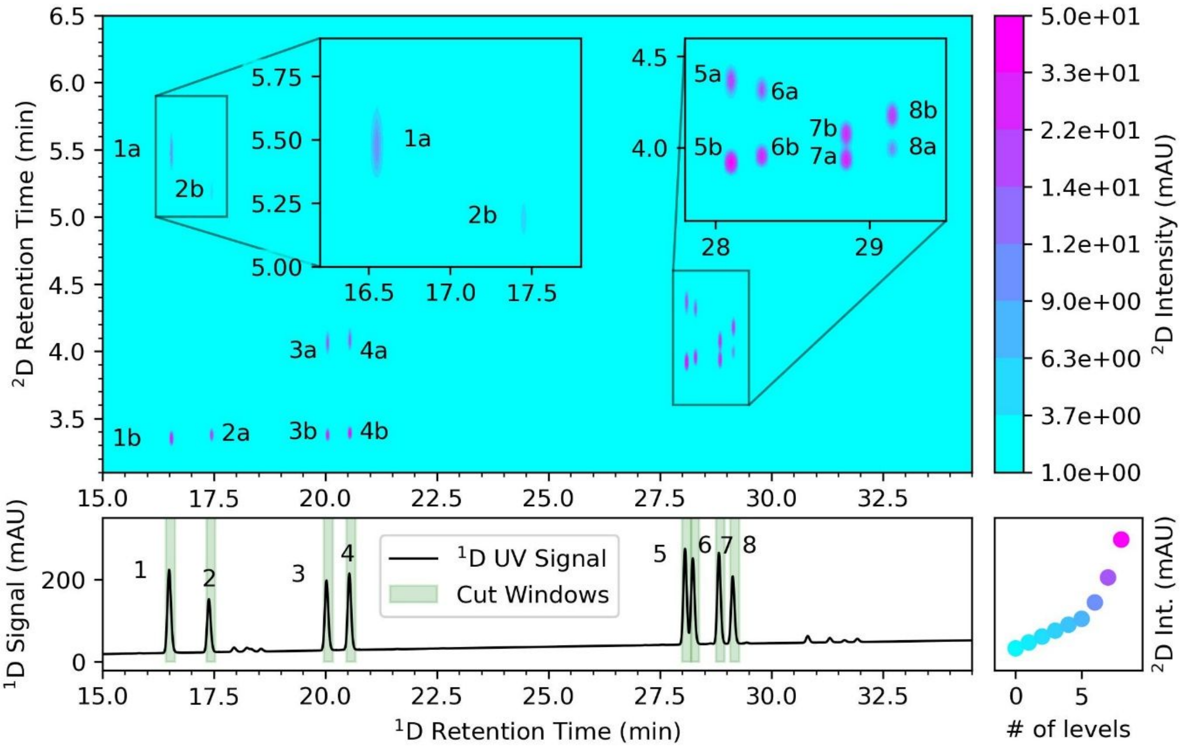

Figure S16. 2D plot with BEH C18AX and CR-I (-).

2D plot of the 16-isomer standard mix of the tetrapeptide of epifadin. In the 1D the tandem BEH C18 AX and in the 2D the CR-I (-) was used (Method: 37). Lower left plot shows the achiral 1D. Upper left plot shows the 2D plot with zoom area. Upper right plot de-scribes the intensity of 2D plot. Lower right shows the scaling of the color levels, first linear then exponential.

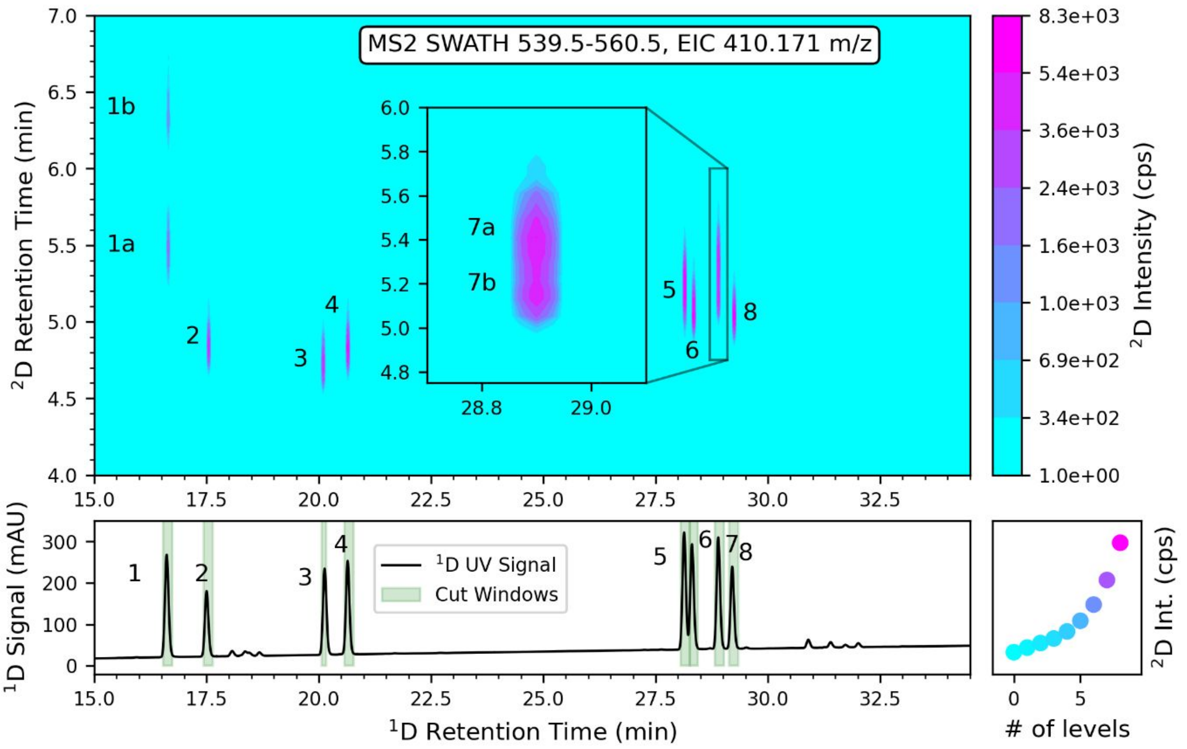

Figure S17. 2D plot with BEH C18AX and Chirobiotic T.

2D plot of the 16-isomer standard mix of the tetrapeptide of epifadin. In the 1D the tandem BEH C18 AX and in the 2D the Chirobiotic T was used (Method: 38). Lower left plot shows the achiral 1D. Upper left plot shows the 2D plot with zoom area. Upper right plot de-scribes the intensity of 2D plot. Lower right shows the scaling of the color levels, first linear then exponential.

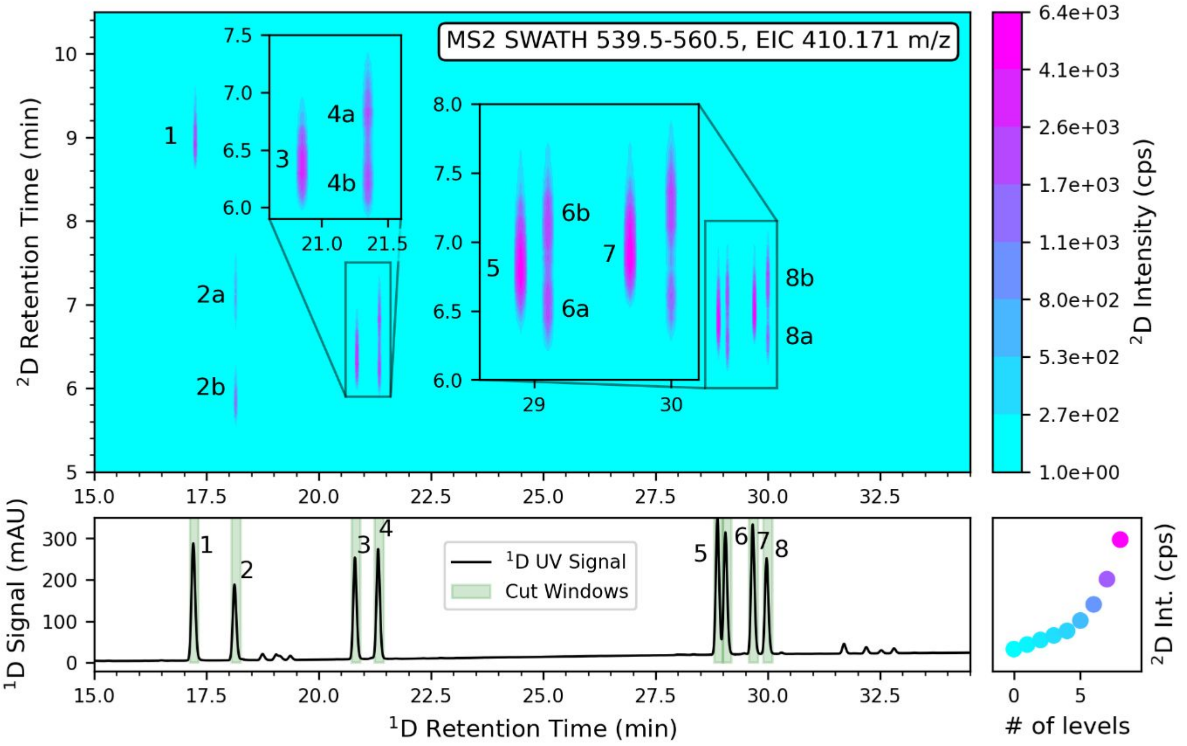

Figure S18. 2D plot with BEH C18AX and Chirobiotic TAG.

2D plot of the 16-isomer standard mix of the tetrapeptide of epifadin. In the 1D the tandem BEH C18 AX and in the 2D the Chirobiotic TAG was used (Method: 39). Lower left plot shows the achiral 1D. Upper left plot shows the 2D plot with zoom area. Upper right plot de-scribes the intensity of 2D plot. Lower right shows the scaling of the color levels, first linear then exponential.

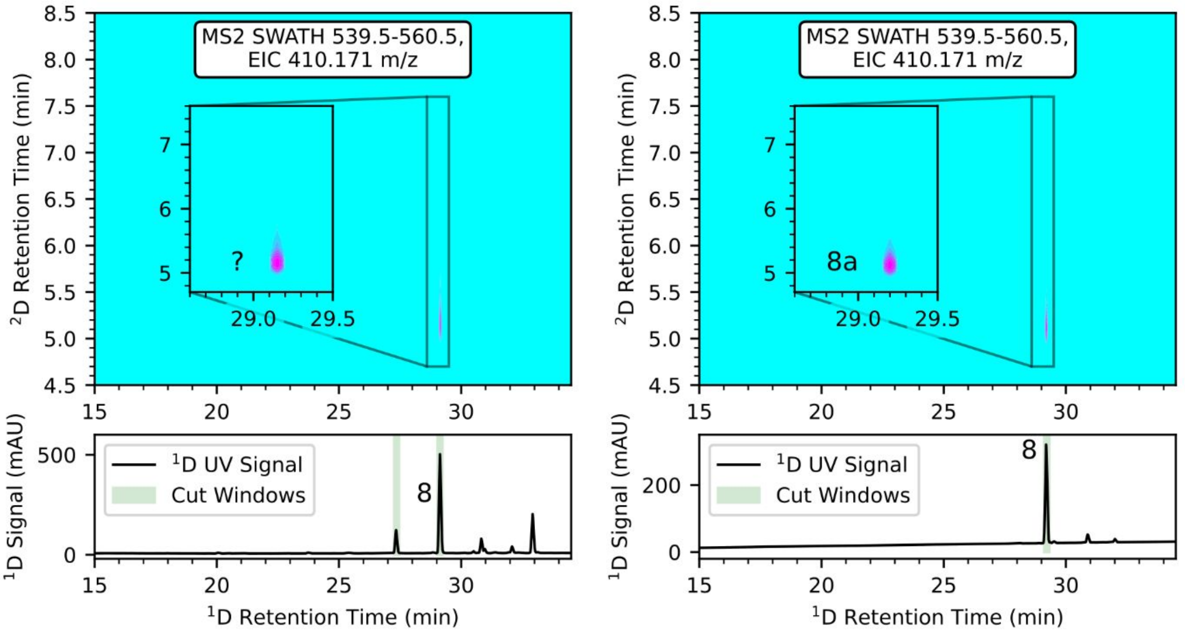

Figure S19. 2D plot comparison of sample and SRSR standard.

2D-LC-MS/MS analysis of sample produced by nasal Staphylococcus epidermidis IVK83, and the standard of the tentative *SRSR* isomer. (a) Epifadin-derived peptide sample. (b) 2D-plot of tentatively identified *RSRS*-stereoisomer. Conditions: see Table S5, method 34. b_3 fragment ion (*m/z* 410.171) of MS2 level used for generation of EIC 2D-plot.

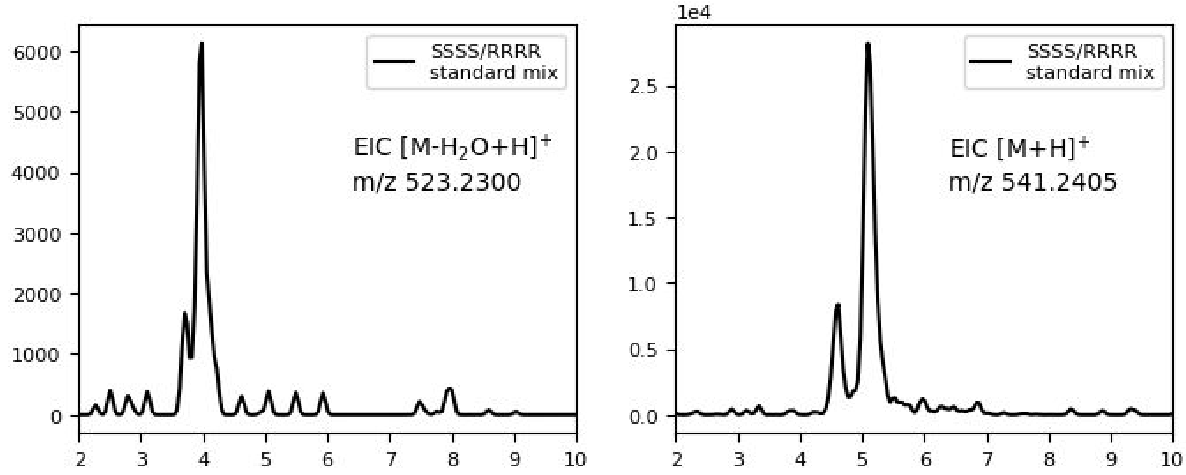

Figure S20. 1D LC-MS degradation analysis with ZWIX (+).

1D-LC-MS separation of the tetrapeptide and formed degradation or isomerization products with ZWIX(+). Method 28 was used. Left chromatogram show EIC of [M-H_2O+H]$^+$ and right chromatogram shows EIC [M+H]$^+$.

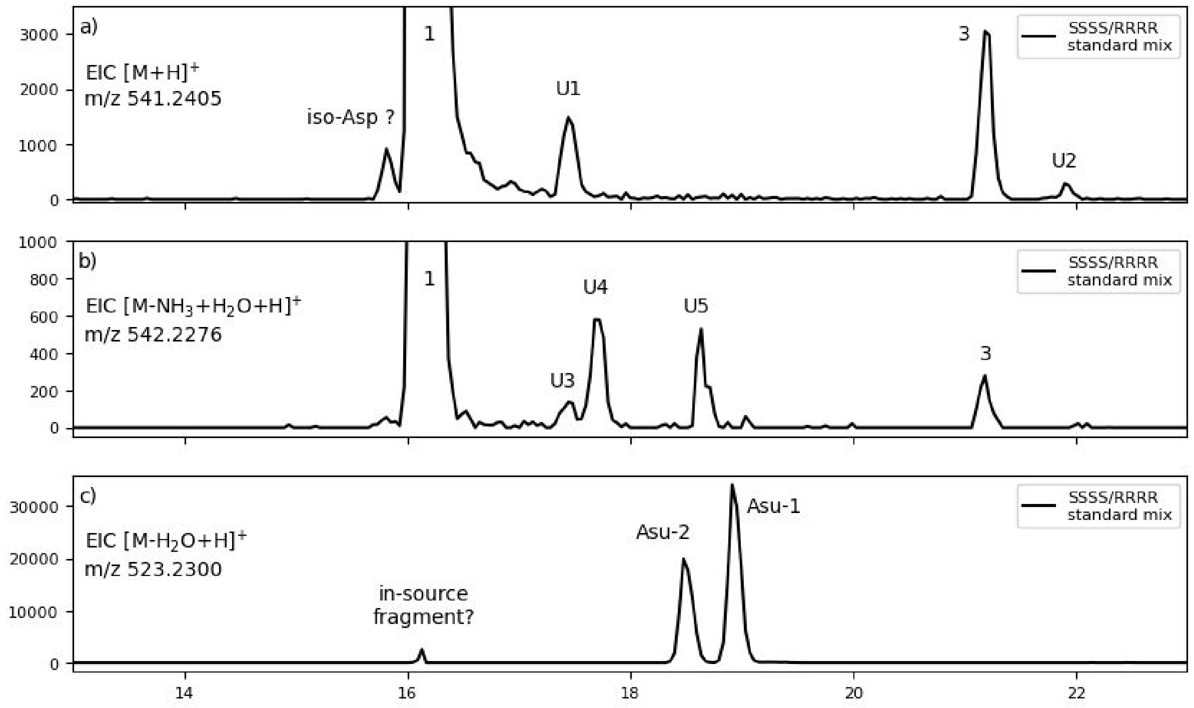

Figure S21. 1D LC-MS degradation analysis with tandem C18 AX of *SSSS*/*RRRR* standard mix.

1D-LC-MS separation of *RRRR*/*SSSS* standard mix and formed degradation or isomerization products with tandem BEH C18 AX. Method 25 with 25 °C was used. EIC shows [M-NH_3+H_2O+H]$^+$ at *m/z* 542.228. Labels: 1 *RRRR*/*SSSS*, 3 *SSRS*/*RRSR*, U1-U5 unidentified peaks.

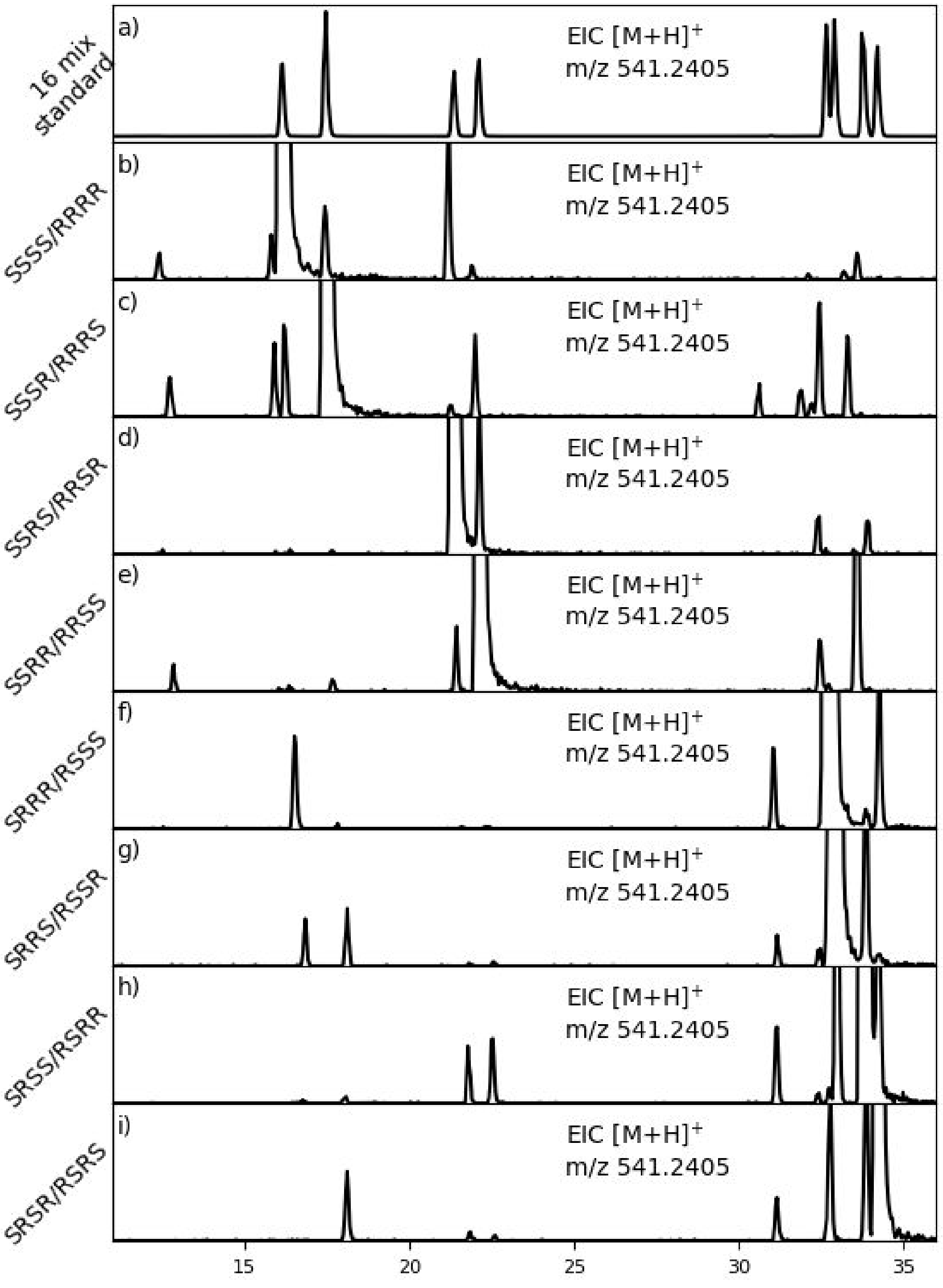

Figure S22. 1D LC-MS degradation overview of analysis with BEH C18 AX.

1D-LC-MS separation of the tetrapeptide and formed degradation or isomerization products with BEH C18 AX of all isomers. Method 25 with 25 °C was used. EIC shows $[M\text{-}NH_3\text{+}H_2O\text{+}H]^+$ at *m/z* 542.228.

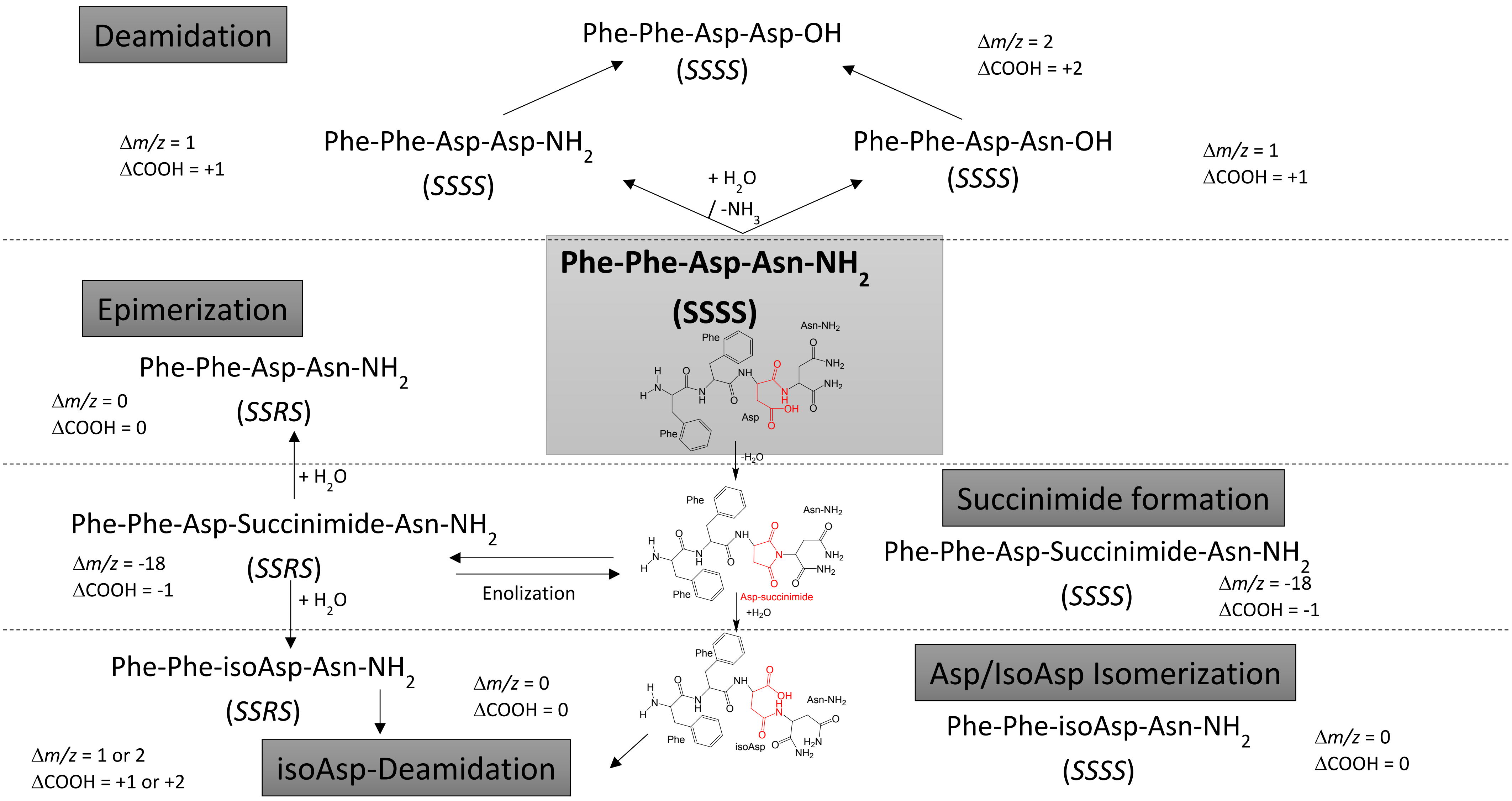

Figure S23. Possible degradation or isomerization pathways of the tetrapeptide.

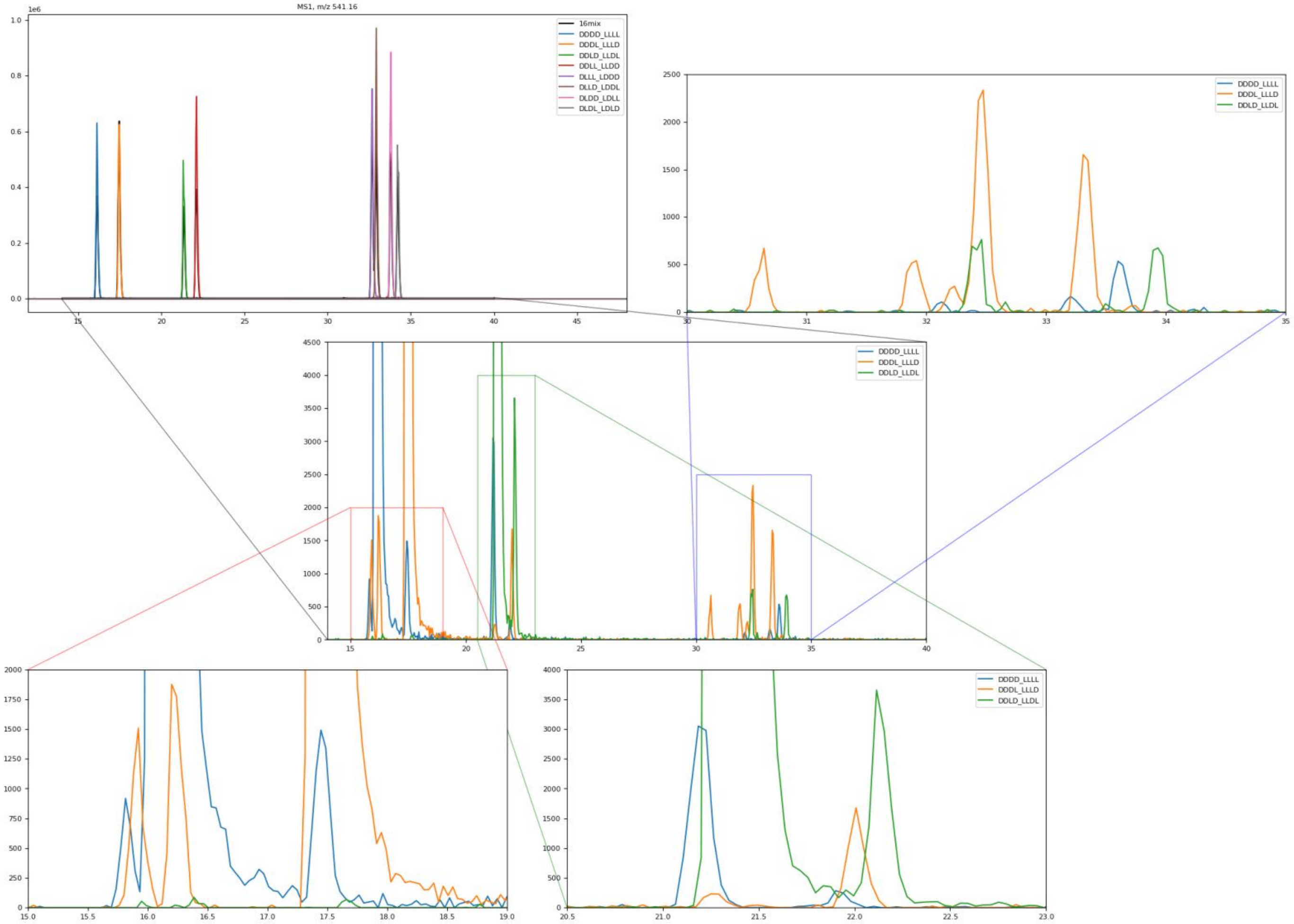

Figure S24. 1D LC-MS degradation analysis with tandem BEH C18 AX, MS1 541 *m/z*.

1D-LC-MS separation of the tetrapeptide and formed degradation or isomerization products with BEH C18 AX. Method 25 with 25 °C was used. EIC shows $[M+H]^+$ at *m/z* 541.241.

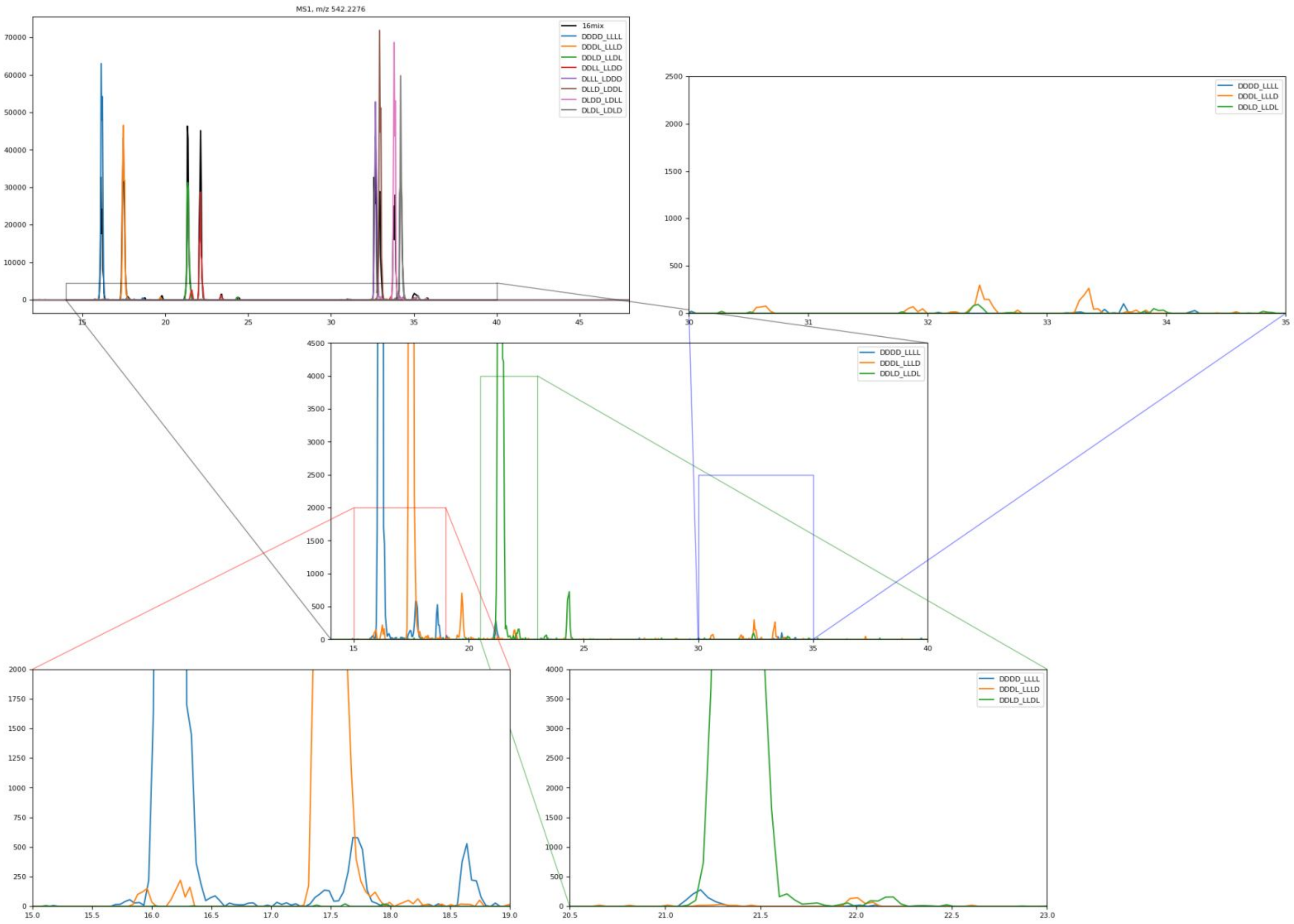

Figure S25. 1D LC-MS degradation analysis with tandem BEH C18 AX, MS1 542 *m/z*.

1D-LC-MS separation of the tetrapeptide and formed degradation or isomerization products with BEH C18 AX. Method 25 with 25 °C was used. EIC shows $[M\text{-}NH_3+H_2O+H]^+$ *m/z* 542.228.

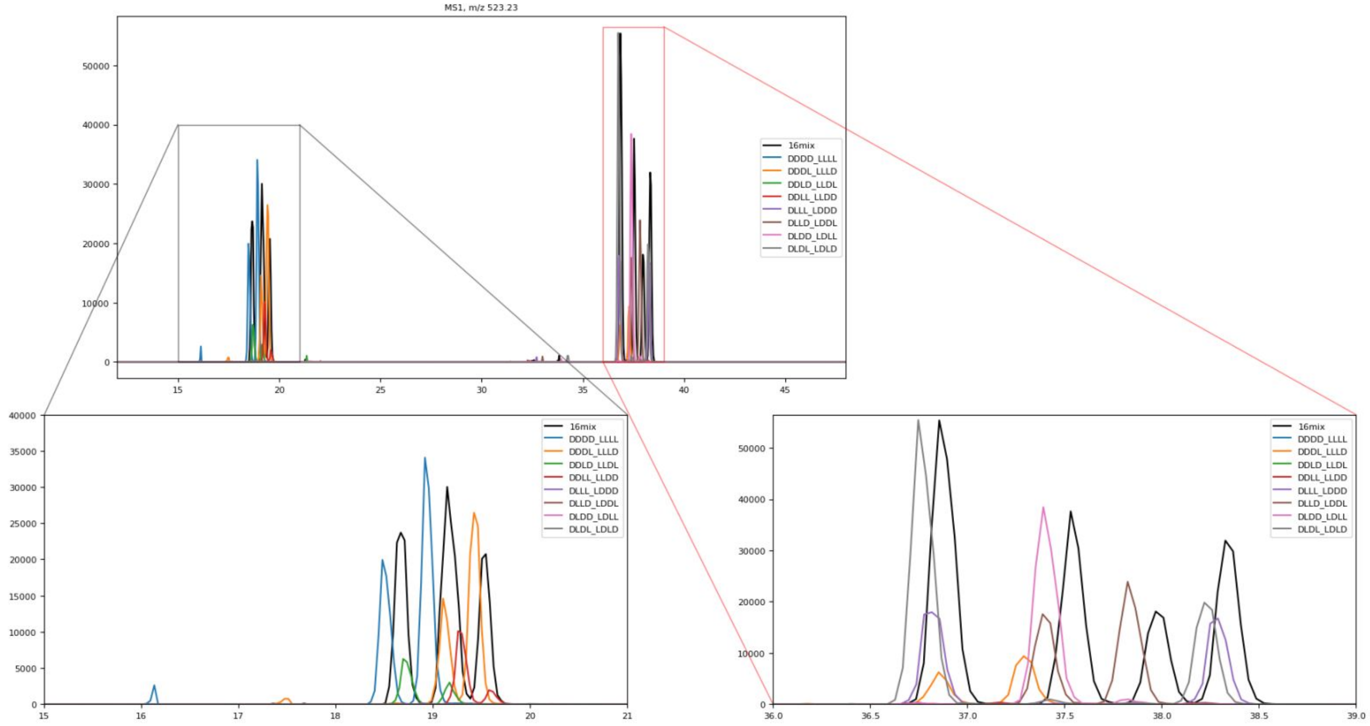

Figure S26. 1D LC-MS degradation analysis with tandem BEH C18 AX, MS1 523 *m/z*.

1D-LC-MS separation of the tetrapeptide and formed degradation or isomerization products with BEH C18 AX. Method 25 with 25 °C was used. EIC shows $[M-H_2O+H]^+$ with *m/z* 523.230.

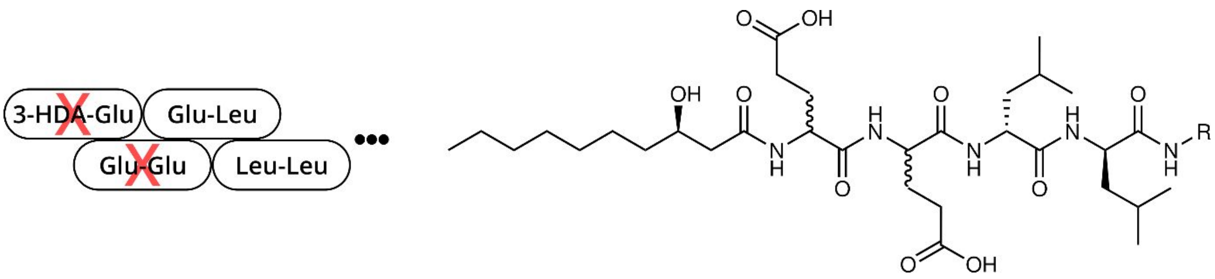

Figure S27. Partial structure and building blocks of lipopeptide.

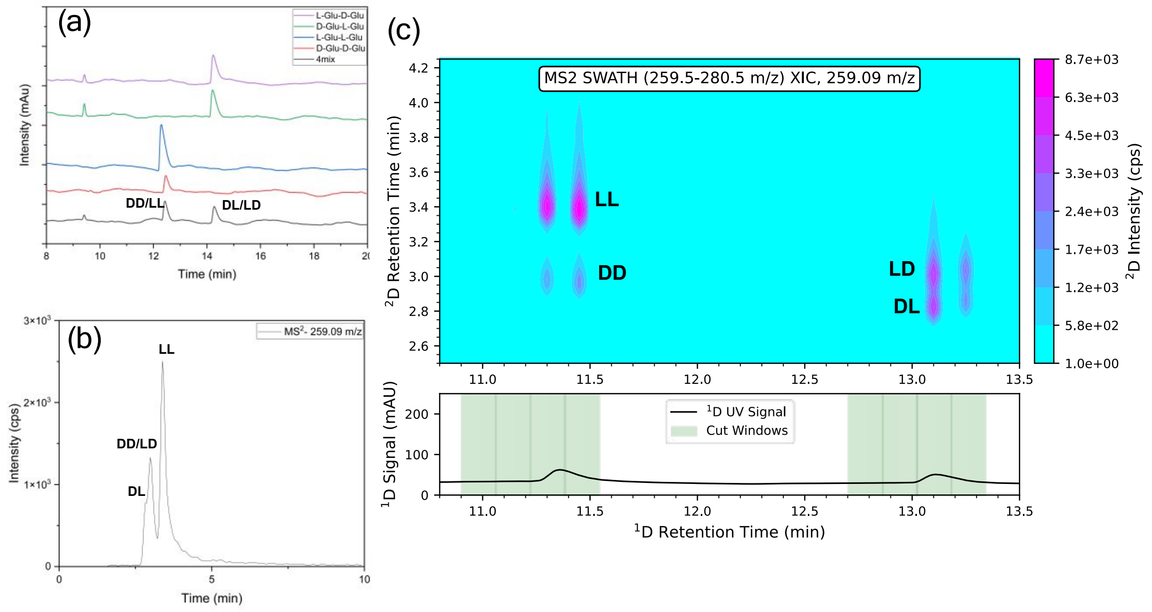

Figure S28. Separation of Glu-Glu.

1D-LC separation of Glu-Glu stereoisomers by RP18/AX (a) and Chiralpak ZWIX(+) (b), and 2D-LC plot for Glu-Glu stereoisomer mixture (c). Conditions (a) Column: Tandem column RP BEH C18 AX (2.1x150 mm, 1.7 µm); Column Temp: 25 °C; flow rate: 0.2 mL/min; Gradient: 0%-31.5% in 60 min, 90 % B for 37 min, re-equilibration 10 % B for 37 min; A: 100 % H_2O + 0.1 % AcOH (v/v); B: 100 % ACN + 0.1 % AcOH (v/v); (b) Column: Chiralpak ZWIX(+) (3x150 mm, 3 µm); Column Temp: 25 °C; flow rate: 1.0 mL/min; Gradient: 0% B for 0.66 min, 100 % B for 9.34 min.; A: 98% ACN, 2% H_2O, 5 mM FA, 2.5 mM NH_3 ; B: 98% MeOH, 2 % H_2O, 18 % MeOH, 50 mM FA, 25 mM NH_3; (c) ^{1}D as (a) and ^{2}D as (b).

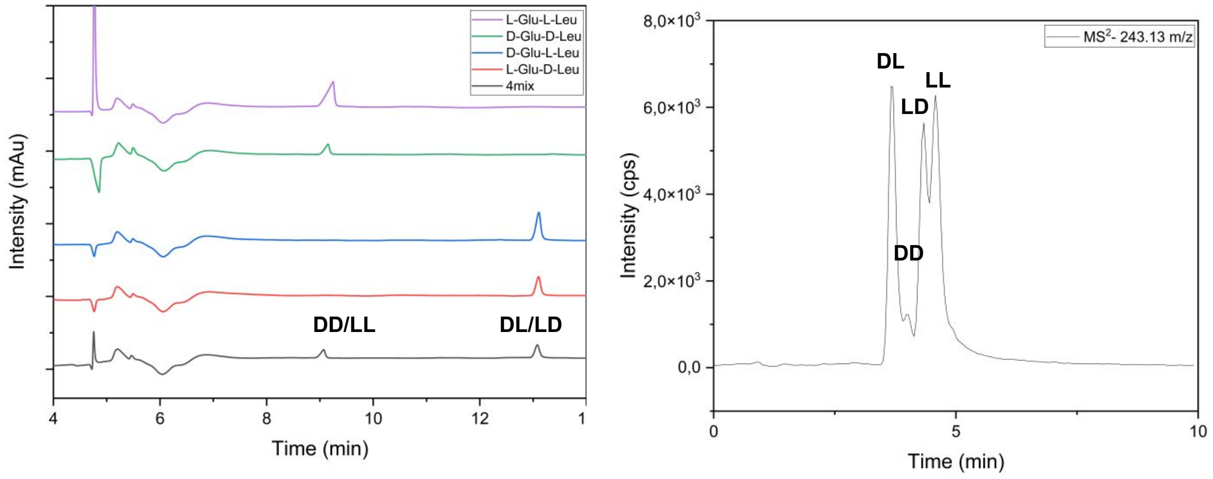

Figure S29. 1D-LC separation of Glu-Leu standards.

(a) on achiral column RP18/AX and (b) chiral column. Conditions: (a) Column: Tandem Column BEH C18 AX (2.1x100mm, 1.9µm); flow rate: 0.2 mL/min; Gradient: 0%-31.5% in 60 min, 90 % B for 37 min, re-equilibration 10 % B for 37 min; A: 100 % H_2O + 0.1 % AcOH (v/v); B: 100 % ACN + 0.1 % AcOH (v/v); (b) Column: Chiralpak ZWIX(+) (3x150 mm, 3µm); Column Temp: 25 °C; flow rate: 1.0 mL/min; Gradient: 0% B for 0.66 min, 100 % B for 9.34 min. A: 98% ACN+2% H_2O,+5 mM FA + 2.5 mM NH3 ; B: 49% ACN + 2 % H_2O+ 49 % MeOH+ 50 mM FA+ 25 mM NH_3.

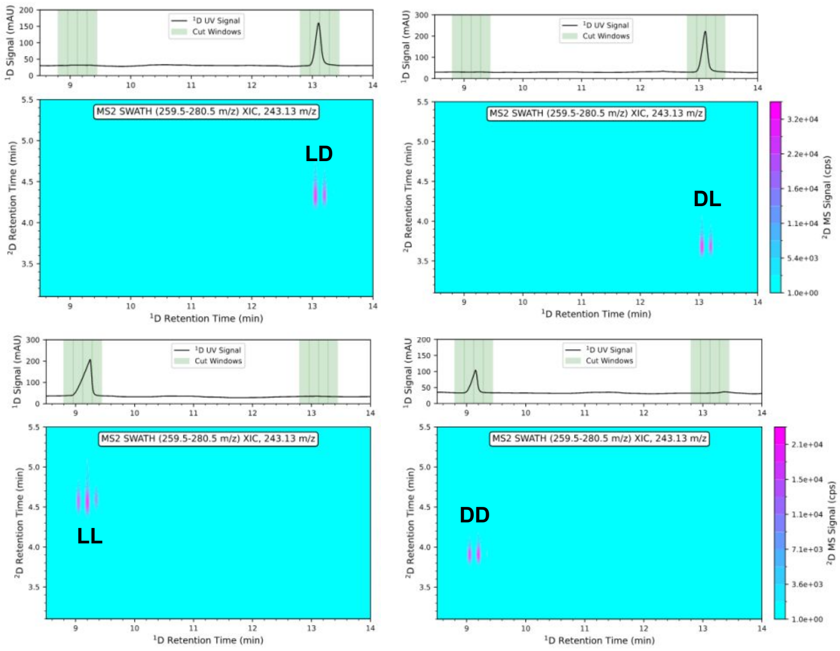

Figure S30. 2D plot of Glu-Leu.

2D-Plot for individual Glu-Leu single stereoisomer standards. Conditions see Fig S27.

Supplementary References:

(1) Virtanen, P.; Gommers, R.; Oliphant, T. E.; Haberland, M.; Reddy, T.; Cournapeau, D.; Burovski, E.; Peterson, P.; Weckesser, W.; Bright, J.; et al. SciPy 1.0: fundamental algorithms for scientific computing in Python. *Nat. Meth.* **2020**, *17*, 261–272.

(2) Harris, C. R.; Millman, K. J.; van der Walt, S. J.; Gommers, R.; Virtanen, P.; Cournapeau, D.; Wieser, E.; Taylor, J.; Berg, S.; Smith, N. J.; et al. Array programming with NumPy. *Nature* **2020**, *585*, 357–362.

(3) team, T. p. d. pandas-dev/pandas: Pandas. In *pandas-dev/pandas: Pandas*, Zenodo: 2024.

(4) Hunter, J. D. Matplotlib: A 2D Graphics Environment. *Computing in Science and Engineering* **2007**, *9*, 90–95.

(5) Shen, H. Interactive notebooks: Sharing the code. *Nature* **2014**, *515*, 151–152.
